# Global translational impacts of the loss of the tRNA modification t^6^A in yeast

**DOI:** 10.15698/mic2016.01.473

**Published:** 2015-12-18

**Authors:** Patrick C. Thiaville, Rachel Legendre, Diego Rojas-Benítez, Agnès Baudin-Baillieu, Isabelle Hatin, Guilhem Chalancon, Alvaro Glavic, Olivier Namy, Valérie de Crécy-Lagard

**Affiliations:** 1 Department of Microbiology and Cell Science, University of Florida, Gainesville, FL 32611, USA.; 2 Genetics and Genomics Graduate Program, University of Florida, Gainesville, FL 32610, USA.; 3 University of Florida Genetics Institute, University of Florida, Gainesville, FL 32610, USA.; 4 Institut de Biologie Intégrative de la Cellule (I2BC), CEA, CNRS, Université Paris-Sud, Bâtiment 400, 91400 Orsay, France.; 5 Centro de Regulación del Genoma. Facultad de Ciencias – Universidad de Chile, Santiago, Chile.; 6 Laboratory of Molecular Biology, Francis Crick Avenue, Cambridge CB2 0QH, United Kingdom.

**Keywords:** t6A, tRNA, ribosome profiling, translation, modified nucleosides

## Abstract

The universal tRNA modification t^6^A is found at position 37 of nearly all tRNAs decoding ANN codons. The absence of t^6^A_37_ leads to severe growth defects in baker’s yeast, phenotypes similar to those caused by defects in mcm^5^s^2^U_34_ synthesis. Mutants in mcm^5^s^2^U_34_ can be suppressed by overexpression of tRNA^Lys^_UUU_, but we show t^6^A phenotypes could not be suppressed by expressing any individual ANN decoding tRNA, and t^6^A and mcm^5^s^2^U are not determinants for each other’s formation. Our results suggest that t^6^A deficiency, like mcm^5^s^2^U deficiency, leads to protein folding defects, and show that the absence of t^6^A led to stress sensitivities (heat, ethanol, salt) and sensitivity to TOR pathway inhibitors. Additionally, L-homoserine suppressed the slow growth phenotype seen in t^6^A-deficient strains, and proteins aggregates and Advanced Glycation End-products (AGEs) were increased in the mutants. The global consequences on translation caused by t^6^A absence were examined by ribosome profiling. Interestingly, the absence of t^6^A did not lead to global translation defects, but did increase translation initiation at upstream non-AUG codons and increased frame-shifting in specific genes. Analysis of codon occupancy rates suggests that one of the major roles of t^6^A is to homogenize the process of elongation by slowing the elongation rate at codons decoded by high abundance tRNAs and I_34_:C_3_ pairs while increasing the elongation rate of rare tRNAs and G_34_:U_3_ pairs. This work reveals that the consequences of t^6^A absence are complex and multilayered and has set the stage to elucidate the molecular basis of the observed phenotypes.

## INTRODUCTION

**Figure 1:**
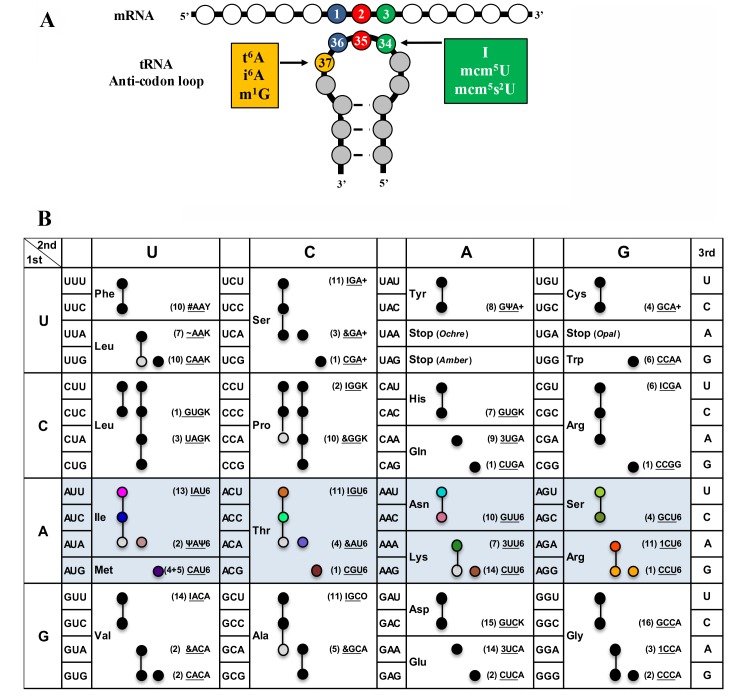
FIGURE 1: (A) Complex modifications found in the anticodon stem loop (ASL) of tRNA. (B) Codon table with decoding tRNAs, based on Johansson *et al*. 47. Blue highlighted cells are decoded by t^6^A modified tRNAs in *S. cerevisiae*. In parenthesis is the number of genomic copies of that tRNA followed by the anticodon (underlined) and base at position 37. Black, grey, and colored circles indicate a codon decoded by that tRNA predicted by the wobble hypothesis, with grey indicating a tRNA less likely to decode that codon, and colors are matching those in Figure 6 and 7. For AUG, four genes code for tRNA^iMet^, and five code for tRNA^eMet^. Modification symbols are from Modomics 44. Ψ – pseudouridine, & - ncm^5^U, I – inosine, 3 – mcm^5^s^2^U, 1 – mcm^5^U, # – Gm, ~ – ncm^5^Um, Y – wybutosine, K – m^1^G, 6 – t^6^A, + – i^6^A, and O – 1-methylinosine.

Modifications of the anticodon stem loop (ASL) of transfer RNA (tRNA) are critical for translational speed and accuracy. As the genetic code is degenerate, most tRNAs decode several codons 1. Nucleoside modifications ensure that the decoding process is stringent enough to discriminate between closely-related codons and yet relaxed enough to allow decoding of more than one codon 2,3. Different organisms use distinct but convergent strategies to optimize speed and accuracy of decoding by modifying specific tRNAs, predominantly at position 34 (the wobble base) and at position 37 (the dangling base) of the ASL (Figure 1A) 2,3. Modifications at position 34, such as ncm^5^U_34_ (5-carbamoylmethyluridine) or I_34_ (inosine) can expand decoding capacity, thereby allowing one tRNA to decode three synonymous codons 2,3,4. Likewise, most modifications at position 37 are critical for decoding, and their role can be complex. For example, i^6^A_37_ (N6-isopentenyladenosine) promotes decoding activity and increases fidelity of tRNA^Cys^_GCA_ at its cognate codon, but also increases the misreading rate of tRNA^Tyr^_GUA_ at the near-cognate UGC codon, which makes the effects of this modification on protein expression difficult to disentangle 5. In fact, the exact *in vivo* contributions of many ASL modifications to translational robustness are still poorly understood 6.

Codon usage bias allows for fine-tuning of translation by ASL modifications. Codon choice (which codon in a synonymous set is used to encode a given amino acid) affects gene expression levels, protein production, accuracy, protein folding 7,8,9,10, and can even be used to predict gene function 11. Codon usage bias is not only driven by neutral processes such as mutation biases or GC%, but is also molded by selection 9,12. Although translation speed is a strong driving force of codon usage 9, the avoidance of codons with higher propensity for protein synthesis errors leading to misfolding is also an important factor in codon selection 13,14. ASL modifications play key roles in both these processes 6,7. In addition, systems level approaches integrating proteomics, codon usage, and modification profiling 15 have recently shown that tRNA modifications can modulate the expression of specific genes including stress-responsive genes 16,17. These tRNA modification tunable transcripts (MoTTs) respond to the proportion of modified tRNAs and regulate translation in response to cellular stress 18. 

Threonyl-carbamoyl-adenosine (t^6^A) is a complex universal modification found at position 37 of nearly all ANN decoding tRNAs, as shown in Figure 1B (for an in depth review of t^6^A synthesis in all domains of life, see Thiaville *et al.* 19). t^6^A is formed in a two-step mechanism, where, in the cytoplasm of eukaryotes, the threonyl-carbamoyl-AMP (TC-AMP) intermediate is produced by Tcs1 or Tcs2 (previously named YrdC and Sua5, respectively) 20,21,22. TC-AMP is placed on tRNA by the threonyl-carbamoyl transferase complex (TCTC, previously referred to as KEOPS or EKC complex) made up of Tcs3 (Kae1), Tcs5 (Bud32), Tcs6 (Pcc1), and Tcs7 (Cgi121), whereas fungi have an additional member Tcs8 (Gon7)] 23,24,25. Yeast mitochondria use a minimum synthesis system to produce t^6^A-modified tRNAs, consisting of a mitochondrial-targeted Tcs2 and Tcs4 (Qri7) 26,27. 

In yeast, the absence of t^6^A synthesis enzymes has been linked to many phenotypes including telomere shortening 28,29, transcription regulation defects 30, and respiration deficiency 31,32,33. The molecular basis for these pleiotropic phenotypes is far from understood, although it is expected that they should relate to translational defects in absence of t^6^A. In addition to the aberrant mis-initiation observed in the *TCS2* mutant when the gene was discovered 34, the deletion of the *TCS2* and *TCS3* results in an increase in +1 and -1 frameshifts, as well as to mis-initiation at CUG codons of specific reporter genes 20,23. Further studies linked loss of *TCS2* with increases in leaky scanning bypass of start codons, +1 frameshifts, read-through of UAG, UAA, and UGA stop codons, and an increase in internal ribosome entry site translation (IRES-dependent initiation of translation) 33. Polysome profiles of *TCS2*-depleted strains (P_TET_::*TCS2*, this strain requires doxycycline for expression of *TCS2*) revealed abnormal ribosome assembly, which could not be rescued by over-expressing the ternary complex (TC; eIF2α, -β, -γ, and Met-tRNA^iMet^), contrary to previously reported cases of ribosome assembly defects caused by inhibition of other tRNA modifications 33. Similarly, the over-expression of either TC or tRNA^iMet^ (*IMT4*) did not rescue the slow-growth phenotype in absence of t^6^A 33. However, depletion of *TCS2* leads to increased levels of the transcriptional activator *GCN4*, although in a non-canonical manner (Gcd^–^ phenotype) 33. *GCN4* is a positive regulator of genes expressed during amino-acid starvation, and is dependent on eIF2α phosphorylation by Gcn2, which monitors uncharged tRNAs 35,36. Over-expression of tRNA^iMet^ or deletion of *GCN2* did not reduce the high levels of *GCN4* in a *TCS2*-depletion background 33. Paradoxically, *GCN4* induction in the *TCS2*-depleted strain was independent of Gcn2 phosphorylation 33. In yeast, Gcn4 is also regulated at the translational level by four upstream open reading frames (uORFs), where the scanning ribosome initiates translation at the first AUG in the uORF leading to bypass of initiation at the AUG of the downstream ORFs 37. *TCS2*-depletion led to increased translation of the main ORF (*GCN4*) by bypassing the regulatory uORFs 33. Over-expression of TC or tRNA^iMet^ did not reduce the leaky scanning seen in *TCS2*-depletion 33. Interestingly, mutations of Tcs3, Tcs5, and Tcs8 in yeast also increased *GCN4* translation 38.

Evidence has emerged that some tRNA modifications can act as determinants of subsequent tRNA modification enzymes. Recently, the requirements of 2’-*O*-methylation of C_32_ and N_34_ has been linked to efficient wybutosine formation at m^1^G_37_ of tRNA^Phe^, a circuitry conserved from yeast to man 39,40,41. Additionally, in bacteria, presence of the t^6^A modification increases the efficiency of formation of the essential modification lysidine at U_34_ of tRNA^Ile^_CAU_, and t^6^A is required for the charging of tRNA^Ile^ by IleRS 42,43. In yeast, parallels can also be made between t^6^A and 5-methoxycarbonylmethyliouridine (mcm^5^U) and its thiolated derivative (mcm^5^s^2^U) found at position 34 of several tRNAs. Both t^6^A and mcm^5^s^2^U modify tRNA^Lys^_UUU,_ and mcm^5^U and t^6^A are found on tRNA^Arg^_UCU_ 44. Trm9 and Elp1-6 (Elongator complex) synthesise the mcm^5^ moiety, and the Ncs2/Ncs6 enzymes are responsible for thiolation 45,46. Deficiencies in mcm^5^s^2^U synthesis lead to slow growth, the inability to grow on non-fermentable carbon sources, and telomere shortening 45,47,48,49,50,51, which are similar to phenotypes seen in t^6^A biosynthesis mutants 20,23,29,33,34,38,52,53. Over-expression of a single tRNA, tRNA^Lys^_UUU_, suppresses all of the mcm^5^s^2^U phenotypes, and additional data suggest that mcm^5^s^2^U acts as a codon-dependent regulator of translation 52,53. Why elimination of mcm^5^s^2^U or t^6^A lead to similar phenotypes is unknown 2. One possibility is that the modification of A_37_ to t^6^A is required for the formation of the x^5^s^2^U derivatives, or vice versa, which has never been explored to date. 

Recently, both mcm^5^s^2^U and t^6^A have been associated to alterations of two central cell regulatory systems; the General Amino Acid Control system (GAAC) through activation of *GCN4* 38,54, which regulates > 1500 genes in response to nutritional cues 35, and Target of Rapamycin Complex (TORC), through alterations in Tor kinase activity 55,56,57,58 (reviewed in Thiaville and de Crécy-Lagard 59). Modulating the levels of t^6^A in *Drosophila* through expression of an unmodifiable tRNA^iMet^ or overexpression of *TCS3* led to alterations of Tor activity and changes in whole organism growth 56. Additionally, knock-down of Tcs3 (Kae1) or Tcs5 (Bud32) in *Drosophila* larvae activated the Unfolded Protein Response (UPR) 55. 

Recent ribosome profiling studies of mutations in the mcm^5^s^2^U pathway (*ncs6*∆ and *uba4*∆) grown under nutrient-depleted conditions revealed pausing and accumulation of ribosomes at GAA, AAA, and CAA codons 54. Follow up studies also found codon-specific ribosome pausing in the absence of mcm^5^s^2^U (*ncs2*∆*elp6*∆), even in the absence of stress 60. Hypo-modified tRNAs cause slower decoding at GAA, AAA, and CAA codons that led to protein misfolding and aggregation of essential proteins, which prevent the cell from maintaining protein homeostasis during stressful events 60. 

In this study, we sought to uncover the translational defects seen in a t^6^A deficient strain and to determine if there is a relationship between mcm^5^s^2^U and t^6^A. So far, the links between t^6^A and translation fidelity have been based on single gene reporters. Here, we sought to assess the functional impact of t^6^A at the genome scale, by combining genome-wide ribosome profiling and bioinformatics tools to catalogue all translational ambiguities in *tcs2*∆ and investigate the differential effects of t^6^A on distinct tRNAs. 

## RESULTS

### mcm^5^s^2^U_34_ or t^6^A_37_ are not determinants for each other’s synthesis

The similarity of the phenotypes observed in strains deficient in mcm^5^s^2^U and t^6^A synthesis suggests that one of the modifications could be required for the synthesis of the other. To test this hypothesis, tRNAs from wild type (BY4741), mcm^5^s^2^U-deficient yeast strains (*elp3*∆, *trm9*∆, *ncs2*∆, and *ncs6*∆) and t^6^A synthesis mutants (*tcs2*∆-*tcs8*∆) were purified and analysed by HPLC. 

**Figure 2:**
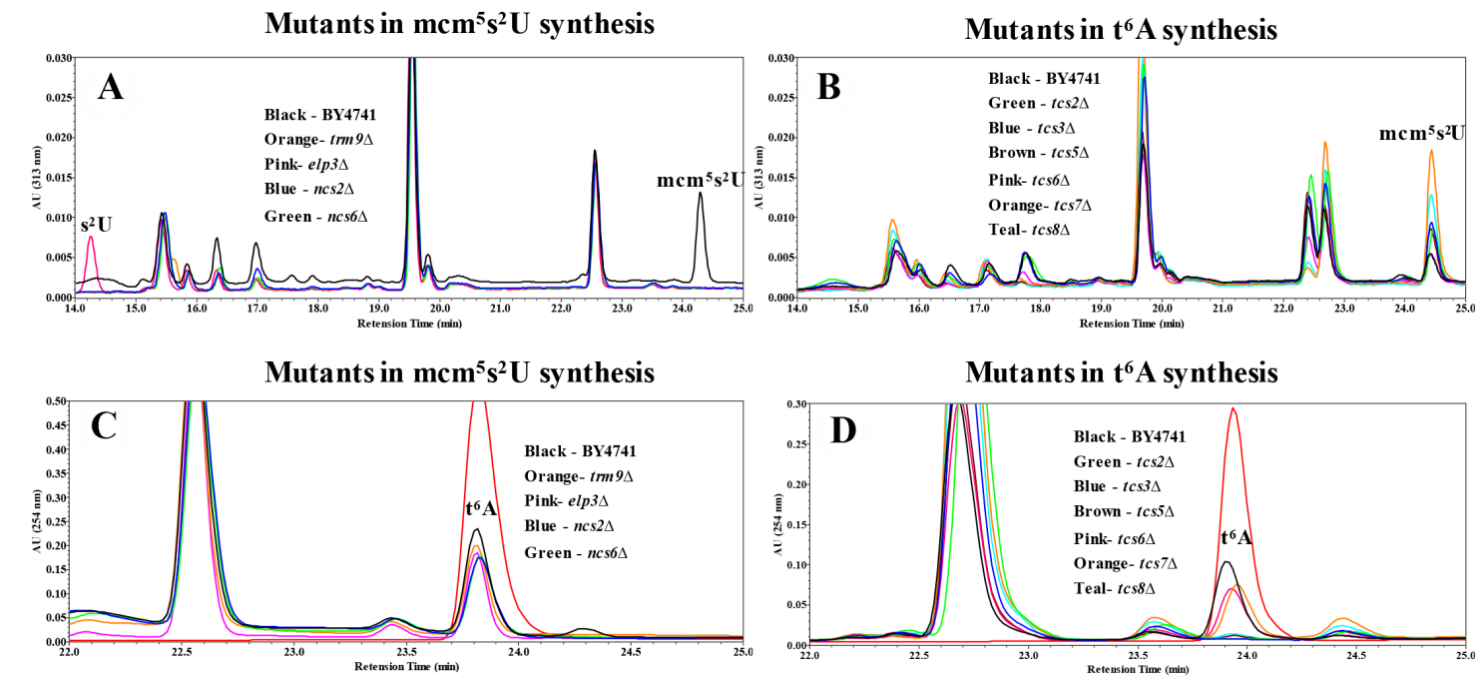
FIGURE 2: HPLC analysis examining the relationship between mcm^5^s^2^U_34_ and t^6^A_37_. **(A) **Analysis of mutations in mcm^5^s^2^U synthesis with detection at 313 nm specific for thio-moieties. Black line = BY4741; Orange = *trm9*∆; Pink = *elp3*∆; Blue = *ncs2*∆; Green = *ncs6*∆. **(B)** Analysis of mcm^5^s^2^U in mutants for t^6^A synthesis for with detection at 313 nm. Black = BY4741; Green = *tcs2*∆; Blue = *tcs3*∆; Brown = *tcs5*∆; Pink = *tcs6*∆; Orange = *tcs7*∆; Teal = *tcs8*∆. **(C) **Analysis for t^6^A in mutants of mcm^5^s^2^U synthesis with detection at 254 nm. The color scheme is the same as part A, with the t^6^A standard in red. **(D) **Analysis for t^6^A in mutants of t^6^A synthesis with detection at 254 nm. Color scheme is the same as part B, with the t^6^A standard in red.

To determine how t^6^A synthesis deficiency affects mcm^5^s^2^U, HPLC analysis with detection at 313 nm (for detection of thio moieties) of nucleosides of tRNAs purified from *elp3*∆*, trm9*∆*, ncs2*∆*, *and* ncs6*∆ revealed the mcm^5^s^2^U peak at 24.35 minutes, which was unique to BY4741 but absent in all the mutants, and a peak at 14.20 minutes appeared only in *elp3*∆, indicating the presence of the s^2^U moiety in this strain (Figure 2A). The chromatographic patterns match previously published reports 50. Analysis of tRNAs purified from t^6^A biosynthesis mutants (*tcs2*∆*-tcs8*∆) revealed that all strains possessed the peak at 24.35 minutes corresponding to mcm^5^s^2^U, and none of the mutants showed the s^2^U peak at 14.20 minutes (Figure 2B). Interestingly, most mutants in t^6^A synthesis had higher levels of mcm^5^s^2^U (*tcs6*∆ is unchanged) as compared to BY4741, among which *tcs7*∆ was the highest (Figure 2B). 

The HPLC profile at 254 nm revealed that all the mutants in mcm^5^s^2^U synthesis contained the same amount of t^6^A as the parental BY4741 strain, as indicated on Figure 2C by a peak at 23.57 minutes. Analysis of the t^6^A synthesis mutants (*tcs2*∆-*tcs8*∆) confirmed prior results of the absence of t^6^A in *tcs2*∆ and *tcs3*∆ 20,23, and revealed the absence of t^6^A in both *tcs5*∆ and *tcs8*∆ (Figure 2D). *tcs6*∆ and *tcs7*∆ were reduced for t^6^A relative to wild type by ~20% (Figure 2D), very similar to the reduction seen in a *tcs6*∆ mutant in the archaea *Haloferax volcanii* 61. These results indicate that mcm^5^s^2^U_34_ and t^6^A_37_ do not require one another for their synthesis, although eliminating t^6^A did increase levels of mcm^5^s^2^U. 

### Overexpression of tRNAs or Ternary Complex (TC) do not suppress the growth defects of *tcs2*∆

**Figure 3:**
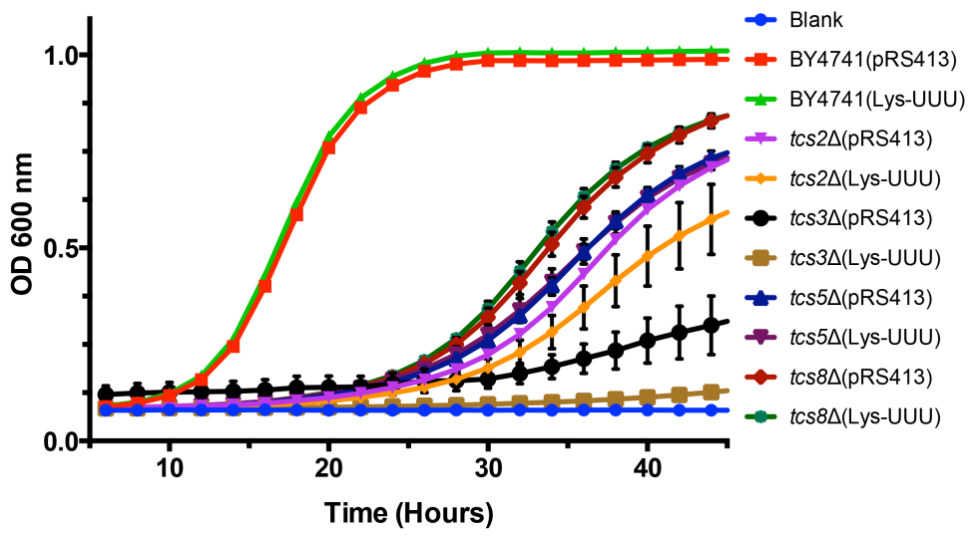
FIGURE 3: Expression of tRNA^Lys^_UUU_ does not suppress slow growth of mutants devoid of t^6^A. BY4741, *tsc2*Δ, *tsc3*Δ, *tsc5*Δ, and *tsc8*Δ were transformed with plasmids expressing tRNA^Lys^_UUU_ or empty vector. Data points are the average of 8 biological replicates. Error bars represent standard error of the mean (SEM).

Overexpression of tRNA^Lys^_UUU_ is sufficient to suppress all the phenotypes resulting from mutations of mcm^5^s^2^U synthesis enzymes 53. Therefore, we tested if this was also the case for mutations in the t^6^A synthesis pathway. To assess if tRNAs could suppress the slow growth rate seen in mutants of t^6^A synthesis, an expression plasmid containing tRNA^Lys^_UUU_ was transformed into BY4741, *tcs2*∆, *tcs3*∆, *tcs5*∆, and *tcs8*∆ (*tcs6*∆ and* tcs7*∆ were not tested as they do not have a growth defect). Unlike in the case of mcm^5^s^2^U, the expression of tRNA^Lys^_UUU_ did not suppress the growth defect observed in t^6^A synthesis mutants (Figure 3). Interestingly, it led instead to a small reduction in growth rate in the mutants (Figure 3). Further, we expressed the other tRNAs that decode ANN codons (tRNA^Lys^_CUU_, tRNA^eMet^_CAU_, tRNA^Ile^_AAU_, tRNA^Ile^_UAU_, tRNA^Thr^_UGU_, tRNA^Arg^_ACG_, tRNA^Arg^_UCU_, tRNA^Arg^_CCU_, and tRNA^Glu^_UUC_ (does not contain t^6^A and decodes GAA, which is the most frequently used codon in *S. cerevisiae*)) in *tcs2*∆. None, of these individual tRNAs suppressed the growth defect of *tcs2*∆ (data not shown). To confirm the results of *TCS2*-depletion published by Lin *et al*. 33, plasmids over-expressing tRNA^iMet^, eIF2α, or TC were transformed into BY4741 and *tcs2*∆. In agreement with the previous results 33, neither tRNA^iMet^, eIF2α, nor TC suppressed the slow growth of *tcs2*∆ (Figure S2A), while the growth of *tcs2*∆ can be restored by expressing *TCS2* *in trans* (Figure S2B). 

Hence, unlike the suppression of mcm^5^s^2^U by tRNA^Lys^_UUU_, neither the overexpression of each ANN-tRNA, nor the overexpression of TC components could suppress the fitness defects observed in mutants of the t^6^A biosynthesis pathway. The effects of the loss of t^6^A thus appear to be more complex than those of the loss of mcm^5^s^2^U_._

### t^6^A-deficient strains are sensitive to heat and inhibitors of TOR, but growth can be partially rescued by L-homoserine

**Figure 4:**
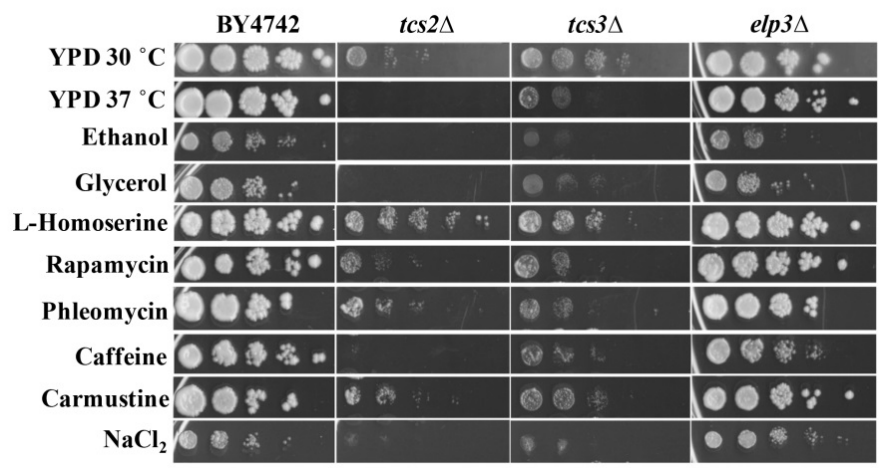
FIGURE 4: Stress phenotypes of t^6^A-deficient strains differ from a mcm^5^s^2^U-deficient strain. Cells were grown at 30°C for 48 hours on YPD with 2% glucose except when 6% ethanol or 3% glycerol was used as a sole carbon source or heat stress at 37°C. Drugs were added to YPD at the following levels: L-homoserine, 1 mg/mL; Rapamycin, 10 nM; Phleomycin, 8 µg/mL; caffeine, 10 mM; carmustine, 1 mM; NaCl_2_, 1 M.

To better characterize how the absence of t^6^A was affecting cellular function, growth on several carbon sources and under different stress conditions was tested (Figure 4). t^6^A-deficient strains were found to be sensitive to heat stress, with *tcs2*∆ unable to grow at 37°C, and to salt stress, with both *tcs2*∆ and *tcs3*∆ affected by the presence of 1 M NaCl_2_. Also, *tcs2*∆ was unable to grow on 3% glycerol or 6% ethanol, but did grow slowly on 2% glucose (YPD), while *tcs3*∆ was able to grow slowly on all carbon sources (Figure 4). Addition of inhibitors of the TOR pathway such as caffeine (10 mM) 62 or rapamycin (10 nM) further reduced the growth of t^6^A-deficient strains (Figure 4). Interestingly, the addition of L-homoserine (1 mg/ml) partially suppressed the growth defects of the *tcs2*∆ strain, but not of the *tcs3*∆ strain. Several other chemical stresses did not affect growth of t^6^A^-^ strains (Figure 4). These included the addition of DNA damaging agents such as phleomycin (8 μg/ml) or carmustine (1 mM). The *elp3*∆ strain was also tested in the same conditions as it is known that depending on the strain background, mutants in Elongator genes vary as to the degree of response to each of these stressors 63. The results presented in Figure 4 are consistent with the results recently published by the Schaffrath laboratory, reporting the lack of strong phenotypes of the *elp3*∆ strain when using the BY4741 background 64, 

Homoserine acts as a toxic threonine analogue and incorporation of homoserine activates protein degradation pathways 65. To test whether activation of UPR could suppress the growth phenotype of t^6^A-depletion strain in yeast, t^6^A- strains were transformed with plasmids expressing UPR factors Xbp1, Kar2 (GRP78/BIP), Der1, and Hrd1. Expression of UPR-related factors were unable to rescue the slow-growth phenotype seen in t^6^A-deficient strains (data not shown).

### t^6^A^- ^strains accumulate aggregated proteins and advanced glycated end-products (AGEs)

**Figure 5:**
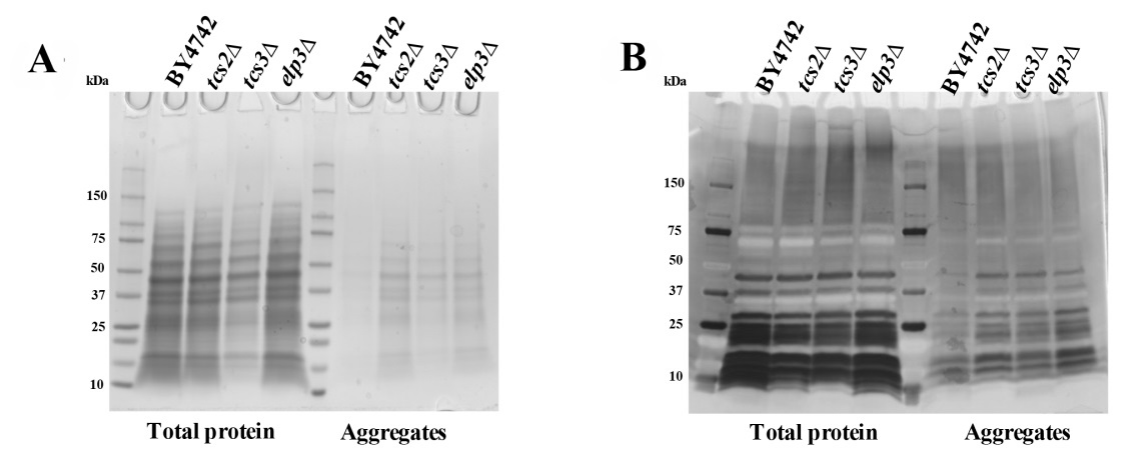
FIGURE 5: t^6^A deficient strains accumulate aggregated proteins and Advanced Glycated End-products (AGEs). **(A)**Protein aggregation in BY4742 and mutant yeast cells. Yeast were grown in YPD to an OD_600_ = 0.8. Soluble and aggregated proteins were separated by SDS–PAGE and visualized by Coomassie blue staining. **(B)** AGEs were visualized by silver staining.

Double mutants *elp6*∆*ncs2*∆ (eliminating mcm^5^s^2^U) have been shown to contain increased amount of aggregated (insoluble) proteins, possibly due to alteration in translation speed 60. Equal amounts of total and insoluble proteins from BY4742, *tcs2*∆, *tcs3*∆ and *elp3*∆ were analysed by SDS-PAGE and Coomassie staining. Depletion of t^6^A in *tcs2*∆ and *tcs3*∆ increased the amount of aggregated or insoluble proteins similar to the single *elp3*∆ strain (Figure 5A), which is less than the amount of insoluble protein seen in the double *elp6*∆*ncs2*∆ strain 60. Prior experiments in *E. coli* and *H. volcanii* revealed that AGEs become more abundant when t^6^A levels are reduced 61,66. To assess levels of AGEs in our context, equal amounts of total and insoluble proteins from BY4742, *tcs2*∆, *tcs3*∆ and *elp3*∆ were separated by SDS-PAGE and visualized with a diol-specific silver stain for glycated proteins 67. Aggregated proteins extracted from *tcs2*∆, *tcs3*∆, and *elp3*∆ were all increased in AGEs relative to wild type (Figure 5B). 

### Ribosome assembly defects are observed in the t^6^A^- ^strain

In light of the diverse phenotypes observed and because the previous analysis of translation defects had focussed on a handful of reporter proteins, we performed a global ribosome profiling analysis to assess the impact of t^6^A-deficiency at the genome scale. An essential step in ribosome profiling is ensuring high quality polysomes are prepared, which was assessed by sucrose gradient sedimentation and subsequent analysis with a fraction analyser. Polysomes prepared from *tcs2*∆ exhibited a “half-mer” phenotype, which is represented by a shoulder after the 80S peak on the chromatograph, blue arrow in Figure S3. Half-mers indicate excess 40S ribosome and incomplete assembly of the 80S particle, which may indicate problems with initiation 68. The half-mer phenotype of *tcs2*∆ was not seen in a prior publication examining a *TCS2*-depletion strain 33. These results may differ due to strain genotypes or technical differences in the preparation of the polysomes.

### The absence of t^6^A leads to increased ribosome occupancy of arginine synthesis genes

A detailed description of purification of the ribosome-protected fragments (RPFs) and sequencing can be found in material and methods. Analysis of RPFs in *tcs2*∆ revealed 111 genes were decreased in RPFs and 196 genes were increased in RPFs relative to wild type. A complete list of these genes and their functional roles can be found in Table S3 and Table S4. To determine if any functional relationship existed with these genes, we performed gene ontology (GO) enrichment using YeastMine (http://yeastmine.yeastgenome.org) 69. The pathway for arginine biosynthesis was found to be enriched, *P *= 0.049, with five genes, *ARG5,6, CPA2, ARG7, ARG1, *and* CPA1* identified. None of the arginine catabolism pathways were significantly increased (Table S4). Increased mRNA expression of arginine has been documented to act as an anti-oxidant to oxidative stress by an unknown mechanism 70. This antioxidative pathway acts through pyrroline-5-carboxylate (P5C) but *PUT1*, encoding a P5C synthesis enzyme, was not increased in RPFs in the mutant (Table S4). Of the 111 genes decreased in RPFs, five genes were identified matching the GO term polyphosphate metabolic process, *P* = 0.003, and no pathway enrichment was identified.

### Depletion of t^6^A deregulates *GCN4*

The number of genes proposed to be regulated by Gcn4 varies greatly with the specific study, from less than 500 genes (microarrays measuring gene expression during histidine starvation) 35 to more than 2500 genes (predicted computationally by SGD). The most conservative estimate of Gcn4-induced genes was produced from a ChIP-Chip assay, which found 128 genes bound during immunoprecipitation of Gcn4 71. Comparison of the RPFs detected in *tcs2*∆ with the 128 well defined Gcn4-regulated genes reveals that 15 ORFs increased in RPFs are regulated by Gcn4 in *tcs2*∆, while no ORFs decreased in RPFs in *tcs2*∆ are regulated by Gcn4, Figure S4A. The 15 Gcn4-regulated genes with increased RPFs in *tcs2*∆ are involved in amino acid synthesis, with six in the arginine synthesis pathway (*P *= 1.3 x 10^-5^), Table S5. 

Microarray analyses of conditional or point mutations in *tcs3*, *tcs6,* or *tcs8* had been previously reported 38 and in all these studies, an up-regulation of Gcn4 regulated genes was observed, including genes in the arginine and histidine biosynthesis pathways, although the mRNA expression levels of *GCN4* itself did not increase. The genes increased in each of the previous microarray studies were compared to the genes with increased RPFs in the ribosome profiling analysis of *tcs2*∆. Of the 196 genes with increased RPFs in *tcs2*∆, 29 were also increased in *tcs3-18*, 30 were also increased in *tcs6-4*, and 12 were also increased in *tcs18-ts1*, summarized in Figure S4B. 12 genes were increased in all four datasets (Table 1). 9 of these are under Gcn4 control, of which four are in the arginine synthesis pathway (Table 1).

Contrary to the previous microarray results that did not detect *GCN4 *induction, we found that RPFs mapping to *GCN4* were increased 6-fold in *tcs2*∆ (Table S4). This difference may be due to an up-regulation of translation (detected by the increased levels of RPFs in *tcs2*∆), and not transcription (as measured by the microarrays). With the 6-fold increase in *GCN4* expression, it is surprising so few Gcn4 inducible genes are increased in *tcs2*∆. Indeed, only 8% of RPFs increased in *tcs2*∆ are in common with the Gcn4p ChIP data. 

**Table 1:** Genes increased in expression in *tcs2*∆, *tcs3-18*, *tcs6-4*, and *tcs8-ts1*. *Under control of Gcn4 (see Table S5), ^&^Arginine biosynthesis.

**Systematic name**	**Standard name**	**Description**
YER069W	ARG5,6	Acetylglutamate kinase and N-acetyl-gamma-glutamyl-phosphate reductase*^&^
YER175C	TMT1	Trans-aconitate methyltransferase
YGL117W		Putative protein of unknown function
YJL079C	PRY1	Sterol binding protein involved in the export of acetylated sterols
YJR025C	BNA1	3-hydroxyanthranilic acid dioxygenase*
YJR109C	CPA2	Large subunit of carbamoyl phosphate synthetase*^&^
YMR062C	ARG7	Mitochondrial ornithine acetyltransferase*^&^
YMR095C	SNO1	Protein of unconfirmed function*
YMR096W	SNZ1	Protein involved in vitamin B6 biosynthesis*
YNL104C	LEU4	Alpha-isopropylmalate synthase (2-isopropylmalate synthase)*
YOL058W	ARG1	Arginosuccinate synthetase*^&^
YOR130C	ORT1	Ornithine transporter of the mitochondrial inner membrane*

### A discrete but not a global increase in translational ambiguity is observed in the t^6^A^- ^strain *tcs2*∆ 

In yeast, the RPF is 28 nucleotides long 72, hence to analyse the frame of each ribosome, only 28-mers that aligned uniquely to the genome and did not contain mismatches were used. 6.5 x 10^6^ reads in wild type and 5.8 x 10^6^ reads in *tcs2*∆ matched these strict criteria. For each read, the nucleotide at position +12, which corresponds to the ribosomal P-site, was determined and its identity defined the frame of the read, and hence the frame of the ribosome, Figure S5. Each ORF was divided into windows of approximately 300 nucleotides (minimum of 3 windows per ORF to a maximum of 9), and reads inside each window were mapped and enumerated 73. Since we cannot be sure if the ribosome associated with read is in frameshift or if that ribosome began translation of the ORFs out of frame, translational ambiguity is defined as the mapping of a read in a frame other than the frame of the annotated ORF. 

There are four well-documented examples of +1 frameshifts occurring in *S. cerevisiae* and these were used to evaluate the frame analysis performed here. One known +1 frameshift, *TRM140* (an AdoMet-dependent tRNA methyltransferase), was detected in both BY4742 and *tcs2*∆ and is illustrated in Figure S6A and B. For translation of full-length Trm140, the ribosome must undergo a +1 frameshift at nucleotide 832. As seen in Figure S6A and B, nearly 100% of the reads begin in Frame 0, then after base 832, nearly 100% of the reads are in the +1 frame. 

87 and 213 ORFs were found to have potential translational ambiguities in BY4742 and *tcs2*∆, respectively (Table S6 and Table S7). GO term enrichment of these genes with translational ambiguities revealed a single biological process, cytoplasmic translation, was enriched in both strains, with 16 genes in BY4742 (*P *= 4 x 10^-6^) and 35 genes in *tsc2*∆ (*P *= 3 x10^-11^). In *tcs2*∆, 17 of the 79 ribosomal proteins had increased levels of translation ambiguities (Table S7). 

Interestingly, global analysis of translational ambiguities, by summing all reads used to determine frame, indicates that 80% of all reads from the ribosome profiling are in the correct, annotated frame (Frame 0), but there is a significant difference in translational ambiguities between wild type and mutant (*P *= 6.5 X 10^-98^, t-test), Figure S7. Interestingly, only 0.26% of all ORFs were identified as having potential translational ambiguities in *tcs2*∆. Thus, the data indicates that loss of t^6^A is causing ambiguities at discrete sequences, or codons, but is not causing a global, cataclysmic alteration of reading frame.

### The number observed non-AUG starts doubles in the t^6^A-deficient strain

*TCS2* (*SUA5*) was discovered in yeast as a suppressor of a translational initiation defect in the *cyc1-362* allele 34. *cyc1-362* contains an aberrant upstream and out of frame AUG resulting in ~2% of the normal Cyc1 protein levels. Suppressors would bypass the out of frame AUG and initiate at the correct downstream AUG, increasing the amount of Cyc1 34. To detect initiation of translation at non-conical codons, we parsed the profiling data with a strict set of parameters. To be considered a non-canonical start codon, a GUG, UUG, or GUC codons (the most frequently used non-AUG initiation codons in yeast) 74,75,76,77,78 had to be within 100 nucleotides upstream of the ORF of interest, and be in-frame with the downstream AUG with no stop codon between the candidate non-AUG and its downstream AUG. Finally, a minimum of 128 reads was required to cover the non-AUG site.

In yeast, there are two well-characterized occurrences of non-AUG initiation occurring upstream of the annotated AUG start site. *ALA1* encodes both the cytoplasmic and mitochondrial alanyl-tRNA synthetase. The cytoplasmic form of Ala1p is translated from the annotated AUG, and the mitochondrial form is translated from a pair of ACG codons located at -25 and -24 relative to AUG 79. *GRS1* encodes the cytoplasmic and mitochondrial glycyl-tRNA synthase. The cytoplasmic form of Grs1p is translated from the annotated AUG, and the mitochondrial form is translated from a UUG codon located at -26, relative to the AUG 78. In both BY4742 and *tcs2*∆, initiation at the upstream non-AUG codons can be detected for *ALA1* and *GRS1*, Figure S8A and Figure S8B.

The analysis of non-AUG initiation was expanded to the entire profiling dataset. For the three codons analysed, *tcs2*∆ contain nearly twice as many non-AUG starts as BY4742. For initiation at UUG, BY4742 contained 140 genes, Table S8, and *tcs2*∆ contained 260, Table S9. For initiation at ACG, BY4742 contained 98 genes, Table S10, and *tcs2*∆ contained 169, Table S11. For initiation at GUG, BY4742 contained 62 genes, Table S12, and *tcs2*∆ contained 134, Table S13. None of these sets of genes contained any enrichment of GO terms. 

### t^6^A’s role in translation speed varies with the codon

**Figure 6:**
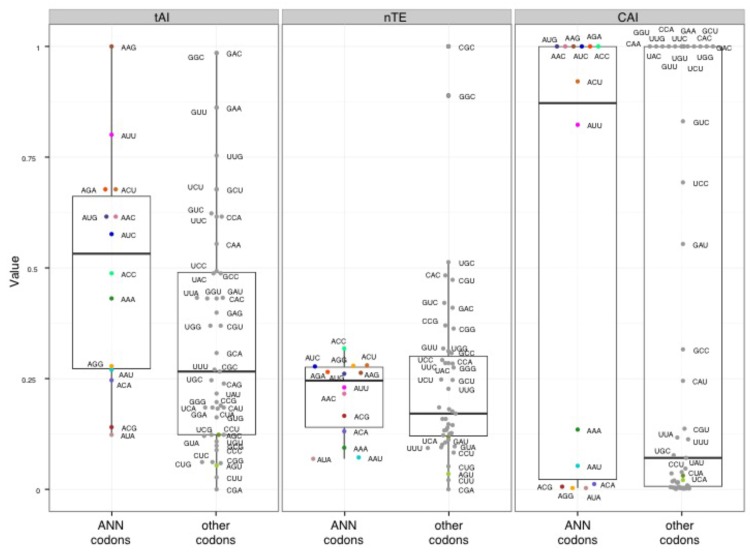
FIGURE 6: Translation efficiency of t^6^A-dependent codons. tAI – tRNA Adaptation Index; nTE – normalized Translational Efficiency; CAI – Codon Adaptation Index. Black and colored circles indicate a codon decoded by a tRNA predicted by the wobble hypothesis, with color matching Figure 1 and 7.

Different metrics have been developed to estimate translation efficiencies of individual codons based on the abundance of their cognate tRNAs, and the properties of the ASL they form. There are three major metrics commonly used to measure the translation efficiency of codons: the Codon adaptation index (CAI, 80), the tRNA adaptation index (tAI, 81), and the normalized Translational Efficiency (nTE, 82) (which is based on codon abundance in the transcriptome rather than codon frequency in the genome). Using the three metrics, we found that ANN codons in yeast have in average a higher estimated translational efficiency compared to other codons (Figure 6), suggesting that there is a statistical tendency for ANN-tRNAs to be in high supply in standard growth conditions. The predicted efficiencies vary greatly between ANN codons, with AAG always in high supply while AUA always in low supply (Figure 6). 

**Figure 7:**
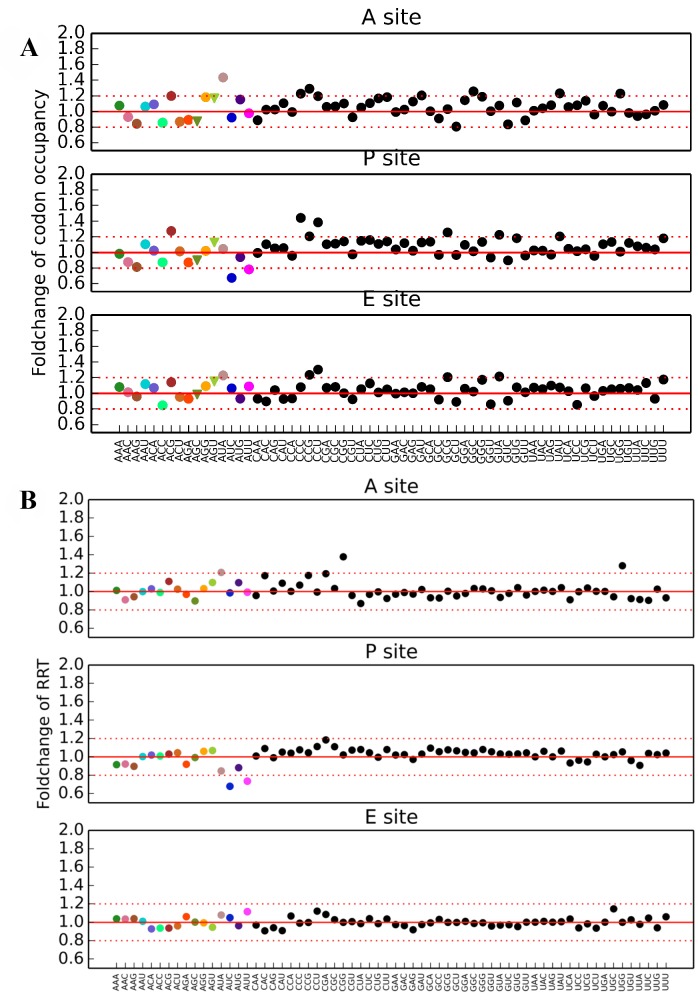
FIGURE 7: Measurements of ribosome pausing at the A-, P-, and E-sites of *tcs2*∆. **(A)** Codon occupancy. **(B)** Ribosome residence time (RRT). Black and colored circles indicate a codon decoded by a tRNA predicted by the wobble hypothesis, with color matching Figure 1 and 6.

Ribosome profiling data allows for evaluation of translation speed by measuring codon occupancy at each site in the ribosome, with increased occupancy analogous to a decrease elongation rate, and vice versa. Using two different methods, the Codon Occupancy (CO) 83 and the Ribosome residence time (RRT) 84, a count of every codon occupying the ribosomal A (acceptor), P (peptidyl transfer), and E (exit) sites was compiled. Comparing the Log_2_ fold-change of CO and RRT in the A, P, and E sites of *tcs2*∆ and BY4742 produced a global summary of the consequences of t^6^A absence on decoding, Figure 7. 

The ribosomal A site occupancy for t^6^A dependent codon site occupancy was increased for AUA, ACG, AGG, AUG, ACA, AAA, and AAU and decreased for AUU, AAC, AUC, AGA, ACU, ACC, and AAG. The codons with increased occupancy in the A-site fell into two categories: (i) codons that are decoded by rare tRNAs (only 1-4 copies of tRNAs genes are encoded in the chromosome) and (ii) codons that are decoded by a G_34_:U_3_ wobble, as for AAU (decoded by tRNA^Asn^_(GUU)_) (see Figure 1B for codon:anticodon pairs). The codons whose A-site occupancy decreased also fell into two categories: (i) codons decoded abundant by tRNAs (4-13 genes) and (ii) codons decoded by an I_34_:C_3_ wobble, as for AUC (decoded by tRNA^Ile^_(IAU)_) and ACC (decoded by tRNA^Thr^_(IGU)_) (Figure 1B). The pattern found for A-site occupancy, also held true for P-site and E-site occupancies. Interestingly, this pattern also held true for codons decoded by non t^6^A-containing tRNAs. AGU (G:U wobble) and CGG (decoded by the rare tRNA^Arg^_CCG_) were increased in ribosome occupancy, while GUC and GCU (I:C or I:U) were decreased in ribosome occupancy (Figure 7). From this data, it appears that t^6^A is helping increase elongation rate of rare tRNAs and G_34_:U_3_ pairs and decrease the elongation rate of high abundance and I_34_:C_3_ pairs to homogenize the process of elongation.

## DISCUSSION

The absence of t^6^A in yeast does not lead to catastrophic and global defects in translation, as would be expected from previous studies based on single reporter assays. Even with doubling of initiation at upstream non-AUG starts and a 2.5 fold increase in translational ambiguities, only a limited number of genes in the yeast genome were affected. This suggests that the severe and pleiotropic phenotypes caused by t^6^A deficiency may not be caused by global defects in translation, but instead of the subtler consequences of codon-specific translation defects caused by lack of t^6^A. 

### Role of t^6^A in decoding efficiency varies with the tRNA

The codon occupancy results presented in this study suggest that t^6^A helps rare cognate tRNAs and G:U mismatches (near-cognates) compete with Watson:Crick decoding tRNAs and slows decoding by high abundance tRNAs and tRNAs using the wobble U:C base pairings 85. This can be all the more critical for codons like AGG decoded by tRNA^Arg^_CCU _that are known to be strongly inhibitory for translation efficiency 86,87,88. Another important role for t^6^A is stabilizing the interaction between the first base of the mRNA codon and position 36 (the third nucleoside) of the tRNA anticodon, preventing decoding of near-cognates by tRNA^iMet^_CAU_ 89. This is exemplified by tRNA^fMet^ of *E. coli* that contains an unmodified A_37_ and can efficiently decode GUG and UUG while the eukaryotic tRNA^iMet^ contains t^6^A_37_ and rarely decodes non-AUG codons 90,91,92,93. The examination of alternative start-sites presented here supports the role of t^6^A preventing tRNA^iMet^ from recognizing near-cognates and restricting translation initiation to AUG codons. 

The codon occupancy for non-t^6^A containing tRNAs is also altered (Figure 7), and this is more dramatic than what is seen in *ncs2*∆*elp6*∆ 60. This could be due to an alteration in competition between cognate and near-cognate tRNAs. This was previously demonstrated for tRNA^Arg^_UCU_ (tRNA^Arg^_III_), which naturally exists in t^6^A modified and unmodified forms 94. The modified version of tRNA^Arg^_UCU_ can outcompete the unmodified form for the cognate codon and binds more tightly to tRNA^Ser^_GGA_ involving a U_36_:G_34_ mismatch 94. CGG codons (decoded by tRNA^Arg^_CCG_) and UGG codons (tRNA^Trp^_CCA_) are increased in both the codon occupancy and RRT assays (Figure 7). One can speculate that the slower decoding at CGG and UGG is due to competition between tRNA^Arg^_CCG_ or tRNA^Trp^_CCA_ with an unmodified near-cognate tRNA^Arg^_CCU_. 

A recent global analysis of yeast ribosome profiling data has shown that frequent codons are decoded more quickly than rare codons, and AT-rich codons are decoded more quickly than GC-rich codons 84. It seems that the difference could be even larger if tRNA modifications are altered, as shown here with the absence of t^6^A, and as is already know for several other ASL modifications. The absence of Queuosine (Q_34_) is known to have opposite effects on decoding depending on identity of the 3^rd^ base of the codon 88,95, and the depletion of mcm^5^s^2^U synthesis can alter the decoding rates of tRNAs that do not possess this modification 60. An emerging general trend for the ASL modifications is to homogenize the kinetics of individual tRNA binding (competition) during translation and to alter the speed of translation to ensure proper protein folding, a concept that was predicted by Toshimichi Ikemura in 1981 96. 

### Could defects in translation speed cause the pleiotropic phenotypes of t^6^A^-^? 

Analysis of codon stretches in yeast 20 revealed that the genes with the longest stretches of t^6^A-dependent codons encode poly-Asn (poly-N) proteins that contain up to 31 consecutive AAU/AAC codons. These include *GPR1, *required for glucose activation of the cAMP pathway 97, and *SWI1*, a subunit of the SWI/SNF chromatin remodelling complex required for transcription of many genes involved in sugar catabolism, as well as meiosis cell mating type (see summary in SGD 98). If the protein expression of these two genes is reduced in the absence of t^6^A (that is a decrease in elongation speed of these transcripts possibly due to stalling), many of the phenotypes seen in t^6^A^-^ strains (e.g., no growth on galactose, chromatin remodelling defects and telomere shortening 33,99,100) can be explained. Unfortunately, the presence of these repeats (90 nts) are longer than the RPFs (28 nts) sequenced, so these genes could not be analysed in the ribosome profiling and further studies are needed to test this hypothesis.

Several stress-induced transcription factors are also increased in *tcs2*∆ context, including NSF1 (YPL230W), a transcriptional regulator of genes involved in growth on non-fermentable carbon sources (see summary in SGD 98), Sol4 (YGR248W), which functions in the pentose phosphate pathway (see summary in SGD 98), and Smc6 (YLR383W), a component of the SMC5-SMC6 complex that plays a key role in the removal of X-shaped DNA structures (see summary in SGD 98) (Table S4). Up-regulation of any of these transcription factors would have wide-ranging effects and could explain some of the pleiotropic phenotypes seen under t^6^A deficiency. 

Ribosome profiling of *ncs2*∆ revealed genes with increased translation activity tend to play a role in amino acid metabolism, and *GCN4* is significantly increased 54. Comparison of genes increased in *tcs2*∆ and *ncs6*∆ revealed 19 genes found in each of these mutants, Table S14. Increased genes included *GCN4* and several members of the arginine biosynthesis pathway, Table S14. Twelve genes are decreased in both *tcs2*∆ and *ncs6*∆, including two ribosomal protein subunits and two phosphatases, Table S15. *GCN4* is also increased in the ribosome profiling of *ncs2*∆*elp6*∆ 60. A common theme seen in disrupting ASL modifications is the de-repression of Gcn4, in a non-canonical Gcn2-independent manner, activating a small subset of Gcn4 regulated genes. The mechanism of activation and the importance of Gcn4 activation are not understood at this point in time.

### Restoring protein homeostasis suppresses the slow growth of *tcs2*∆

An explanation for the similarity of phenotypes seen in both Elongator and t^6^A mutants could be due to the disruption of protein homeostasis. Unlike the mutations in the Elongator modification, the slow growth observed during disruption of t^6^A biosynthesis cannot be suppressed by overexpression of tRNAs (Figure 3). However, L-homoserine rescued the growth of *tcs2*∆, but not *tcs3*∆ (Figure 4). Further studies are required to explain this suppression, but homoserine is a toxic intermediate, which acts as a threonine analogue, and it was recently shown that the ubiquitin pathway and the proteasome are crucial in alleviating homoserine toxicity 65. 

### Model for the cellular response to reduction of t^6^A

**Figure 8:**
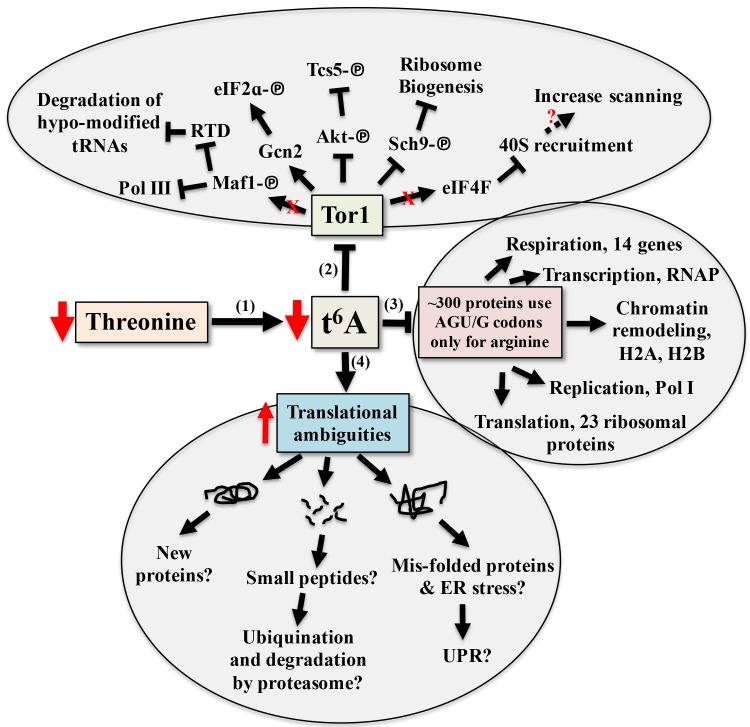
FIGURE 8: Model for the cellular response to reduction of t^6^A. **(1) **Reduction of threonine lowers the level of t^6^A and **(2) **decreases the activity of the master controller Tor, which reduces anabolism and the growth potential of the cell through multiple pathways. **(3)** ~300 proteins only use t^6^A encoding tRNAs for arginine. **(4)** Potential outcomes of increased translation ambiguities seen in the absence of t^6^A.

To date, *tcs2* and members of the TCTC complex have been implicated in transcriptional regulation. While there is no empirical evidence eliminating this possibly, the evidence presented here and in other works suggests transcriptional changes seen when perturbing t^6^A biosynthetic genes are part of an adaptive response by the cell to cope with translational errors. The response to alterations in t^6^A levels involves a combination of both independent and interrelated events, summarized in Figure 8. The model proposes that t^6^A acts as a sensor of nutritional levels as t^6^A varies in response to the availability of threonine, Figure 8-1 101. As t^6^A levels decline, Tor1 activity decreases, Figure 8-2 56. As a master controller, declines in Tor1 activity reduces the growth potential of the cell 55,56,102 and has wide ranging affects, from blocking Pol I, Pol III 103, and the RTD (Rapid tRNA Degradation pathway to prevent degradation of hypo-modified tRNAs) 104 to lessening translation initiation and ribosome biogenesis 105.

A reduction in t^6^A may also lower the translation rate of specific proteins due to codon usage, Figure 8-3. Arginine is one of two amino acids in yeast that are incorporated both by t^6^A-containing tRNAs (AGA/G codons) and by tRNAs lacking t^6^A (CGN codons), Figure 1B 44. The AGA/G codons are known to be frequent sites of frameshifting, reviewed in 106. Using the codon usage database 107, yeast genes were ranked according to their use of t^6^A-dependent or t^6^A-independent Arg codons. Around 300 genes used only t^6^A-dependent Arg codons and GO analysis showed a strong enrichment for genes of the aerobic respiration and electron transport pathways (*P *- value 10^-9^, Holm-Bonferroni test), which could explain the respiratory deficiency phenotype displayed by t^6^A^-^ yeast strains (Table S16 and S17). 23 ribosomal proteins, RNA Polymerase subunits (RNAP), and proteins of the chromatin remodelling complexes H2A and H2B use t^6^A-dependent Arg codons. Only 12 of these t^6^A-dependent Arg genes were decreased in RPFs in *tcs2*∆. Proteomic studies are now underway to confirm if this specific set of genes are translated less efficiently in the t^6^A^- ^strains.

As t^6^A levels decrease, translation fidelity decreases (Figures S7, S8 and Tables S6-13). Increase in translation ambiguity could lead to new protein products, which may be non-functional or toxic 108. Out-of-frame decoding could increase synthesis of small peptides 108 and misfolded proteins could lead to the activation of the unfolded protein response (UPR) 55, and to the activation of catabolic pathways 109,110, Figure 8-4. Additional proteomic studies are underway to measure misfolding and amino acid misincorporation rates in t^6^A^- ^strains to further characterize these multi-layered and complex phenotypes.

## MATERIALS AND METHODS

### Strains and growth conditions

A list of all organisms used in this study can be found in Table S1. Yeast strains were grown on YPD (DIFCO Laboratories) at 30°C. Synthetic minimal media (SD), with or without agar, with or without dropout supplements (-uracil, -ura; -leucine, -leu; -histidine, -his) were purchased from Clontech (Palo Alto, CA) and prepared as recommended by the manufacturer. Glucose (Glu, 2% w/v), Glycerol (Gly, 4% w/v), Ethanol (EtOH, 6% v/v) 5-fluoro-orotic acid (5-FOA, 0.1% w/v) and G418 (300 μg/mL) were used when appropriate. Yeast transformations were carried out using frozen competent cells as described by 111 with plating onto the appropriate media. The *S. cerevisiae* *tcs2*∆::*KanMX4* strain, VDC9100, was created as previously described 27. All strains were genotyped using oligonucleotides targeting inside and outside the gene of interest, in addition to the location of the replacement cassette. Oligonucleotides are listed in Table S2. VDC9100 (*tcs2*∆) harboring tRNA over-expression plasmids were created using the plasmid shuffle technique by first transforming with pBN204 (*TCS2* complementation plasmid), then transforming with tRNA plasmids, and finally curing pBN204 from VDC9100 using SD-leu+5-FOA media.

### Yeast growth assays 

Growth curves were performed using a Bioscreen C MBR (Oy Growth Curves AB Ltd, Finland) at 30°C and at maximum shaking. A 250 µl culture was used in each well, and 5 biological replicates were used for each condition. Yeast cultures were grown in the listed media to saturation, normalized to an OD_600_ of 1, and diluted 200 times in the listed media before loading on the Bioscreen. The growth curves presented are averages of 5 biological replicates. Significance was determined using a 2-way ANOVA and Fisher’s LSD using Prism 6 (GraphPad). 

For phenotype screens and tRNA over-expression assays, yeast cultures were grown in the media listed in the figure to saturation, normalized to an OD_600_ of 1.0 and 5 µL of 1:10 serial dilutions were spotted on the listed media with the supplements listed in the figure and text. Galactose (2% w/v) was added when needed. 

### Extraction and digestion of bulk tRNAs 

Bulk tRNAs were prepared as previously described using acid buffered-phenol (phenol saturated with 50 mM sodium acetate, pH 5.8) and alcohol precipitation 20. Nucleosides were prepared as described in 61 by hydrolyzing bulk tRNA with 10 units of Nuclease P1 (Sigma) overnight at 37°C, with the addition of 0.01 units of phosphodiesterase I (Sigma) and 3 µL *E. coli* alkaline phosphatase (Sigma). The hydrolyzed nucleosides were further purified by filtering through a 5 kD MWCO filter (Millipore) (to remove enzymes), dried in a CentriVap Concentrator, and suspended in 20 µL of water prior to analysis by HPLC or LC-MS/MS. 

### HPLC and LC-MS/MS Analysis

t^6^A was detected by HPLC as described by 112 using a Waters 1525 HPLC with Empower 2 software and detected with a Waters 2487 UV-vis spectrophotometer with simultaneous detection at 254 nm and 313 nm (for thio-derivatives). Separation was performed on an Ace C-18 column heated to 30°C, using 250 mM ammonium acetate (Buffer A) and 40% acetonitrile (Buffer B) run at 1 mL/min. 100 µg of nucleosides were injected and separated using a complex step gradient 112. Levels of t^6^A were measured by integrating the peak area from the extraction ion chromatograms. The ratio of Ψ-modified base/m_2_^2^G was used to normalize for tRNA concentration across samples. Levels for mutant strains were expressed relative to wild type levels. Results were confirmed by LC–MS/MS at the Donald Danforth Plant Science Center, St. Louis MO, as described in 20. The MS/MS fragmentation data, as well as a t^6^A standard provided by D. Davis (University of Utah) were also used to confirm the presence of t^6^A.

### Ribosome profiling

#### Purification of RPFs and Library Preparation

Ribosome Profiling was performed as described previously by Baudin-Baillieu *et al. *113,114. Briefly, polysomes were prepared from two biological replicates of the parental BY4742 strain and *tcs2*∆ (VDC9100) grown from a preculture diluted into 500 mL YPD in an Erlenmeyer flask. Cells were harvested at OD_600_ 0.6, chilled on ice, and cycloheximide was added to a final concentration of 50 µg/mL. Polysomes were harvested in cold lysis buffer (0.1 mM Tris-HCl, pH 7.4, 10 mM NaCl_2_, 3 mM MgCl_2_, and 50 µg/mL cycloheximide), and were aliquoted at approximately 40-50 OD_260_ units per tube and rapidly frozen in liquid nitrogen and stored at -80°C. Monosomes were prepared by digesting polysome extracts for 1 hour at room temperature with 15 units RNaseI (Ambion) per OD unit. The digested polysomes were purified on sucrose gradients prepared by casting the sucrose gradients (31% sucrose, 50 mM Tris-acetate pH 7.6, 50 mM NH_4_Cl, 12 mM MgCl_2_, and 1 mM DTT) with three freeze-thaw cycles. The samples were loaded on the gradients and centrifuged in a Beckman SW41 rotor at 39,000 rpm at 4°C for 3 hours. Fractions were collected using an ISCO (Teledyne, Lincoln, NE) instrument at a 0.5 mL/min flow rate. Ribosome protected fragments (RPFs) of mRNA were purified using acid phenol (unbuffered), chloroform, and ethanol precipitation, then stored at -20°C. The 28-nucleotide RNA fragments were selected on 15% acrylamide gels containing 7 M urea. A 28 nt RNA oligonucleotide (oNTI 199 5'-AUGUACACGGAGUCGACCCGCAACGCGA-3') was used as a size marker. After migration, gels were incubated for 30 minutes in a 10 % solution of SYBR-Gold (Life Technologies) and visualized with a UV lamp at 300 nm, and the 28 nt fragments were excised. The excised gel pieces were loaded into the pierced 1.5 mL tubes inside a 2 mL tube, and centrifuged for 1 minute at 16,000 x g. RNA was precipitated with glycogen ethanol overnight at -20°C. RPFs were depleted of major rRNA contamination by subtractive hybridization using biotinylated oligonucleotides (Table S2) and were recovered by reacting with MageneShere Paramagnetic streptavidin particles (Promega). The supernatants containing the RPFs were recovered and the RNA was precipitated, as described above. RNA size and quality was checked with a Small RNA Chip on a Bioanalyzer 2100 (Agilent). A directional RNA-Seq library was prepared by IMAGIF (Centre de Recherche de Gif - www.imagif.cnrs.fr) Gif-sur-Yvette, France using the TruSeq Small RNA Sample Prep Kit (Illumina) and the v1.5 sRNA 3' Adaptor (Illumina) according to the manufacturer’s protocol and verified using Bioanalyzer Small RNA Analysis kit (Agilent). Sequencing was performed at the Microarray and Genomic Analysis Core Facility at the University of Utah Huntsman Cancer Institute on an Illumina HiSeq 1500 and subjected to a 50 cycle run.

#### Sequencing, quality control, and read-mapping

Sequencing and bioinformatics analysis were performed as described in 114. In brief, four sequencing libraries were prepared from the 28-mer RPFs purified from two biological replicates of BY4742 and *tcs2*∆. The libraries were sequenced on an Illumina HiSeq 1500 with 50-cycle single-end reads to maximize the numbers of reads for each small RNA library. Approximately 2 x 10^9^ reads were obtained for each sample. Quality was assessed using FastQC (http://www.bioinformatics.babraham.ac.uk/projects/fastqc) and adaptors were removed using CutAdapt 115. To remove contaminating reads corresponding to rRNA, reads were mapped against an rRNA index from the *Saccharomyces* Genome Database (SGD) using Bowtie2 with the default settings 116. Reads not mapping to rRNA were mapped against the SacCer3 index (SGD) 117 using Bowtie2. Approximately 1.3 x 10^8^ reads from each sample were mapped to the *S. cerevisiae* genome. Differential expression between samples was determined using a DESeq, with multiple testing correction using Benjamini and Hochberg 118,119,120. Significant differences between wild type and mutant were declared based on an adjusted alpha of 0.05. Precision of the biological replicates was very high with R = 0.9881 and R = 0.9959 for BY4742 and *tcs2*∆, respectively (Figure S1A and B). Post-sequencing analysis to identify differential expression, frameshifts, read-through, and non-AUG starts was performed as described in 73. Sequences were deposited at NCBI GEO database under accession number GSE72030. 

#### Functional Classification of Genes 

Lists of genes produced from the above analysis were analysed using YeastMine 69, an interactive database for querying the *Saccharomyces* Genome Database (SGD,www.yeastgenome.org) 69 to produce Gene Ontology enrichments and pathway enrichments.

### Detection of protein aggregates and AGEs

Proteins were extracted from cells grown to mid-log and total proteins were extracted as described in 121. Aggregates were isolated as described by 122. Total proteins and aggregates were separated on 4-20% denaturing polyacrylamide gels with Coomasie blue staining. AGEs were identified by diol-specific silver staining 67.

## SUPPLEMENTAL MATERIAL

Click here for supplemental data file.

All supplemental data for this article are also available online at http://microbialcell.com/researcharticles/global-translational-impacts-of-the-loss-of-the-trna-modification-t6a-in-yeast/.

## References

[1] Crick FHC (1966). Codon—anticodon pairing: The wobble hypothesis.. J Mol Biol.

[2] El Yacoubi B, Bailly M, de Crécy-Lagard V (2011). Biosynthesis and Function of Posttranscriptional Modifications of
Transfer RNAs.. Annu Rev Genet.

[3] Grosjean H, de Crécy-Lagard V, Marck C (2010). Deciphering synonymous codons in the three domains of life:
co-evolution with specific tRNA modification enzymes.. FEBS Lett.

[4] Novoa EM, Pavon-Eternod M, Pan T, Ribas de Pouplana L (2012). A role for tRNA modifications in genome structure and codon
usage.. Cell.

[5] Lamichhane TN, Blewett NH, Crawford AK, Cherkasova V a, Iben JR, Begley TJ, Farabaugh PJ, Maraia RJ (2013). Lack of tRNA modification isopentenyl-A37 alters mRNA decoding
and causes metabolic deficiencies in fission yeast.. Mol Cell Biol.

[6] Novoa EM, Ribas de Pouplana L (2012). Speeding with control: codon usage, tRNAs, and
ribosomes..

[7] Ran W, Higgs PG (2010). The influence of anticodon-codon interactions and modified bases
on codon usage bias in bacteria.. Mol Biol Evol.

[8] Ran W, Kristensen DM, Koonin E V (2014). Coupling between protein level selection and codon usage
optimization in the evolution of bacteria and archaea.. MBio.

[9] Ran W, Higgs PG (2012). Contributions of Speed and Accuracy to Translational Selection in
Bacteria.. PLoS One.

[10] Plotkin JB, Kudla G (2011). Synonymous but not the same: the causes and consequences of codon
bias.. Nat Rev Genet.

[11] KrisKo A, Copic T, Gabaldón T, Lehner B, Supek F (2014). Inferring gene function from evolutionary change in signatures of
translation efficiency.. Genome Biol.

[12] Wallace EWJ, Airoldi EM, Drummond DA (2013). Estimating selection on synonymous codon usage from noisy
experimental data.. Mol Biol Evol.

[13] Drummond DA, Wilke CO (2009). The evolutionary consequences of erroneous protein
synthesis.. Nat Rev Genet.

[14] Drummond DA, Bloom JD, Adami C, Wilke CO, Arnold FH (2005). Why highly expressed proteins evolve slowly.. Proc Natl Acad Sci U S A.

[15] Dedon PC, Begley TJ (2014). A system of RNA modifications and biased codon use controls
cellular stress response at the level of translation.. Chem Res Toxicol.

[16] Preston M a, D’Silva S, Kon Y, Phizicky EM (2013). tRNAHis 5-methylcytidine levels increase in response to several
growth arrest conditions in Saccharomyces cerevisiae.. RNA.

[17] Chan CTY, Dyavaiah M, DeMott MS, Taghizadeh K, Dedon PC, Begley TJ (2010). A quantitative systems approach reveals dynamic control of tRNA
modifications during cellular stress.. PLoS Genet.

[18] Gu C, Begley TJ, Dedon PC (2014). tRNA modifications regulate translation during cellular
stress.. FEBS Lett.

[19] Thiaville PC, Iwata-Reuyl D, de Crécy-Lagard V (2014). Diversity of the biosynthesis pathway for
threonylcarbamoyladenosine (t(6)A), a universal modification of
tRNA.. RNA Biol.

[20] El Yacoubi B, Lyons B, Cruz Y, Reddy R, Nordin B, Agnelli F, Williamson JR, Schimmel P, Swairjo MA, de Crécy-Lagard V (2009). The universal YrdC/Sua5 family is required for the formation of
threonylcarbamoyladenosine in tRNA.. Nucleic Acids Res.

[21] Deutsch C, El Yacoubi B, de Crécy-Lagard V, Iwata-Reuyl D (2012). Biosynthesis of threonylcarbamoyl adenosine (t6A), a universal
tRNA nucleoside.. J Biol Chem.

[22] Lauhon CT (2012). Mechanism of N6-Threonylcarbamoyladenonsine (t6A) Biosynthesis:
Isolation and Characterization of the Intermediate
Threonylcarbamoyl-AMP.. Biochemistry.

[23] El Yacoubi B, Hatin I, Deutsch C, Kahveci T, Rousset J-P, Iwata-Reuyl D, Murzin AG, de Crécy-Lagard V (2011). A role for the universal Kae1/Qri7/YgjD (COG0533) family in tRNA
modification.. EMBO J.

[24] Perrochia L, Guetta D, Hecker A, Forterre P, Basta T (2013). Functional assignment of KEOPS/EKC complex subunits in the
biosynthesis of the universal t6A tRNA modification.. Nucleic Acids Res.

[25] Perrochia L, Crozat E, Hecker A, Zhang W, Bareille J, Collinet B, van Tilbeurgh H, Forterre P, Basta T (2013). In vitro biosynthesis of a universal t6A tRNA modification in
Archaea and Eukarya.. Nucleic Acids Res.

[26] Wan LCK, Mao DYL, Neculai D, Strecker J, Chiovitti D, Kurinov I, Poda G, Thevakumaran N, Yuan F, Szilard RK, Lissina E, Nislow C, Caudy A a, Durocher D, Sicheri F (2013). Reconstitution and characterization of eukaryotic
N6-threonylcarbamoylation of tRNA using a minimal enzyme
system.. Nucleic Acids Res.

[27] Thiaville PC, El Yacoubi B, Perrochia L, Hecker A, Prigent M, Thiaville JJ, Forterre P, Namy O, Basta T, de Crécy-Lagard V (2014). Cross Kingdom Functional Conservation of the Core Universally
Conserved Threonylcarbamoyladenosine tRNA Synthesis Enzymes.. Eukaryot Cell.

[28] Meng F-L, Hu Y, Shen N, Tong X-J, Wang J, Ding J, Zhou J-Q (2009). Sua5p a single-stranded telomeric DNA-binding protein facilitates
telomere replication.. EMBO J.

[29] Downey M, Houlsworth R, Maringele L, Rollie A, Brehme M, Galicia S, Guillard S, Partington M, Zubko MK, Krogan NJ, Emili A, Greenblatt JF, Harrington L, Lydall D, Durocher D (2006). A genome-wide screen identifies the evolutionarily conserved
KEOPS complex as a telomere regulator.. Cell.

[30] Kisseleva-Romanova E, Lopreiato R, Baudin-Baillieu A, Rousselle J-C, Ilan L, Hofmann K, Namane A, Mann C, Libri D (2006). Yeast homolog of a cancer-testis antigen defines a new
transcription complex.. EMBO J.

[31] Hecker A, Graille M, Madec E, Gadelle D, Le Cam E, van Tilbergh H, Forterre P (2009). The universal Kae1 protein and the associated Bud32 kinase
(PRPK), a mysterious protein couple probably essential for genome
maintenance in Archaea and Eukarya.. Biochem Soc Trans.

[32] Oberto J, Breuil N, Hecker A, Farina F, Brochier-Armanet C, Culetto E, Forterre P (2009). Qri7/OSGEPL, the mitochondrial version of the universal Kae1/YgjD
protein, is essential for mitochondrial genome maintenance.. Nucleic Acids Res.

[33] Lin C a, Ellis SR, True HL (2010). The Sua5 protein is essential for normal translational regulation
in yeast.. Mol Cell Biol.

[34] Hampsey M, Na JG, Pinto I, Ware DE, Berroteran RW (1991). Extragenic suppressors of a translation initiation defect in the
cyc1 gene of Saccharomyces cerevisiae.. Biochimie.

[35] Natarajan K, Meyer MR, Jackson BM, Slade D, Roberts C, Hinnebusch AG, Marton MJ (2001). Transcriptional profiling shows that Gcn4p is a master regulator
of gene expression during amino acid starvation in yeast.. Mol Cell Biol.

[36] Foiani M, Cigan AM, Paddon CJ, Harashima S, Hinnebusch AG (1991). GCD2, a translational repressor of the GCN4 gene, has a general
function in the initiation of protein synthesis in Saccharomyces
cerevisiae.. Mol Cell Biol.

[37] Hinnebusch AG (2005). Translational regulation of GCN4 and the general amino acid
control of yeast.. Annu Rev Microbiol.

[38] Daugeron M-C, Lenstra TL, Frizzarin M, El Yacoubi B, Liu X, Baudin-Baillieu A, Lijnzaad P, Decourty L, Saveanu C, Jacquier A, Holstege FCP, de Crécy-Lagard V, van Tilbeurgh H, Libri D (2011). Gcn4 misregulation reveals a direct role for the evolutionary
conserved EKC/KEOPS in the t6A modification of tRNAs.. Nucleic Acids Res.

[39] Guy MP, Phizicky EM (2015). Conservation of an intricate circuit for crucial modifications of
the tRNAPhe anticodon loop in eukaryotes.. RNA.

[40] Guy MP, Podyma BM, Preston MA, Shaheen HH, Krivos KL, Limbach PA, Hopper AK, Phizicky EM (2012). Yeast Trm7 interacts with distinct proteins for critical
modifications of the tRNAPhe anticodon loop.. RNA.

[41] Noma A, Kirino Y, Ikeuchi Y, Suzuki T (2006). Biosynthesis of wybutosine, a hyper-modified nucleoside in
eukaryotic phenylalanine tRNA.. EMBO J.

[42] Thiaville PC, El Yacoubi B, Köhrer C, Thiaville JJ, Deutsch C, Iwata-Reuyl D, Bacusmo JM, Armengaud J, Bessho Y, Wetzel C, Cao X, Limbach PA, RajBhandary UL, de Crécy-Lagard V (2015). Essentiality of threonylcarbamoyladenosine (t(6)A), a universal
tRNA modification, in bacteria.. Mol Microbiol.

[43] Taniguchi T, Miyauchi K, Nakane D, Miyata M, Muto A, Nishimura S, Suzuki T (2013). Decoding system for the AUA codon by tRNAIle with the UAU
anticodon in Mycoplasma mobile.. Nucleic Acids Res.

[44] Machnicka M a, Milanowska K, Osman Oglou O, Purta E, Kurkowska M, Olchowik A, Januszewski W, Kalinowski S, Dunin-Horkawicz S, Rother KM, Helm M, Bujnicki JM, Grosjean H (2013). MODOMICS: a database of RNA modification pathways--2013
update.. Nucleic Acids Res.

[45] Björk GR, Huang B, Persson OP, Byström AS (2007). A conserved modified wobble nucleoside (mcm5s2U) in lysyl-tRNA is
required for viability in yeast.. RNA.

[46] Dewez M, Bauer F, Dieu M, Raes M, Vandenhaute J, Hermand D (2008). The conserved Wobble uridine tRNA thiolase Ctu1-Ctu2 is required
to maintain genome integrity.. Proc Natl Acad Sci U S A.

[47] Johansson MJO, Esberg A, Huang B, Björk GR, Byström AS (2008). Eukaryotic wobble uridine modifications promote a functionally
redundant decoding system.. Mol Cell Biol.

[48] Jäger G, Nilsson K, Björk GR (2013). The phenotype of many independently isolated +1 frameshift
suppressor mutants supports a pivotal role of the P-site in reading frame
maintenance.. PLoS One.

[49] Urbonavicius J, Stahl G, Durand JMB, Ben Salem SN, Qian Q, Farabaugh PJ, Björk GR (2003). Transfer RNA modifications that alter +1 frameshifting in general
fail to affect -1 frameshifting.. RNA.

[50] Huang B, Johansson MJO, Byström AS (2005). An early step in wobble uridine tRNA modification requires the
Elongator complex.. RNA.

[51] Chen C, Huang B, Eliasson M, Rydén P, Byström AS (2011). Elongator Complex Influences Telomeric Gene Silencing and DNA
Damage Response by Its Role in Wobble Uridine tRNA
Modification.. PLoS Genet.

[52] Bauer F, Hermand D (2012). A coordinated codon-dependent regulation of translation by
Elongator.. Cell Cycle.

[53] Esberg A, Huang B, Johansson MJO, Byström AS (2006). Elevated levels of two tRNA species bypass the requirement for
elongator complex in transcription and exocytosis.. Mol Cell.

[54] Zinshteyn B, Gilbert W V (2013). Loss of a Conserved tRNA Anticodon Modification Perturbs Cellular
Signaling.. PLoS Genet.

[55] Rojas-Benítez D, Ibar C, Glavic Á (2013). The Drosophila EKC/KEOPS complex: roles in protein synthesis
homeostasis and animal growth.. Fly (Austin).

[56] Rojas-Benitez D, Thiaville PC, de Crécy-Lagard V, Glavic A (2015). The Levels of a Universally Conserved tRNA Modification Regulate
Cell Growth.. J Biol Chem.

[57] Ibar C, Cataldo VF, Vásquez-Doorman C, Olguín P, Glavic A (2013). Drosophila p53-related protein kinase is required for PI3K/TOR
pathway-dependent growth.. Development.

[58] Scheidt V, Juedes A, Baer C, Klassen R, Schaffrath R (2014). Loss of wobble uridine modification in tRNA anticodons interferes
with TOR pathway signaling.. Microb Cell.

[59] Thiaville PC, de Crecy-Lagard V (2015). The emerging role of complex modifications of tRNALysUUU in
signaling pathways.. Microb Cell.

[60] Nedialkova DD, Leidel SA (2015). Optimization of Codon Translation Rates via tRNA Modifications
Maintains Proteome Integrity.. Cell.

[61] Naor A, Thiaville PC, Altman-Price N, Cohen-Or I, Allers T, de Crécy-Lagard V, Gophna U (2012). A Genetic Investigation of the KEOPS Complex in Halophilic
Archaea.. PLoS One.

[62] Kuranda K, Leberre V, Sokol S, Palamarczyk G, François J (2006). Investigating the caffeine effects in the yeast Saccharomyces
cerevisiae brings new insights into the connection between TOR, PKC and
Ras/cAMP signalling pathways.. Mol Microbiol.

[63] Alings F, Sarin LP, Fufezan C, Drexler HC a, Leidel S a (2014). An evolutionary approach uncovers a diverse response of tRNA
2-thiolation to elevated temperatures in yeast..

[64] Klassen R, Grunewald P, Thüring KL, Eichler C, Helm M, Schaffrath R (2015). Loss of Anticodon Wobble Uridine Modifications Affects tRNALys
Function and Protein Levels in Saccharomyces cerevisiae.. PLoS One.

[65] Kingsbury JM, McCusker JH (2010). Homoserine toxicity in Saccharomyces cerevisiae and Candida
albicans homoserine kinase (thr1Delta) mutants.. Eukaryot Cell.

[66] Katz C, Cohen-Or I, Gophna U, Ron EZ (2010). The ubiquitous conserved glycopeptidase Gcp prevents accumulation
of toxic glycated proteins.. MBio.

[67] Tsai CM, Frasch CE (1982). A sensitive silver stain for detecting lipopolysaccharides in
polyacrylamide gels.. Anal Biochem.

[68] Anderson J, Phan L, Cuesta R, Carlson BA, Pak M, Asano K, Björk GR, Tamame M, Hinnebusch AG (1998). The essential Gcd10p-Gcd14p nuclear complex is required for
1-methyladenosine modification and maturation of initiator
methionyl-tRNA.. Genes Dev.

[69] Balakrishnan R, Park J, Karra K, Hitz BC, Binkley G, Hong EL, Sullivan J, Micklem G, Cherry JM (2012). YeastMine--an integrated data warehouse for Saccharomyces
cerevisiae data as a multipurpose tool-kit.. Database J Biol Databases Curation.

[70] Nishimura A, Kotani T, Sasano Y, Takagi H (2010). An antioxidative mechanism mediated by the yeast
N-acetyltransferase Mpr1: Oxidative stress-induced arginine synthesis and
its physiological role.. FEMS Yeast Res.

[71] Harbison CT, Gordon DB, Lee TI, Rinaldi NJ, Macisaac KD, Danford TW, Hannett NM, Tagne J, Reynolds DB, Yoo J, Jennings EG, Zeitlinger J, Pokholok DK, Kellis M, Rolfe PA, Takusagawa KT, Lander ES, Gifford DK, Fraenkel E, Young RA (2004). Transcriptional regulatory code of a eukaryotic
genome.. Nature.

[72] Wolin SL, Walter P (1988). Ribosome pausing and stacking during translation of a eukaryotic
mRNA.. EMBO J.

[73] Legendre R, Baudin-Baillieu A, Hatin I, Namy O (2015). RiboTools: A Galaxy toolbox for qualitative ribosome profiling
analysis.. Bioinformatics.

[74] Pearson CE (2011). Repeat associated non-ATG translation initiation: one DNA, two
transcripts, seven reading frames, potentially nine toxic
entities!. PLoS Genet.

[75] Chen S-J, Lin G, Chang K-J, Yeh L-S, Wang C-C (2008). Translational efficiency of a non-AUG initiation codon is
significantly affected by its sequence context in yeast.. J Biol Chem.

[76] Clements JM, Laz TM, Sherman F (1988). Efficiency of translation initiation by non-AUG codons in
Saccharomyces cerevisiae.. Mol Cell Biol.

[77] Chang C-P, Chen S-J, Lin C-H, Wang T-L, Wang C-C (2010). A single sequence context cannot satisfy all non-AUG initiator
codons in yeast.. BMC Microbiol.

[78] Chang K-J, Wang C-C (2004). Translation initiation from a naturally occurring non-AUG codon
in Saccharomyces cerevisiae.. J Biol Chem.

[79] Tang H-L, Yeh L-S, Chen N-K, Ripmaster T, Schimmel P, Wang C-C (2004). Translation of a yeast mitochondrial tRNA synthetase initiated at
redundant non-AUG codons.. J Biol Chem.

[80] Sharp PM, Li WH (1987). The codon Adaptation Index--a measure of directional synonymous
codon usage bias, and its potential applications.. Nucleic Acids Res.

[81] dos Reis M, Savva R, Wernisch L (2004). Solving the riddle of codon usage preferences: a test for
translational selection.. Nucleic Acids Res.

[82] Pechmann S, Frydman J (2013). Evolutionary conservation of codon optimality reveals hidden
signatures of cotranslational folding.. Nat Struct Mol Biol.

[83] Stadler M, Fire A (2011). Wobble base-pairing slows in vivo translation elongation in
metazoans.. RNA.

[84] Gardin J, Yeasmin R, Yurovsky A, Cai Y, Skiena S, Futcher B (2014). Measurement of average decoding rates of the 61 sense codons in
vivo.. Elife.

[85] Kramer EB, Farabaugh PJ (2007). The frequency of translational misreading errors in
E. coli is largely determined by tRNA competition.. RNA.

[86] Farabaugh PJ, Björk GR (1999). How translational accuracy influences reading frame
maintenance.. EMBO J.

[87] Kawakami K, Pande S, Faiola B, Moore DP, Boeke JD, Farabaugh PJ, Strathern JN, Nakamura Y, Garfinkel DJ (1993). A rare tRNA-Arg(CCU) that regulates Ty1 element ribosomal
frameshifting is essential for Ty1 retrotransposition in Saccharomyces
cerevisiae.. Genetics.

[88] Manickam N, Nag N, Abbasi A, Patel K, Farabaugh PJ (2014). Studies of translational misreading in vivo show that the
ribosome very efficiently discriminates against most potential
errors.. RNA.

[89] Dube SK, Marcker K a, Clark BF, Cory S (1968). Nucleotide sequence of N-formyl-methionyl-transfer
RNA.. Nature.

[90] Miller JH (1974). GUG and UUG are initiation codons in vivo.. Cell.

[91] Files JG, Weber K, Coulondre C, Miller JH (1975). Identification of the UUG codon as a translational initiation
codon in vivo.. J Mol Biol.

[92] Stewart JW, Sherman F, Shipman N a, Jackson M (1971). Identification and mutational relocation of the AUG codon
initiating translation of iso-1-cytochrome c in yeast.. J Biol Chem.

[93] Baralle FE, Brownlee GG (1978). AUG is the only recognisable signal sequence in the 5′ non-coding
regions of eukaryotic mRNA.. Nature.

[94] Weissenbach J, Grosjean H (1981). Effect of threonylcarbamoyl modification (t6A) in yeast tRNA Arg
III on codon-anticodon and anticodon-anticodon interactions. A thermodynamic and kinetic evaluation.. Eur J Biochem.

[95] Zaborske JM, Bauer DuMont VL, Wallace EWJ, Pan T, Aquadro CF, Drummond DA (2014). A Nutrient-Driven tRNA Modification Alters Translational Fidelity
and Genome-wide Protein Coding across an Animal Genus.. PLoS Biol.

[96] Ikemura T (1982). Correlation between the abundance of yeast transfer RNAs and the
occurrence of the respective codons in protein genes: Differences in synonymous codon choice patterns of yeast and Escherichia
coli with reference to the abundance of isoaccepting transfer RNAs.. J Mol Biol.

[97] Kraakman L, Lemaire K, Ma P, Teunlssen AWRH, Donaton MC V, Van Dijck P, Winderickx J, De Winde JH, Thevelein JM (1999). A Saccharomyces cerevisiae G-protein coupled receptor, Gpr1, is
specifically required for glucose activation of the cAMP pathway during the
transition to growth on glucose.. Mol Microbiol.

[98] Cherry JM, Hong EL, Amundsen C, Balakrishnan R, Binkley G, Chan ET, Christie KR, Costanzo MC, Dwight SS, Engel SR, Fisk DG, Hirschman JE, Hitz BC, Karra K, Krieger CJ, Miyasato SR, Nash RS, Park J, Skrzypek MS, Simison M, Weng S, Wong ED (2012). Saccharomyces Genome Database: The genomics resource of budding
yeast.. Nucleic Acids Res.

[99] Kato M, Slack FJ (2012). Ageing and the small, non-coding RNA world..

[100] Na JG, Pinto I, Hampsey M (1992). Isolation and Characterization of SUA5, a Novel Gene Required for
Normal Growth in Saccharomyces cerevisiae.. Genetics.

[101] Miller JP, Hussain Z, Schweizer MP (1976). The involvement of the anticodon adjacent modified nucleoside
N-(9-(BETA-D-ribofuranosyl) purine-6-ylcarbamoyl)-threonine in the
biological function of E.. coli tRNAile. Nucleic Acids Res.

[102] Loewith R, Hall MN (2011). Target of rapamycin (TOR) in nutrient signaling and growth
control.. Genetics.

[103] Rohde JR, Bastidas R, Puria R, Cardenas ME (2008). Nutritional control via Tor signaling in Saccharomyces
cerevisiae.. Curr Opin Microbiol.

[104] Turowski TW, Karkusiewicz I, Kowal J, Boguta M (2012). Maf1-mediated repression of RNA polymerase III transcription
inhibits tRNA degradation via RTD pathway.. RNA.

[105] Cardenas ME, Cutler NS, Lorenz MC, Di Como CJ, Heitman J (1999). The TOR signaling cascade regulates gene expression in response
to nutrients.. Genes Dev.

[106] Farabaugh PJ (1996). Programmed translational frameshifting.. Annu Rev Genet.

[107] Tumu S, Patil A, Towns W, Dyavaiah M, Begley TJ (2012). The gene-specific codon counting database: a genome-based catalog
of one-, two-, three-, four- and five-codon combinations present in
Saccharomyces cerevisiae genes.. Database (Oxford).

[108] Powers ET, Balch WE (2008). Costly mistakes: translational infidelity and protein
homeostasis.. Cell.

[109] Travers KJ, Patil CK, Wodicka L, Lockhart DJ, Weissman JS, Walter P (2000). Functional and genomic analyses reveal an essential coordination
between the unfolded protein response and ER-associated
degradation.. Cell.

[110] Herzog B, Popova B, Jakobshagen A, Shahpasandzadeh H, Braus GH (2013). Mutual cross talk between the regulators Hac1 of the unfolded
protein response and Gcn4 of the general amino acid control of Saccharomyces
cerevisiae.. Eukaryot Cell.

[111] Gietz RD, Schiestl RH (2007). Frozen competent yeast cells that can be transformed with high
efficiency using the LiAc/SS carrier DNA/PEG method.. Nat Protoc.

[112] Pomerantz SC, McCloskey JA (1990). Analysis of RNA hydrolyzates by liquid chromatography-mass
spectrometry.. Methods Enzymol.

[113] Baudin-Baillieu A, Legendre R, Kuchly C, Hatin I, Demais S, Mestdagh C, Gautheret D, Namy O (2014). Genome-wide Translational Changes Induced by the Prion
[PSI+].. Cell Rep.

[114] Baudin-Baillieu A, Hatin I, Legendre R, Namy O (2016). Translation Analysis at the Genome Scale by Ribosome
Profiling.. Methods in Molecular Biology.

[115] Martin M (2011). Cutadapt removes adapter sequences from high-throughput
sequencing reads.. EMBnet.journal.

[116] Langmead B, Salzberg SL (2012). Fast gapped-read alignment with Bowtie 2.. Nat Methods.

[117] Engel SR, Dietrich FS, Fisk DG, Binkley G, Balakrishnan R, Costanzo MC, Dwight SS, Hitz BC, Karra K, Nash RS, Weng S, Wong ED, Lloyd P, Skrzypek MS, Miyasato SR, Simison M, Cherry JM (2014). The reference genome sequence of Saccharomyces cerevisiae: then
and now.. G3 (Bethesda).

[118] Anders S, Huber W (2010). Differential expression analysis for sequence count
data.. Genome Biol.

[119] Anders S, EMBL (2012). Analysing RNA-Seq data with the DESeq package.

[120] Benjamini Y, Hochberg Y (1995). Controlling the False Discovery Rate: A Practical and Powerful
Approach to Multiple Testing.. J R Stat Soc Ser B.

[121] Kushnirov V V (2000). Rapid and reliable protein extraction from yeast.. Yeast.

[122] Koplin A, Preissler S, Llina Y, Koch M, Scior A, Erhardt M, Deuerling E (2010). A dual function for chaperones SSB-RAC and the NAC nascent
polypeptide-associated complex on ribosomes.. J Cell Biol.

