# Supplementary material for: Global translational impacts of the loss of the tRNA modification t6A in yeast
Source: Microb Cell. 2015 Dec 18;3(1):29–45. doi: 10.15698/mic2016.01.473 (PMC4717488; doi:10.15698/mic2016.01.473)
Supplement: Supplementary file 1 [file mic-03-029-s01.pdf]

Global Translational Impacts of the Loss of the tRNA Modification t^6A in Yeast

Patrick C. Thiaville[1,2,3,4], Rachel Legendre[4], Diego Rojas-Benítez[5], Agnès Baudin-Baillieu[4], Isabelle Hatin[4], Guilhem Chalancon[6], Alvaro Glavic[5], Olivier Namy[4]*, Valérie de Crécy-Lagard[1,3,]*

[1]Department of Microbiology and Cell Science, University of Florida, Gainesville, FL 32611, USA
[2]Genetics and Genomics Graduate Program, University of Florida, Gainesville, FL 32610, USA
[3]University of Florida Genetics Institute, University of Florida, Gainesville, FL 32610, USA
[4]Institut de Biologie Intégrative de la Cellule (I2BC), CEA, CNRS, Université Paris-Sud, Bâtiment 400, 91400 Orsay, France
[5]Centro de Regulación del Genoma. Facultad de Ciencias – Universidad de Chile, Santiago, Chile.
[6]Laboratory of Molecular Biology, Francis Crick Avenue, Cambridge CB2 0QH, United Kingdom

Running title: Role of t^6A synthesis

*To whom correspondence should be addressed. Olivier Namy, Institut de Biologie Intégrative de la Cellule (I2BC), CEA, CNRS, Université Paris-Sud, Bâtiment 400, 91400 Orsay, France; Email: olivier.namy@igmors.u-psud.fr, Tel: 33(0)169155051; Fax: 33(0)169157296 and Valérie de Crécy-Lagard, Department of Microbiology and Cell Science, University of Florida, P.O. Box 110700, Gainesville, FL 32611-0700; E-mail: vcrecy@ufl.edu, Tel: (352) 392 9416; Fax: (352) 392 5922

SUPPLEMENTAL

Table S1. Strains and plasmids used in this study

**Name**	**Description**	**Reference**
S. cerevisiae		
BY4741	*MATa*; *his3*Δ; *leu2*Δ; *met15*Δ; *ura3*Δ	OpenBio Systems
BY4742	*MATα*; *his3*Δ; *leu2*Δ; *met15*Δ; *ura3*Δ	OpenBio Systems
VDC9100	*tcs2*Δ. BY4742, *sua5*::kanMX4	[1]
VDC5563	*tcs3*Δ. BY4741; *kae1*::kanMX4	[2]
Y03801	*tcs4*Δ. BY4741; *qri7*::kanMX4	Euroscarf
15914	*tcs5*Δ. BY4742; *bud32*::kanMX4	OpenBio Systems
Y07104	*tcs6*Δ. BY4741; *pcc1*::KanMX4	Euroscarf
Y06725	*tcs7*Δ. BY4741; *cgi121*::KanMX4	Euroscarf
Y07017	*tcs8*Δ. BY4741; *gon7*::KanMX4	Euroscarf
2742	BY4741; *elp3*::kanMX4	OpenBio Systems
559	BY4741; *trm9*::kanMX4	OpenBio Systems
7242	BY4741; *ncs2*::kanMX4	OpenBio Systems
7770	BY4741; *ncs6*::kanMX4	OpenBio Systems
E. coli		
DH5α	F$^-$, *gyrA462*, *endA1*, *Δ(sr1-recA)*, *mcrB*, *mrr*, *hsd*S20*(rB–, mB–)*, *supE44*, *ara-14*, *galK2*, *lacY1*, *proA2*, *rpsL20(*Sm^R*)*, *xyl-5*, *λ*$^-$, *leu*, *mtl1*	Invitrogen
Plasmids		
pRS425	2μ *LEU2*	[3]
pYES-DEST52	2μ *URA3*	Invitrogen
tM(CAU)O2	pRS425 tRNA overexpression vector	Like in [4]
tK(CUU)G1	pRS425 tRNA overexpression vector	Like in [4]
tN(GUU)C	pRS425 tRNA overexpression vector	Like in [4]
tE(UUC)U	pRS425 tRNA overexpression vector	[4]
tK(UUU)L	pRS425 tRNA overexpression vector	[4]
tI(AAU)G	pRS425 tRNA overexpression vector	Like in [4]
tT(UGU)G1	pRS425 tRNA overexpression vector	Like in [4]
tR(UCU)D	pRS425 tRNA overexpression vector	Like in [4]
tR(ACG)E	pRS425 tRNA overexpression vector	Like in [4]
tR(CCU)J	pRS425 tRNA overexpression vector	Like in [4]

tI(UAU)L	pRS425 tRNA overexpression vector	Like in [4]
pBN204	pYes containing *TCS2* plus 200 bases upstream	[5]
pXbp1	2μ *URA3* P_{GAL}::*XBP1* (YIL101c)	OpenBio Systems
pKar2	2μ *URA3* P_{GAL}::*KAR2* (YJL034w)	OpenBio Systems
pHrd1	2μ *URA3* P_{GAL}::*HRD1* (YOL013c)	OpenBio Systems
pIMT100-1	YEp351 containing $tRNA^{iMet}$ (*IMT*)	[6]
pD1780	YEp24 containing eIF-2αβγ (*SUI1*, *SUI2*, *GCD11*)	[6]
YEp24	2μ *URA3*	[7]
YEp351	2μ *URA3*	[7]

Table S2. Oligonucleotides used in this study

Name	Sequence
>KanMx-5’	gcggccgcATGGGTAAGGAAAAGACTCACGTTTC
>KanMx-3’	gcggccgcTTAGAAAAACTCATCGAGCATCAAATG
>Kae1-int-fwd	GCCTCGAGACACGGCAAGGC
>Kae1-int-rev	TGTAGCCAGGCGAGGGCTCA
>Kae1-ext-fwd	TCCATTTTGAGGATTCCTATGTGCTCG
>Kae1-ext-rev	GCTTGCCTTGTTGCTTGCTCCC
>Qri7-int-fwd	GGCAGAGGCGTGCATTCAAC
>Qri7-int-rev	CAATTCCTTCTCTCGCATTG
>Qri7-ext-fwd	TGGTTGGAAGATGCAGGCGCT
>Qri7-ext-rev	AAGGCGTCGTGCCGCTCATC
>Sua5-Int-fwd	GCAGCGCTAGTTGAAGCGGC
>Sua5-int-rev	ACCTTGCACGCTCCGCCATC
>Sua5_start	GTATGTACCTTGGACGACATT
>Sua5-ext-rev	GACGCCCAACCCTATTGAG
>Pcc1_outside-5'	TCCGATCTCTGTTCCACCCA
>Pcc1_outside-3'	ATTTACACGCAACCCCAGGA
>Pcc1_inside-5'	ATGACAAGCAAACGGGAAAAGT
>Pcc1_inside-3'	TCTTGTGGCTTCAAAATCGGG
>Gon7_outside-5'	AGTGTTACGGCCTTGTCAGG
>Gon7_outside-3'	CCGGCAGGATGATTCCAAGT
>Gon7_inside-5'	CGCGATGACCCTCGATACAT
>Gon7_inside-3'	AACAGCATCTTCGTCACCGT
>Cgi121_outside-5'	CATGTTTAATCTTTTGCGCGAA
>Cgi121_outside-3'	ACACTGACTTCACGGAGAACA
>Cgi121_inside-5'	GACCCCCGGTTAGTCTGTTC

>Cgi121_inside-3'	TGTGTTTCTGGTGAGGCTGT
>Bud32_outside-5'	GCGCATCACCAACTTGAAGG
>Bud32_outside-3'	CAATAACTCGGACACGCTTTGATGG
>Bud32_inside-5'	CGGAGGGCACGGTTTTAGTA
>Bud32_inside-3'	CTGAGCCCAGACCGAAATCA
rRNA depletions oligos	
25SLNA*	5BioTEG/GACPCCTZATTLGTETCLATC
rRNA-1	5BioTEG/TGATGCCCCCGACCGTCCCTATTAATCATTACGACCA AGTTTGTCCAAATTCTCCGCTCTGAGA
rRNA-2	5BioTEG/GCTAGCCTGCTATGGTTCAGCGACGCCACAACTGATC AAATGCCCTTCCCTTTCAACAATTTCACG
rRNA-3	5BioTEG/TTCCAGCTCCGCTTCATTGAATAAGTAAAGAACTATT TTGCCGACTTCCCTTATCTACATTATTCTA
rRNA-4	5BioTEG/ATGTCTTCAACCCGGATCAGCCCCGAAGACTTACGTC GCAGTCCTCAGTCCCAGCTGGCAGTATTCCCACAG
rRNA-5	5BioTEG/ATTCTATTATTCCATGCTAATATATTCGAGCAAGCGG TTATCAGTACGACCTGGCATGAAAAC
rRNA-6	5BioTEG/AGCTGCATTCCCAAACAACTCGACTCTTCCCCCACTT CAGTCTTCAAAGTTCTCATTTTTATTCTACACCCTCTATGTCTC TTCACA

Table S3. Genes reduced in RPF in *tcs2*Δ

Systematic Name	Standard Name	Description
YAR066W		Putative GPI protein
YAR070C		Dubious open reading frame
YAR071W	PHO11	One of three repressible acid phosphatases
YBR093C	PHO5	Repressible acid phosphatase
YBR296C	PHO89	Plasma membrane Na+/Pi cotransporter
YCL063W	VAC17	Phosphoprotein involved in vacuole inheritance
YCL064C	CHA1	Catabolic L-serine (L-threonine) deaminase
YCL066W	HMLALPH A1	Silenced copy of ALPHA1 at HML
YCR013C		Dubious open reading frame
YCR040W	MATALPH A1	Transcriptional co-activator that regulates mating-type-specific genes
YDL022W	GPD1	NAD-dependent glycerol-3-phosphate dehydrogenase

YDL042C	SIR2	Conserved NAD+ dependent histone deacetylase of the Sirtuin family
YDL227C	HO	Site-specific endonuclease
YDR111C	ALT2	Catalytically inactive alanine transaminase
YDR140W	MTQ2	S-adenosylmethionine-dependent methyltransferase
YDR184C	ATC1	Nuclear protein
YDR281C	PHM6	Protein of unknown function
YDR354C-A		Dubious open reading frame
YDR365C	ESF1	Nucleolar protein involved in pre-rRNA processing
YDR445C		Dubious open reading frame
YER011W	TIR1	Cell wall mannoprotein
YER072W	VTC1	Subunit of the vacuolar transporter chaperone (VTC) complex
YFL002C	SPB4	Putative ATP-dependent RNA helicase
YFL004W	VTC2	Subunit of vacuolar transporter chaperone (VTC) complex
YFL005W	SEC4	Rab family GTPase
YFL017C	GNA1	Glucosamine-6-phosphate acetyltransferase
YFL017W-A	SMX2	Core Sm protein Sm G
YFL022C	FRS2	Alpha subunit of cytoplasmic phenylalanyl-tRNA synthetase
YFL026W	STE2	Receptor for alpha-factor pheromone
YFL034C-A	RPL22B	Ribosomal 60S subunit protein L22B
YFL045C	SEC53	Phosphomannomutase
YFR001W	LOC1	Nuclear protein involved in asymmetric localization of ASH1 mRNA
YFR005C	SAD1	Conserved zinc-finger domain protein involved in pre-mRNA splicing
YFR016C		Putative protein of unknown function
YFR032C	RRT5	Putative protein of unknown function
YFR032C-A	RPL29	Ribosomal 60S subunit protein L29
YFR032C-B		Putative protein of unknown function
YFR033C	QCR6	Subunit 6 of the ubiquinol cytochrome-c reductase complex
YFR034C	PHO4	Basic helix-loop-helix (bHLH) transcription factor of the myc-family
YFR036W	CDC26	Subunit of the Anaphase-Promoting Complex/Cyclosome (APC/C)
YGL014W	PUF4	Member of the PUF protein family
YGL089C	MF(ALPHA)2	Mating pheromone alpha-factor, made by alpha cells
YGL169W	SUA5	Protein involved in threonylcarbamoyl adenosine biosynthesis
YGL255W	ZRT1	High-affinity zinc transporter of the plasma membrane
YGR035C		Putative protein of unknown function, potential Cdc28p substrate

YGR111W		Putative protein of unknown function
YGR146C	ECL1	Protein of unknown function
YGR177C	ATF2	Alcohol acetyltransferase
YGR210C		Putative protein of unknown function
YGR233C	PHO81	Cyclin-dependent kinase (CDK) inhibitor
YGR234W	YHB1	Nitric oxide oxidoreductase
YHR136C	SPL2	Protein with similarity to cyclin-dependent kinase inhibitors
YHR148W	IMP3	Component of the SSU processome
YHR163W	SOL3	6-phosphogluconolactonase
YHR180W-A		Dubious open reading frame
YHR193C-A		Dubious open reading frame
YHR214C-E		Putative protein of unknown function
YHR214W		Putative protein of unknown function
YHR215W	PHO12	One of three repressible acid phosphatases
YIL011W	TIR3	Cell wall mannoprotein
YIL068W-A		Dubious open reading frame
YJL012C	VTC4	Vacuolar membrane polyphosphate polymerase
YJL056C	ZAP1	Zinc-regulated transcription factor
YJL127C	SPT10	Putative histone acetylase with a role in transcriptional silencing
YJL191W	RPS14B	Protein component of the small (40S) ribosomal subunit
YJR004C	SAG1	Alpha-agglutinin of alpha-cells
YJR047C	ANB1	Translation elongation factor eIF-5A
YJR147W	HMS2	Protein with similarity to heat shock transcription factors
YKL024C	URA6	Uridylate kinase
YKL084W	HOT13	Zinc-binding mitochondrial intermembrane space (IMS) protein
YKL096W	CWP1	Cell wall mannoprotein that localizes to birth scars of daughter cells
YKR025W	RPC37	RNA polymerase III subunit C37
YLL042C	ATG10	Conserved E2-like conjugating enzyme
YLL052C	AQY2	Water channel that mediates water transport across cell membranes
YLR121C	YPS3	Aspartic protease
YLR333C	RPS25B	Protein component of the small (40S) ribosomal subunit
YLR346C		Putative protein of unknown function found in mitochondria
YLR367W	RPS22B	Protein component of the small (40S) ribosomal subunit
YLR452C	SST2	GTPase-activating protein for Gpa1p
YML017W	PSP2	Asn rich cytoplasmic protein that contains RGG motifs
YML058W-A	HUG1	Protein involved in the Mec1p-mediated checkpoint pathway
YML123C	PHO84	High-affinity inorganic phosphate (Pi) transporter

YMR006C	PLB2	Phospholipase B (lysophospholipase) involved in lipid metabolism
YMR141C		Dubious open reading frame
YMR319C	FET4	Low-affinity Fe(II) transporter of the plasma membrane
YNL111C	CYB5	Cytochrome b5
YNL141W	AAH1	Adenine deaminase (adenine aminohydrolase)
YNR044W	AGA1	Anchorage subunit of a-agglutinin of a-cells
YOL013W-B		Dubious open reading frame
YOL068C	HST1	NAD(+)-dependent histone deacetylase
YOL109W	ZEO1	Peripheral membrane protein of the plasma membrane
YOR009W	TIR4	Cell wall mannoprotein
YOR091W	TMA46	Protein of unknown function that associates with translating ribosomes
YOR095C	RKI1	Ribose-5-phosphate ketol-isomerase
YOR238W		Putative protein of unknown function
YOR253W	NAT5	Subunit of protein N-terminal acetyltransferase NatA
YOR299W	BUD7	Member of the ChAPs family (Chs5p-Arf1p-binding proteins)
YOR346W	REV1	Deoxycytidyl transferase
YOR377W	ATF1	Alcohol acetyltransferase
YOR378W	AMF1	Putative paralog of ATR1
YPL019C	VTC3	Subunit of vacuolar transporter chaperone (VTC) complex
YPL049C	DIG1	MAP kinase-responsive inhibitor of the Ste12p transcription factor
YPL067C		Putative protein of unknown function
YPL081W	RPS9A	Protein component of the small (40S) ribosomal subunit
YPL165C	SET6	SET domain protein of unknown function
YPL199C		Putative protein of unknown function
YPL245W		Putative protein of unknown function
YPL263C	KEL3	Cytoplasmic protein of unknown function
YPR013C	CMR3	Putative zinc finger protein
YPR108W-A		Putative protein of unknown function
YPR119W	CLB2	B-type cyclin involved in cell cycle progression

Table S4. Genes increased in RPFs in *tcs2*Δ

Systematic Name	Standard Name	Description
YAL026C	DRS2	Trans-golgi network aminophospholipid translocase (flippase)
YAL037W		Putative protein of unknown function
YAL061W	BDH2	Putative medium-chain alcohol dehydrogenase with similarity to BDH1

YBL017C	PEP1	Type I transmembrane sorting receptor for multiple vacuolar hydrolases
YBL042C	FUI1	High affinity uridine permease, localizes to the plasma membrane
YBL043W	ECM13	Non-essential protein of unknown function
YBL049W	MOH1	Protein of unknown function
YBL066C	SEF1	Putative transcription factor
YBL075C	SSA3	ATPase involved in protein folding and the response to stress
YBL078C	ATG8	Component of autophagosomes and Cvt vesicles
YBL088C	TEL1	Protein kinase primarily involved in telomere length regulation
YBL107C	MIX23	Mitochondrial intermembrane space protein of unknown function
YBR006W	UGA2	Succinate semialdehyde dehydrogenase
YBR015C	MNN2	Alpha-1,2-mannosyltransferase
YBR033W	EDS1	Putative zinc cluster protein, predicted to be a transcription factor
YBR036C	CSG2	Endoplasmic reticulum membrane protein
YBR041W	FAT1	Very long chain fatty acyl-CoA synthetase and fatty acid transporter
YBR043C	QDR3	Multidrug transporter of the major facilitator superfamily
YBR054W	YRO2	Protein of unknown function with similarity to archaeal rhodopsins
YBR072W	HSP26	Small heat shock protein (sHSP) with chaperone activity
YBR097W	VPS15	Serine/threonine protein kinase involved in vacuolar protein sorting
YBR105C	VID24	GID Complex regulatory subunit
YBR139W		Putative serine type carboxypeptidase
YBR147W	RTC2	Putative vacuolar membrane transporter for cationic amino acids
YBR157C	ICS2	Protein of unknown function
YBR168W	PEX32	Peroxisomal integral membrane protein
YBR169C	SSE2	Member of the heat shock protein 70 (HSP70) family
YBR199W	KTR4	Putative mannosyltransferase involved in protein glycosylation
YBR207W	FTH1	Putative high affinity iron transporter
YBR208C	DUR1,2	Urea amidolyase
YBR214W	SDS24	Protein involved in cell separation during budding
YBR229C	ROT2	Glucosidase II catalytic subunit
YBR241C		Putative transporter, member of the sugar porter family
YBR256C	RIB5	Riboflavin synthase
YBR270C	BIT2	Subunit of TORC2 membrane-associated complex
YBR284W		Putative metallo-dependent hydrolase superfamily protein
YBR293W	VBA2	Permease of basic amino acids in the vacuolar membrane
YBR299W	MAL32	Maltase (alpha-D-glucosidase)

YCR011C	ADP1	Putative ATP-dependent permease of the ABC transporter family
YCR017C	CWH43	Putative sensor/transporter protein involved in cell wall biogenesis
YCR023C		Vacuolar membrane protein of unknown function
YCR061W		Protein of unknown function
YDL072C	YET3	Protein of unknown function
YDL174C	DLD1	D-lactate dehydrogenase
YDL204W	RTN2	Reticulon protein
YDR019C	GCV1	T subunit of the mitochondrial glycine decarboxylase complex
YDR070C	FMP16	Protein of unknown function
YDR082W	STN1	Telomere end-binding and capping protein
YDR135C	YCF1	Vacuolar glutathione S-conjugate transporter
YDR216W	ADR1	Carbon source-responsive zinc-finger transcription factor
YDR242W	AMD2	Putative amidase
YDR294C	DPL1	Dihydrosphingosine phosphate lyase
YDR342C	HXT7	High-affinity glucose transporter
YDR343C	HXT6	High-affinity glucose transporter
YDR344C		Dubious open reading frame
YEL009C	GCN4	bZIP transcriptional activator of amino acid biosynthetic genes
YEL011W	GLC3	Glycogen branching enzyme, involved in glycogen accumulation
YEL045C		Dubious open reading frame
YEL065W	SIT1	Ferrioxamine B transporter
YER067W	RGI1	Protein of unknown function
YER069W	ARG5,6	Acetylglutamate kinase and N-acetyl-gamma-glutamyl-phosphate reductase
YER103W	SSA4	Heat shock protein that is highly induced upon stress
YER121W		Putative protein of unknown function
YER150W	SPI1	GPI-anchored cell wall protein involved in weak acid resistance
YER166W	DNF1	Aminophospholipid translocase (flippase)
YER175C	TMT1	Trans-aconitate methyltransferase
YFL014W	HSP12	Plasma membrane protein involved in maintaining membrane organization
YFR053C	HXK1	Hexokinase isoenzyme 1
YGL022W	STT3	Subunit of the oligosaccharyltransferase complex of the ER lumen
YGL062W	PYC1	Pyruvate carboxylase isoform
YGL114W		Putative protein of unknown function
YGL117W		Putative protein of unknown function
YGL121C	GPG1	Proposed gamma subunit of the heterotrimeric G protein
YGL125W	MET13	Major isozyme of methylenetetrahydrofolate reductase

YGL156W	AMS1	Vacuolar alpha mannosidase
YGL184C	STR3	Peroxisomal cystathionine beta-lyase
YGR032W	GSC2	Catalytic subunit of 1,3-beta-glucan synthase
YGR043C	NQM1	Transaldolase of unknown function
YGR088W	CTT1	Cytosolic catalase T
YGR154C	GTO1	Omega-class glutathione transferase
YGR244C	LSC2	Beta subunit of succinyl-CoA ligase
YGR248W	SOL4	6-phosphogluconolactonase
YGR281W	YOR1	Plasma membrane ATP-binding cassette (ABC) transporter
YGR287C	IMA1	Major isomaltase (alpha-1,6-glucosidase/alpha-methylglucosidase)
YHL040C	ARN1	ARN family transporter for siderophore-iron chelates
YHR045W		Putative protein of unknown function
YHR117W	TOM71	Mitochondrial outer membrane protein
YHR210C		Putative aldose 1-epimerase superfamily protein
YIL017C	VID28	GID Complex subunit, serves as adaptor for regulatory subunit Vid24p
YIL029C		Putative protein of unknown function
YIL030C	SSM4	Ubiquitin-protein ligase involved in ER-associated protein degradation
YIL073C	SPO22	Meiosis-specific protein essential for chromosome synapsis
YIL101C	XBP1	Transcriptional repressor
YIL111W	COX5B	Subunit Vb of cytochrome c oxidase
YIL117C	PRM5	Pheromone-regulated protein, predicted to have 1 transmembrane segment
YIL125W	KGD1	Subunit of the mitochondrial alpha-ketoglutarate dehydrogenase complex
YIL129C	TAO3	Component of the RAM signaling network
YIL136W	OM45	Mitochondrial outer membrane protein of unknown function
YIL155C	GUT2	Mitochondrial glycerol-3-phosphate dehydrogenase
YIL164C	NIT1	Nitrilase
YIR016W		Putative protein of unknown function
YIR029W	DAL2	Allantoicase
YIR031C	DAL7	Malate synthase
YIR032C	DAL3	Ureidoglycolate lyase
YIR036C	IRC24	Putative benzil reductase
YIR038C	GTT1	ER associated glutathione S-transferase capable of homodimerization
YIR039C	YPS6	Putative GPI-anchored aspartic protease
YIR042C		Putative protein of unknown function
YJL079C	PRY1	Sterol binding protein involved in the export of acetylated sterols

YJL093C	TOK1	Outward-rectifier potassium channel of the plasma membrane
YJL116C	NCA3	Protein involved in mitochondrion organization
YJL172W	CPS1	Vacuolar carboxypeptidase S
YJR025C	BNA1	3-hydroxyanthranilic acid dioxygenase
YJR109C	CPA2	Large subunit of carbamoyl phosphate synthetase
YJR130C	STR2	Cystathionine gamma-synthase, converts cysteine into cystathionine
YJR154W		Putative protein of unknown function
YKL071W		Putative protein of unknown function
YKL100C		Putative protein of unknown function
YKL161C	KDX1	Protein kinase
YKL187C	FAT3	Protein required for fatty acid uptake
YKL220C	FRE2	Ferric reductase and cupric reductase
YKR022C	NTR2	Essential protein that forms a dimer with Ntr1p
YKR039W	GAP1	General amino acid permease
YKR067W	GPT2	Glycerol-3-phosphate/dihydroxyacetone phosphate sn-1 acyltransferase
YKR076W	ECM4	Omega class glutathione transferase
YKR091W	SRL3	GTB motif (G1/S transcription factor binding) containing protein
YKR093W	PTR2	Integral membrane peptide transporter
YLL015W	BPT1	ABC type transmembrane transporter of MRP/CFTR family
YLL019C	KNS1	Protein kinase involved in negative regulation of PolIII transcription
YLL048C	YBT1	Transporter of the ATP-binding cassette (ABC) family
YLL060C	GTT2	Glutathione S-transferase capable of homodimerization
YLR125W		Putative protein of unknown function
YLR136C	TIS11	mRNA-binding protein expressed during iron starvation
YLR149C		Protein of unknown function
YLR178C	TFS1	Protein that interacts with and inhibits carboxypeptidase Y and Ira2p
YLR251W	SYM1	Protein required for ethanol metabolism
YLR258W	GSY2	Glycogen synthase
YLR327C	TMA10	Protein of unknown function that associates with ribosomes
YLR383W	SMC6	Component of the SMC5-SMC6 complex
YLR454W	FMP27	Putative protein of unknown function
YML023C	NSE5	Component of the SMC5-SMC6 complex
YML091C	RPM2	Protein subunit of mitochondrial RNase P
YML100W	TSL1	Large subunit of trehalose 6-phosphate synthase/phosphatase complex
YML116W	ATR1	Multidrug efflux pump of the major facilitator superfamily

YML128C	MSC1	Protein of unknown function
YMR008C	PLB1	Phospholipase B (lysophospholipase) involved in lipid metabolism
YMR062C	ARG7	Mitochondrial ornithine acetyltransferase
YMR095C	SNO1	Protein of unconfirmed function
YMR096W	SNZ1	Protein involved in vitamin B6 biosynthesis
YMR105C	PGM2	Phosphoglucomutase
YMR135C	GID8	Subunit of GID Complex, binds strongly to central component Vid30p
YMR136W	GAT2	Protein containing GATA family zinc finger motifs
YMR189W	GCV2	P subunit of the mitochondrial glycine decarboxylase complex
YMR196W		Putative protein of unknown function
YMR323W	ERR3	Enolase, a phosphopyruvate hydratase
YNL018C		Putative protein of unknown function
YNL034W		Putative protein of unknown function
YNL036W	NCE103	Carbonic anhydrase
YNL104C	LEU4	Alpha-isopropylmalate synthase (2-isopropylmalate synthase)
YNL142W	MEP2	Ammonium permease involved in regulation of pseudohyphal growth
YNL160W	YGP1	Cell wall-related secretory glycoprotein
YNL200C		NADHX epimerase
YNL202W	SPS19	Peroxisomal 2,4-dienoyl-CoA reductase
YNL237W	YTP1	Probable type-III integral membrane protein of unknown function
YNL332W	THI12	Protein involved in synthesis of the thiamine precursor HMP
YNR001C	CIT1	Citrate synthase
YNR002C	ATO2	Putative transmembrane protein involved in export of ammonia
YNR034W-A		Putative protein of unknown function
YNR067C	DSE4	Daughter cell-specific secreted protein with similarity to glucanases
YNR069C	BSC5	Protein of unknown function
YOL058W	ARG1	Arginosuccinate synthetase
YOL119C	MCH4	Protein with similarity to mammalian monocarboxylate permeases
YOL126C	MDH2	Cytoplasmic malate dehydrogenase
YOR120W	GCY1	Glycerol dehydrogenase
YOR130C	ORT1	Ornithine transporter of the mitochondrial inner membrane
YOR173W	DCS2	m(7)GpppX pyrophosphatase regulator
YOR185C	GSP2	GTP binding protein (mammalian Ranp homolog)
YOR289W		Putative protein of unknown function
YOR303W	CPA1	Small subunit of carbamoyl phosphate synthetase

YOR374W	ALD4	Mitochondrial aldehyde dehydrogenase
YOR393W	ERR1	Protein of unknown function
YPL006W	NCR1	Vacuolar membrane protein
YPL022W	RAD1	Single-stranded DNA endonuclease (with Rad10p)
YPL036W	PMA2	Plasma membrane H+-ATPase
YPL148C	PPT2	Phosphopantetheine:protein transferase (PPTase)
YPL154C	PEP4	Vacuolar aspartyl protease (proteinase A)
YPL167C	REV3	Catalytic subunit of DNA polymerase zeta
YPL186C	UIP4	Protein that interacts with Ulp1p
YPL214C	THI6	Thiamine-phosphate diphosphorylase and hydroxyethylthiazole kinase
YPL230W	USV1	Putative transcription factor containing a C2H2 zinc finger
YPL281C	ERR2	Enolase, a phosphopyruvate hydratase
YPR002W	PDH1	Mitochondrial protein that participates in respiration
YPR085C	ASA1	Subunit of the ASTRA complex, involved in chromatin remodeling
YPR091C	NVJ2	Lipid-binding ER protein, enriched at nucleus-vacuolar junctions (NVJ)
YPR160W	GPH1	Glycogen phosphorylase required for the mobilization of glycogen
YPR184W	GDB1	Glycogen debranching enzyme

Table S5. Genes with increased RPFs in *tcs2*Δ under *GCN4* regulation

Systematic Name	Standard Name	Description
YBR043C	QDR3	Multidrug transporter of the major facilitator superfamily
YBR256C	RIB5	Riboflavin synthase
YER069W	ARG5,6	Acetylglutamate kinase and N-acetyl-gamma-glutamyl-phosphate reductase
YGL125W	MET13	Major isozyme of methylenetetrahydrofolate reductase
YGL184C	STR3	Peroxisomal cystathionine beta-lyase
YJR025C	BNA1	3-hydroxyanthranilic acid dioxygenase
YJR109C	CPA2	Large subunit of carbamoyl phosphate synthetase
YMR062C	ARG7	Mitochondrial ornithine acetyltransferase
YMR095C	SNO1	Protein of unconfirmed function
YMR096W	SNZ1	Protein involved in vitamin B6 biosynthesis
YNL104C	LEU4	Alpha-isopropylmalate synthase (2-isopropylmalate synthase)
YOL058W	ARG1	Arginosuccinate synthetase
YOL119C	MCH4	Protein with similarity to mammalian monocarboxylate permeases

YOR130C	ORT1	Ornithine transporter of the mitochondrial inner membrane
YOR303W	CPA1	Small subunit of carbamoyl phosphate synthetase

Table S6. ORFs increased in translational ambiguities in BY4742

Systematic Name	Standard Name	Description
YBL003C	HTA2	Histone H2A
YBL051C	PIN4	Protein involved in G2/M phase progression and response to DNA damage
YBL087C	RPL23A	Ribosomal 60S subunit protein L23A
YBR017C	KAP104	Transportin or cytosolic karyopherin beta 2
YBR031W	RPL4A	Ribosomal 60S subunit protein L4A
YBR057C	MUM2	Protein essential for meiotic DNA replication and sporulation
YBR058C	UBP14	Ubiquitin-specific protease
YBR084C-A	RPL19A	Ribosomal 60S subunit protein L19A
YBR090C		Putative protein of unknown function
YBR118W	TEF2	Translational elongation factor EF-1 alpha
YBR126W-B		Dubious open reading frame
YBR279W	PAF1	Component of the Paf1p complex involved in transcription elongation
YCL005W-A	VMA9	Vacuolar H+ ATPase subunit e of the V-ATPase V0 subcomplex
YCR005C	CIT2	Citrate synthase
YCR024C-A	PMP1	Regulatory subunit for the plasma membrane H(+)-ATPase Pma1p
YCR034W	FEN1	Fatty acid elongase, involved in sphingolipid biosynthesis
YCR065W	HCM1	Forkhead transcription factor
YDL140C	RPO21	RNA polymerase II largest subunit B220
YDL184C	RPL41A	Ribosomal 60S subunit protein L41A
YDL191W	RPL35A	Ribosomal 60S subunit protein L35A
YDL195W	SEC31	Component of the Sec13p-Sec31p complex of the COPII vesicle coat
YDL232W	OST4	Subunit of the oligosaccharyltransferase complex of the ER lumen
YDR074W	TPS2	Phosphatase subunit of the trehalose-6-P synthase/phosphatase complex
YDR119W	VBA4	Protein of unknown function
YDR154C		Dubious open reading frame
YDR172W	SUP35	Translation termination factor eRF3
YDR320C-A	DAD4	Essential subunit of the Dam1 complex (aka DASH complex)

YDR433W		Dubious open reading frame
YDR508C	GNP1	High-affinity glutamine permease
YDR510W	SMT3	Ubiquitin-like protein of the SUMO family
YEL027W	VMA3	Proteolipid subunit c of the V0 domain of vacuolar H(+)-ATPase
YER074W	RPS24A	Protein component of the small (40S) ribosomal subunit
YER151C	UBP3	Ubiquitin-specific protease involved in transport and osmotic response
YGL088W		Dubious open reading frame
YGL207W	SPT16	Subunit of the heterodimeric FACT complex (Spt16p-Pob3p)
YGL225W	VRG4	Golgi GDP-mannose transporter
YGR001C	AML1	Putative protein of unknown function
YGR119C	NUP57	FG-nucleoporin component of central core of the nuclear pore complex
YGR148C	RPL24B	Ribosomal 60S subunit protein L24B
YGR229C	SMI1	Protein involved in the regulation of cell wall synthesis
YHL007C	STE20	Cdc42p-activated signal transducing kinase
YHR066W	SSF1	Constituent of 66S pre-ribosomal particles
YHR136C	SPL2	Protein with similarity to cyclin-dependent kinase inhibitors
YHR152W	SPO12	Nucleolar protein of unknown function
YHR203C	RPS4B	Protein component of the small (40S) ribosomal subunit
YJL081C	ARP4	Nuclear actin-related protein involved in chromatin remodeling
YJL098W	SAP185	Protein that forms a complex with the Sit4p protein phosphatase
YJL136C	RPS21B	Protein component of the small (40S) ribosomal subunit
YJR001W	AVT1	Vacuolar transporter
YJR094W-A	RPL43B	Ribosomal 60S subunit protein L43B
YKL006W	RPL14A	Ribosomal 60S subunit protein L14A
YKL029C	MAE1	Mitochondrial malic enzyme
YKL054C	DEF1	RNAPII degradation factor
YKL145W	RPT1	ATPase of the 19S regulatory particle of the 26S proteasome
YKL160W	ELF1	Transcription elongation factor with a conserved zinc finger domain
YKL179C	COY1	Golgi membrane protein with similarity to mammalian CASP
YKR059W	TIF1	Translation initiation factor eIF4A
YKR093W	PTR2	Integral membrane peptide transporter
YLR095C	IOC2	Subunit of the Isw1b complex
YLR216C	CPR6	Peptidyl-prolyl cis-trans isomerase (cyclophilin)
YLR262C	YPT6	Rab family GTPase
YLR287C		Putative protein of unknown function
YLR287C-A	RPS30A	Protein component of the small (40S) ribosomal subunit
YLR333C	RPS25B	Protein component of the small (40S) ribosomal subunit

YLR372W	SUR4	Elongase
YLR409C	UTP21	Subunit of U3-containing 90S preribosome and SSU processome complexes
YMR093W	UTP15	Nucleolar protein
YMR145C	NDE1	Mitochondrial external NADH dehydrogenase
YNL037C	IDH1	Subunit of mitochondrial NAD(+)-dependent isocitrate dehydrogenase
YNL066W	SUN4	Cell wall protein related to glucanases
YNL068C	FKH2	Forkhead family transcription factor
YNL162W	RPL42A	Ribosomal 60S subunit protein L42A
YNL244C	SUI1	Translation initiation factor eIF1
YNL327W	EGT2	Glycosylphosphatidylinositol (GPI)-anchored cell wall endoglucanase
YOL101C	IZH4	Membrane protein involved in zinc ion homeostasis
YOR057W	SGT1	Cochaperone protein
YOR182C	RPS30B	Protein component of the small (40S) ribosomal subunit
YOR202W	HIS3	Imidazoleglycerol-phosphate dehydratase
YOR239W	ABP140	AdoMet-dependent tRNA methyltransferase and actin binding protein
YOR309C		Dubious open reading frame
YOR312C	RPL20B	Ribosomal 60S subunit protein L20B
YPL082C	MOT1	Essential protein involved in regulation of transcription
YPL183C	RTT10	WD40 domain-containing protein involved in endosomal recycling
YPL256C	CLN2	G1 cyclin involved in regulation of the cell cycle
YPR053C		Dubious open reading frame
YPR080W	TEF1	Translational elongation factor EF-1 alpha
YPR189W	SKI3	Ski complex component and TPR protein

Table S7. ORFs increased in translational ambiguities in *tcs2*Δ

Systematic Name	Standard Name	Description
YAL005C	SSA1	ATPase involved in protein folding and NLS-directed nuclear transport
YAL042 W	ERV46	Protein localized to COPII-coated vesicles
YAL044C	GCV3	H subunit of the mitochondrial glycine decarboxylase complex
YAR028 W		Putative integral membrane protein

YBL002W	HTB2	Histone H2B
YBL003C	HTA2	Histone H2A
YBL028C		Protein of unknown function that may interact with ribosomes
YBL031W	SHE1	Mitotic spindle protein
YBL051C	PIN4	Protein involved in G2/M phase progression and response to DNA damage
YBL079W	NUP170	Subunit of the inner ring of the nuclear pore complex (NPC)
YBL087C	RPL23A	Ribosomal 60S subunit protein L23A
YBL107C	MIX23	Mitochondrial intermembrane space protein of unknown function
YBR010W	HHT1	Histone H3
YBR017C	KAP104	Transportin or cytosolic karyopherin beta 2
YBR031W	RPL4A	Ribosomal 60S subunit protein L4A
YBR038W	CHS2	Chitin synthase II
YBR048W	RPS11B	Protein component of the small (40S) ribosomal subunit
YBR055C	PRP6	Splicing factor
YBR084C-A	RPL19A	Ribosomal 60S subunit protein L19A
YBR086C	IST2	Cortical ER protein involved in ER-plasma membrane tethering
YBR090C		Putative protein of unknown function
YBR102C	EXO84	Exocyst subunit with dual roles in exocytosis and spliceosome assembly
YBR111W-A	SUS1	Component of both the SAGA histone acetylase and TREX-2 complexes
YBR112C	CYC8	General transcriptional co-repressor
YBR118W	TEF2	Translational elongation factor EF-1 alpha
YBR126C	TPS1	Synthase subunit of trehalose-6-P synthase/phosphatase complex
YBR126W-B		Dubious open reading frame
YBR146W	MRPS9	Mitochondrial ribosomal protein of the small subunit
YBR155W	CNS1	TPR-containing co-chaperone
YBR158W	AMN1	Protein required for daughter cell separation
YBR160W	CDC28	Cyclin-dependent kinase (CDK) catalytic subunit
YBR172C	SMY2	GYF domain protein

YBR177C	EHT1	Acyl-coenzymeA:ethanol O-acyltransferase
YBR207W	FTH1	Putative high affinity iron transporter
YBR263W	SHM1	Mitochondrial serine hydroxymethyltransferase
YBR268W	MRPL37	Mitochondrial ribosomal protein of the large subunit
YBR289W	SNF5	Subunit of the SWI/SNF chromatin remodeling complex
YCL005W-A	VMA9	Vacuolar H+ ATPase subunit e of the V-ATPase V0 subcomplex
YCL008C	STP22	Component of the ESCRT-I complex
YCL042W		Putative protein of unknown function
YCR002C	CDC10	Component of the septin ring, required for cytokinesis
YCR005C	CIT2	Citrate synthase
YCR034W	FEN1	Fatty acid elongase, involved in sphingolipid biosynthesis
YCR082W	AHC2	Component of the ADA histone acetyltransferase complex
YDL053C	PBP4	Pbp1p binding protein
YDL067C	COX9	Subunit VIIa of cytochrome c oxidase (Complex IV)
YDL092W	SRP14	Signal recognition particle (SRP) subunit
YDL125C	HNT1	Adenosine 5'-monophosphoramidase
YDL132W	CDC53	Cullin
YDL173W	PAR32	Putative protein of unknown function
YDL191W	RPL35A	Ribosomal 60S subunit protein L35A
YDL195W	SEC31	Component of the Sec13p-Sec31p complex of the COPII vesicle coat
YDL198C	GGC1	Mitochondrial GTP/GDP transporter
YDL229W	SSB1	Cytoplasmic ATPase that is a ribosome-associated molecular chaperone
YDL232W	OST4	Subunit of the oligosaccharyltransferase complex of the ER lumen
YDR025W	RPS11A	Protein component of the small (40S) ribosomal subunit
YDR046C	BAP3	Amino acid permease
YDR074W	TPS2	Phosphatase subunit of the trehalose-6-P synthase/phosphatase complex

YDR119 W	VBA4	Protein of unknown function
YDR120C	TRM1	tRNA methyltransferase
YDR128 W	MTC5	Subunit of the SEA (Seh1-associated) complex
YDR135C	YCF1	Vacuolar glutathione S-conjugate transporter
YDR141C	DOP1	Golgi-localized, leucine-zipper domain containing protein
YDR154C		Dubious open reading frame
YDR172 W	SUP35	Translation termination factor eRF3
YDR208 W	MSS4	Phosphatidylinositol-4-phosphate 5-kinase
YDR276C	PMP3	Small plasma membrane protein
YDR309C	GIC2	Redundant rho-like GTPase Cdc42p effector
YDR372C	VPS74	Golgi phosphatidylinositol-4-kinase effector and PtdIns4P sensor
YDR505C	PSP1	Asn and gln rich protein of unknown function
YDR510 W	SMT3	Ubiquitin-like protein of the SUMO family
YDR516C	EMI2	Non-essential protein of unknown function
YEL017W	GTT3	Protein of unknown function may be involved in glutathione metabolism
YEL027W	VMA3	Proteolipid subunit c of the V0 domain of vacuolar H(+)-ATPase
YEL046C	GLY1	Threonine aldolase
YER056C -A	RPL34A	Ribosomal 60S subunit protein L34A
YER089C	PTC2	Type 2C protein phosphatase (PP2C)
YER102W	RPS8B	Protein component of the small (40S) ribosomal subunit
YER127W	LCP5	Essential protein involved in maturation of 18S rRNA
YER131W	RPS26B	Protein component of the small (40S) ribosomal subunit
YER151C	UBP3	Ubiquitin-specific protease involved in transport and osmotic response
YER166W	DNF1	Aminophospholipid translocase (flippase)
YFL004W	VTC2	Subunit of vacuolar transporter chaperone (VTC) complex
YFL010C	WWM1	WW domain containing protein of unknown function
YFR030W	MET10	Subunit alpha of assimilatory sulfite reductase
YFR044C	DUG1	Cys-Gly metallo-di-peptidase
YFR050C	PRE4	Beta 7 subunit of the 20S proteasome
YGL031C	RPL24A	Ribosomal 60S subunit protein L24A
YGL062 W	PYC1	Pyruvate carboxylase isoform
YGL088 W		Dubious open reading frame

YGL120C	PRP43	RNA helicase in the DEAH-box family
YGL130 W	CEG1	Guanylyltransferase involved in mRNA 5' capping
YGL137 W	SEC27	Essential beta'-coat protein of the COPI coatomer
YGL141 W	HUL5	Multiubiquitin chain assembly factor (E4)
YGL189C	RPS26A	Protein component of the small (40S) ribosomal subunit
YGL207 W	SPT16	Subunit of the heterodimeric FACT complex (Spt16p-Pob3p)
YGL225 W	VRG4	Golgi GDP-mannose transporter
YGL245 W	GUS1	Glutamyl-tRNA synthetase (GluRS)
YGR001C	AML1	Putative protein of unknown function
YGR054 W		Eukaryotic initiation factor (eIF) 2A
YGR092 W	DBF2	Ser/Thr kinase involved in transcription and stress response
YGR094 W	VAS1	Mitochondrial and cytoplasmic valyl-tRNA synthetase
YGR118 W	RPS23A	Ribosomal protein 28 (rp28) of the small (40S) ribosomal subunit
YGR119C	NUP57	FG-nucleoporin component of central core of the nuclear pore complex
YGR148C	RPL24B	Ribosomal 60S subunit protein L24B
YGR183C	QCR9	Subunit 9 of ubiquinol cytochrome-c reductase (Complex III)
YGR197C	SNG1	Protein involved in resistance to nitrosoguanidine and 6-azauracil
YGR200C	ELP2	Subunit of Elongator complex
YGR229C	SMI1	Protein involved in the regulation of cell wall synthesis
YGR234 W	YHB1	Nitric oxide oxidoreductase
YGR235C	MIC26	Component of the MICOS complex
YHL001 W	RPL14B	Ribosomal 60S subunit protein L14B
YHR005C -A	TIM10	Essential protein of the mitochondrial intermembrane space
YHR010 W	RPL27A	Ribosomal 60S subunit protein L27A
YHR016C	YSC84	Actin-binding protein
YHR042 W	NCP1	NADP-cytochrome P450 reductase

YHR047C	AAP1	Arginine/alanine amino peptidase
YHR066 W	SSF1	Constituent of 66S pre-ribosomal particles
YHR077C	NMD2	Protein involved in the nonsense-mediated mRNA decay (NMD) pathway
YHR113 W	APE4	Cytoplasmic aspartyl aminopeptidase with possible vacuole function
YHR143 W	DSE2	Daughter cell-specific secreted protein with similarity to glucanases
YHR143 W-A	RPC10	RNA polymerase subunit ABC10-alpha, found in RNA pol I, II, and III
YHR152 W	SPO12	Nucleolar protein of unknown function
YHR174 W	ENO2	Enolase II, a phosphopyruvate hydratase
YIL009C-A	EST3	Component of the telomerase holoenzyme
YIL018W	RPL2B	Ribosomal 60S subunit protein L2B
YIL035C	CKA1	Alpha catalytic subunit of casein kinase 2 (CK2)
YIL038C	NOT3	Subunit of CCR4-NOT global transcriptional regulator
YIL050W	PCL7	Pho85p cyclin of the Pho80p subfamily
YIL125W	KGD1	Subunit of the mitochondrial alpha-ketoglutarate dehydrogenase complex
YIL135C	VHS2	Regulator of septin dynamics
YIL148W	RPL40A	Ubiquitin-ribosomal 60S subunit protein L40A fusion protein
YIR001C	SGN1	Cytoplasmic RNA-binding protein
YIR015W	RPR2	Subunit of nuclear RNase P
YIR036C	IRC24	Putative benzil reductase
YJL012C	VTC4	Vacuolar membrane polyphosphate polymerase
YJL078C	PRY3	Cell wall-associated protein involved in export of acetylated sterols
YJL080C	SCP160	Essential RNA-binding G protein effector of mating response pathway
YJL136C	RPS21B	Protein component of the small (40S) ribosomal subunit
YJL143W	TIM17	Essential component of the TIM23 complex
YJL173C	RFA3	Subunit of heterotrimeric Replication Protein A (RPA)
YJL176C	SWI3	Subunit of the SWI/SNF chromatin remodeling complex
YJL177W	RPL17B	Ribosomal 60S subunit protein L17B
YJR001W	AVT1	Vacuolar transporter
YJR005W	APL1	Beta-adaptin
YJR076C	CDC11	Component of the septin ring that is required for cytokinesis
YJR094W -A	RPL43B	Ribosomal 60S subunit protein L43B

YJR145C	RPS4A	Protein component of the small (40S) ribosomal subunit
YKL014C	URB1	Protein required for the normal accumulation of 25S and 5.8S rRNAs
YKL054C	DEF1	RNAPII degradation factor
YKL085W	MDH1	Mitochondrial malate dehydrogenase
YKL141W	SDH3	Subunit of succinate dehydrogenase and of TIM22 translocase
YKL145W	RPT1	ATPase of the 19S regulatory particle of the 26S proteasome
YKL152C	GPM1	Tetrameric phosphoglycerate mutase
YKL179C	COY1	Golgi membrane protein with similarity to mammalian CASP
YKL184W	SPE1	Ornithine decarboxylase
YKL213C	DOA1	WD repeat protein required for ubiquitin-mediated protein degradation
YKL216W	URA1	Dihydroorotate dehydrogenase
YKR077W	MSA2	Putative transcriptional activator
YKR089C	TGL4	Multifunctional lipase/hydrolase/phospholipase
YKR094C	RPL40B	Ubiquitin-ribosomal 60S subunit protein L40B fusion protein
YLR003C	CMS1	Putative subunit of the 90S preribosome processome complex
YLR017W	MEU1	Methylthioadenosine phosphorylase (MTAP)
YLR027C	AAT2	Cytosolic aspartate aminotransferase involved in nitrogen metabolism
YLR052W	IES3	Subunit of the INO80 chromatin remodeling complex
YLR058C	SHM2	Cytosolic serine hydroxymethyltransferase
YLR095C	IOC2	Subunit of the Isw1b complex
YLR209C	PNP1	Purine nucleoside phosphorylase
YLR216C	CPR6	Peptidyl-prolyl cis-trans isomerase (cyclophilin)
YLR256W	HAP1	Zinc finger transcription factor
YLR259C	HSP60	Tetradecameric mitochondrial chaperonin
YLR262C	YPT6	Rab family GTPase
YLR274W	MCM5	Component of the Mcm2-7 hexameric helicase complex
YLR286C	CTS1	Endochitinase
YLR388W	RPS29A	Protein component of the small (40S) ribosomal subunit
YLR389C	STE23	Metalloprotease
YLR432W	IMD3	Inosine monophosphate dehydrogenase
YML024W	RPS17A	Ribosomal protein 51 (rp51) of the small (40s) subunit
YML048W	GSF2	Endoplasmic reticulum (ER) localized integral membrane protein

YML086C	ALO1	D-Arabinono-1,4-lactone oxidase
YMR033 W	ARP9	Component of both the SWI/SNF and RSC chromatin remodeling complexes
YMR061 W	RNA14	Component of the cleavage and polyadenylation factor I (CF I)
YMR093 W	UTP15	Nucleolar protein
YMR122W-A		Protein of unknown function
YMR136W	GAT2	Protein containing GATA family zinc finger motifs
YMR149W	SWP1	Delta subunit of the oligosaccharyl transferase glycoprotein complex
YMR186W	HSC82	Cytoplasmic chaperone of the Hsp90 family
YMR194W	RPL36A	Ribosomal 60S subunit protein L36A
YMR247C	RKR1	RING domain E3 ubiquitin ligase
YMR261C	TPS3	Regulatory subunit of trehalose-6-phosphate synthase/phosphatase
YNL039W	BDP1	Essential subunit of RNA polymerase III transcription factor (TFIIIB)
YNL049C	SFB2	Component of the Sec23p-Sfb2p heterodimer of the COPII vesicle coat
YNL055C	POR1	Mitochondrial porin (voltage-dependent anion channel)
YNL065W	AQR1	Plasma membrane transporter of the major facilitator superfamily
YNL066W	SUN4	Cell wall protein related to glucanases
YNL068C	FKH2	Forkhead family transcription factor
YNL074C	MLF3	Serine-rich protein of unknown function
YNL085W	MKT1	Protein that forms a complex with Pbp1p
YNL098C	RAS2	GTP-binding protein
YNL103W	MET4	Leucine-zipper transcriptional activator
YNL110C	NOP15	Constituent of 66S pre-ribosomal particles
YNL118C	DCP2	Catalytic subunit of the Dcp1p-Dcp2p decapping enzyme complex
YNL124W	NAF1	RNA-binding protein required for the assembly of box H/ACA snoRNPs
YNL162W	RPL42A	Ribosomal 60S subunit protein L42A
YNL183C	NPR1	Protein kinase
YNL190W		Hydrophilin essential in desiccation-rehydration process
YNL197C	WHI3	RNA binding protein that sequesters CLN3 mRNA in cytoplasmic foci
YNL209W	SSB2	Cytoplasmic ATPase that is a ribosome-associated molecular chaperone
YNL231C	PDR16	Phosphatidylinositol transfer protein (PITP)
YNL233W	BNI4	Targeting subunit for Glc7p protein phosphatase
YNL244C	SUI1	Translation initiation factor eIF1
YNL300W	TOS6	Glycosylphosphatidylinositol-dependent cell wall protein
YNL307C	MCK1	Dual-specificity ser/thr and tyrosine protein kinase

YNL313C	EMW1	Essential conserved protein with a role in cell wall integrity
YNL327W	EGT2	Glycosylphosphatidylinositol (GPI)-anchored cell wall endoglucanase
YNR034W	SOL1	Protein with a possible role in tRNA export
YNR035C	ARC35	Subunit of the ARP2/3 complex
YNR047W	FPK1	Ser/Thr protein kinase
YNR052C	POP2	RNase of the DEDD superfamily
YOL010W	RCL1	Endonuclease that cleaves pre-rRNA at site A2 for 18S rRNA biogenesis
YOL076W	MDM20	Non-catalytic subunit of the NatB N-terminal acetyltransferase
YOL121C	RPS19A	Protein component of the small (40S) ribosomal subunit
YOR014W	RTS1	B-type regulatory subunit of protein phosphatase 2A (PP2A)
YOR048C	RAT1	Nuclear 5' to 3' single-stranded RNA exonuclease
YOR057W	SGT1	Cochaperone protein
YOR061W	CKA2	Alpha' catalytic subunit of casein kinase 2 (CK2)
YOR069W	VPS5	Nexin-1 homolog
YOR075W	UFE1	t-SNARE protein required for retrograde vesicular traffic
YOR141C	ARP8	Nuclear actin-related protein involved in chromatin remodeling
YOR182C	RPS30B	Protein component of the small (40S) ribosomal subunit
YOR189W	IES4	Component of the INO80 chromatiin remodeling complex
YOR195W	SLK19	Kinetochore-associated protein
YOR202W	HIS3	Imidazoleglycerol-phosphate dehydratase
YOR234C	RPL33B	Ribosomal 60S subunit protein L33B
YOR239W	ABP140	AdoMet-dependent tRNA methyltransferase and actin binding protein
YOR241W	MET7	Folylpolyglutamate synthetase
YOR247W	SRL1	Mannoprotein that exhibits a tight association with the cell wall
YOR309C		Dubious open reading frame
YOR312C	RPL20B	Ribosomal 60S subunit protein L20B
YOR332W	VMA4	Subunit E of the V1 domain of the vacuolar H+-ATPase (V-ATPase)
YPL058C	PDR12	Plasma membrane ATP-binding cassette (ABC) transporter
YPL061W	ALD6	Cytosolic aldehyde dehydrogenase
YPL105C	SYH1	Protein of unknown function that influences nuclear pore distribution
YPL126W	NAN1	U3 snoRNP protein
YPL143W	RPL33A	Ribosomal 60S subunit protein L33A
YPL184C	MRN1	RNA-binding protein that may be involved in translational regulation
YPL195W	APL5	Delta adaptin-like subunit of the clathrin associated protein complex
YPL204W	HRR25	Protein kinase
YPL249C-A	RPL36B	Ribosomal 60S subunit protein L36B
YPL256C	CLN2	G1 cyclin involved in regulation of the cell cycle
YPR019W	MCM4	Essential helicase component of heterohexameric MCM2-7 complexes
YPR036W-		Protein of unknown function

A		
YPR051W	MAK3	Catalytic subunit of the NatC type N-terminal acetyltransferase
YPR053C		Dubious open reading frame
YPR062W	FCY1	Cytosine deaminase
YPR063C		ER-localized protein of unknown function
YPR069C	SPE3	Spermidine synthase
YPR072W	NOT5	Subunit of CCR4-NOT global transcriptional regulator
YPR080W	TEF1	Translational elongation factor EF-1 alpha
YPR086W	SUA7	Transcription factor TFIIB
YPR103W	PRE2	Beta 5 subunit of the 20S proteasome
YPR129W	SCD6	Repressor of translation initiation
YPR132W	RPS23B	Ribosomal protein 28 (rp28) of the small (40S) ribosomal subunit
YPR149W	NCE102	Protein of unknown function
YPR163C	TIF3	Translation initiation factor eIF-4B
YPR170W-B		Putative protein of unknown function
YPR173C	VPS4	AAA-ATPase involved in multivesicular body (MVB) protein sorting
YPR187W	RPO26	RNA polymerase subunit ABC23

Table S8. Initiation at upstream UUG in BY4742

Systematic Name	Standard Name	Description
YAL012W	CYS3	Cystathionine gamma-lyase
YBL029C-A		Protein of unknown function
YBL039C	URA7	Major CTP synthase isozyme (see also URA8)
YBL060W	YEL1	Guanine nucleotide exchange factor specific for Arf3p
YBR028C	YPK3	AGC kinase
YBR121C	GRS1	Cytoplasmic and mitochondrial glycyl-tRNA synthase
YBR121C-A		Dubious open reading frame
YBR168W	PEX32	Peroxisomal integral membrane protein
YBR193C	MED8	Subunit of the RNA polymerase II mediator complex
YBR212W	NGR1	RNA binding protein that negatively regulates growth rate
YBR221C	PDB1	E1 beta subunit of the pyruvate dehydrogenase (PDH) complex
YCR096C	HMRA2	Silenced copy of a2 at HMR
YDL003W	MCD1	Essential alpha-kleisin subunit of the cohesin complex
YDL025C	RTK1	Putative protein kinase, potentially phosphorylated by Cdc28p
YDL028C	MPS1	Dual-specificity kinase

YDL063C	SYO1	Transport adaptor or symportin
YDL129W		Protein of unknown function
YDL177C		Putative protein of unknown function
YDL208W	NHP2	Protein related to mammalian high mobility group (HMG) proteins
YDR037W	KRS1	Lysyl-tRNA synthetase
YDR043C	NRG1	Transcriptional repressor
YDR086C	SSS1	Subunit of the Sec61p translocation complex (Sec61p-Sss1p-Sbh1p)
YDR159W	SAC3	mRNA export factor
YDR189W	SLY1	Hydrophilic protein involved in ER/Golgi vesicle trafficking
YDR319C	YFT2	Protein required for normal ER membrane biosynthesis
YDR347W	MRP1	Mitochondrial ribosomal protein of the small subunit
YDR377W	ATP17	Subunit f of the F0 sector of mitochondrial F1F0 ATP synthase
YDR433W		Dubious open reading frame
YDR473C	PRP3	Splicing factor
YDR514C		Protein of unknown function that localizes to mitochondria
YDR530C	APA2	Diadenosine 5',5'''-P1,P4-tetraphosphate phosphorylase II
YEL063C	CAN1	Plasma membrane arginine permease
YEL072W	RMD6	Protein required for sporulation
YER059W	PCL6	Pho85p cyclin of the Pho80p subfamily
YER090W	TRP2	Anthranilate synthase
YER169W	RPH1	JmjC domain-containing histone demethylase
YFL007W	BLM10	Proteasome activator
YFR014C	CMK1	Calmodulin-dependent protein kinase
YFR049W	YMR31	Mitochondrial ribosomal protein of the small subunit
YGL037C	PNC1	Nicotinamidase that converts nicotinamide to nicotinic acid
YGL144C	ROG1	Protein with putative serine active lipase domain
YGL179C	TOS3	Protein kinase
YGR026W		Putative protein of unknown function
YGR028W	MSP1	Mitochondrial protein involved in mitochondrial protein sorting
YGR091W	PRP31	Splicing factor
YGR155W	CYS4	Cystathionine beta-synthase
YGR166W	TRS65	Component of transport protein particle (TRAPP) complex II
YGR177C	ATF2	Alcohol acetyltransferase
YGR188C	BUB1	Protein kinase involved in the cell cycle checkpoint into anaphase
YGR244C	LSC2	Beta subunit of succinyl-CoA ligase
YGR260W	TNA1	High affinity nicotinic acid plasma membrane permease
YGR267C	FOL2	GTP-cyclohydrolase I
YHL010C	ETP1	Putative protein of unknown function required for growth on ethanol
YHL032C	GUT1	Glycerol kinase
YHR039C	MSC7	Protein of unknown function

YHR072W-A	NOP10	Subunit of box H/ACA snoRNP complex
YHR084W	STE12	Transcription factor that is activated by a MAPK signaling cascade
YHR131C		Putative protein of unknown function
YHR172W	SPC97	Component of the microtubule-nucleating Tub4p (gamma-tubulin) complex
YHR186C	KOG1	Subunit of TORC1
YHR206W	SKN7	Nuclear response regulator and transcription factor
YIL016W	SNL1	Ribosome-associated protein
YIL103W	DPH1	Protein required for synthesis of diphthamide
YIL133C	RPL16A	Ribosomal 60S subunit protein L16A
YJL016W		Putative protein of unknown function
YJL046W	AIM22	Putative lipoate-protein ligase
YJL130C	URA2	Bifunctional carbamoylphosphate synthetase/aspartate transcarbamylase
YJL146W	IDS2	Protein involved in modulation of Ime2p activity during meiosis
YJR034W	PET191	Protein required for assembly of cytochrome c oxidase
YJR054W	KCH1	Potassium transporter that mediates K+ influx
YKL004W	AUR1	Phosphatidylinositol:ceramide phosphoinositol transferase
YKL076C	PSY1	Dubious open reading frame
YKL092C	BUD2	GTPase activating factor for Rsr1p/Bud1p
YKL119C	VPH2	Integral membrane protein required for V-ATPase function
YKL125W	RRN3	Protein required for transcription of rDNA by RNA polymerase I
YKL138C-A	HSK3	Essential subunit of the Dam1 complex (aka DASH complex)
YKL166C	TPK3	cAMP-dependent protein kinase catalytic subunit
YKL184W	SPE1	Ornithine decarboxylase
YKL215C	OXP1	5-oxoprolinase
YKR079C	TRZ1	tRNA 3'-end processing endonuclease tRNase Z
YKR089C	TGL4	Multifunctional lipase/hydrolase/phospholipase
YLL013C	PUF3	Protein of the mitochondrial outer surface
YLL048C	YBT1	Transporter of the ATP-binding cassette (ABC) family
YLR084C	RAX2	N-glycosylated protein
YLR135W	SLX4	Endonuclease involved in processing DNA
YLR219W	MSC3	Protein of unknown function
YLR332W	MID2	O-glycosylated plasma membrane protein
YLR378C	SEC61	Conserved ER protein translocation channel
YLR451W	LEU3	Zinc-knuckle transcription factor, repressor and activator
YML023C	NSE5	Component of the SMC5-SMC6 complex
YML038C	YMD8	Putative nucleotide sugar transporter

YML055W	SPC2	Subunit of signal peptidase complex
YML056C	IMD4	Inosine monophosphate dehydrogenase
YML071C	COG8	Component of the conserved oligomeric Golgi complex
YML108W		Protein of unknown function
YML117W	NAB6	Putative RNA-binding protein
YMR033W	ARP9	Component of both the SWI/SNF and RSC chromatin remodeling complexes
YMR173W-A		Dubious open reading frame
YMR200W	ROT1	Molecular chaperone involved in protein folding in ER
YMR226C		NADP(+)-dependent serine dehydrogenase and carbonyl reductase
YNL024C-A	KSH1	Essential protein suggested to function early in the secretory pathway
YNL094W	APP1	Phosphatidate phosphatase, converts phosphatidate to diacylglycerol
YNL147W	LSM7	Lsm (Like Sm) protein
YNL149C	PGA2	Essential protein required for maturation of Gas1p and Pho8p
YNL189W	SRP1	Karyopherin alpha homolog
YNL199C	GCR2	Transcriptional activator of genes involved in glycolysis
YNL233W	BNI4	Targeting subunit for Glc7p protein phosphatase
YNL239W	LAP3	Cysteine aminopeptidase with homocysteine-thiolactonase activity
YNL286W	CUS2	Putative checkpoint factor in transcription
YNR017W	TIM23	Essential component of the TIM23 complex
YOL004W	SIN3	Component of both the Rpd3S and Rpd3L histone deacetylase complexes
YOL042W	NGL1	Putative endonuclease
YOL136C	PFK27	6-phosphofructo-2-kinase
YOR065W	CYT1	Cytochrome c1
YOR083W	WHI5	Repressor of G1 transcription
YOR113W	AZF1	Zinc-finger transcription factor
YOR142W	LSC1	Alpha subunit of succinyl-CoA ligase
YOR193W	PEX27	Peripheral peroxisomal membrane protein
YOR209C	NPT1	Nicotinate phosphoribosyltransferase
YOR232W	MGE1	Mitochondrial matrix cochaperone
YOR303W	CPA1	Small subunit of carbamoyl phosphate synthetase
YOR307C	SLY41	Protein involved in ER-to-Golgi transport
YOR312C	RPL20B	Ribosomal 60S subunit protein L20B
YOR335C	ALA1	Cytoplasmic and mitochondrial alanyl-tRNA synthetase
YOR342C		Protein of unknown function
YOR359W	VTS1	Flap-structured DNA-binding and RNA-binding protein
YOR369C	RPS12	Protein component of the small (40S) ribosomal subunit
YPL004C	LSP1	Primary component of eisosomes

YPL012W	RRP12	Protein required for export of the ribosomal subunits
YPL019C	VTC3	Subunit of vacuolar transporter chaperone (VTC) complex
YPL032C	SVL3	Protein of unknown function
YPL083C	SEN54	Subunit of the tRNA splicing endonuclease
YPL092W	SSU1	Plasma membrane sulfite pump involved in sulfite metabolism
YPL118W	MRP51	Mitochondrial ribosomal protein of the small subunit
YPL203W	TPK2	cAMP-dependent protein kinase catalytic subunit
YPL236C	ENV7	Vacuolar membrane protein kinase
YPR018W	RLF2	Largest subunit (p90) of the Chromatin Assembly Complex (CAF-1)
YPR072W	NOT5	Subunit of CCR4-NOT global transcriptional regulator
YPR124W	CTR1	High-affinity copper transporter of the plasma membrane
YPR179C	HDA3	Subunit of the HDA1 histone deacetylase complex

Table S9. Initiation at upstream UUG codons in *tcs2*Δ

Systematic Name	Standard Name	Description
YAL012W	CYS3	Cystathionine gamma-lyase
YAL026C-A		Dubious open reading frame
YAR033W	MST28	Putative integral membrane protein, involved in vesicle formation
YAR075W		Non-functional protein with homology IMP dehydrogenase
YBL029C-A		Protein of unknown function
YBL039C	URA7	Major CTP synthase isozyme (see also URA8)
YBL060W	YEL1	Guanine nucleotide exchange factor specific for Arf3p
YBL079W	NUP170	Subunit of the inner ring of the nuclear pore complex (NPC)
YBL101C	ECM21	Protein involved in regulating endocytosis of plasma membrane proteins
YBR011C	IPP1	Cytoplasmic inorganic pyrophosphatase (PPase)
YBR028C	YPK3	AGC kinase
YBR057C	MUM2	Protein essential for meiotic DNA replication and sporulation
YBR060C	ORC2	Subunit of the origin recognition complex (ORC)
YBR097W	VPS15	Serine/threonine protein kinase involved in vacuolar protein sorting
YBR121C	GRS1	Cytoplasmic and mitochondrial glycyl-tRNA synthase
YBR121C-A		Dubious open reading frame
YBR125C	PTC4	Cytoplasmic type 2C protein phosphatase (PP2C)
YBR129C	OPY1	Protein of unknown function
YBR146W	MRPS9	Mitochondrial ribosomal protein of the small subunit
YBR148W	YSW1	Protein required for normal prospore membrane formation
YBR166C	TYR1	Prephenate dehydrogenase involved in tyrosine biosynthesis
YBR167C	POP7	Subunit of both RNase MRP and nuclear RNase P

YBR168W	PEX32	Peroxisomal integral membrane protein
YBR172C	SMY2	GYF domain protein
YBR188C	NTC20	Member of the NineTeen Complex (NTC)
YBR193C	MED8	Subunit of the RNA polymerase II mediator complex
YBR204C	LDH1	Serine hydrolase
YBR212W	NGR1	RNA binding protein that negatively regulates growth rate
YBR221C	PDB1	E1 beta subunit of the pyruvate dehydrogenase (PDH) complex
YBR238C		Mitochondrial membrane protein
YBR275C	RIF1	Protein that binds to the Rap1p C-terminus
YCL014W	BUD3	Protein involved in bud-site selection
YCR091W	KIN82	Putative serine/threonine protein kinase
YCR094W	CDC50	Endosomal protein that interacts with phospholipid flippase Drs2p
YCR096C	HMRA2	Silenced copy of a2 at HMR
YDL003W	MCD1	Essential alpha-kleisin subunit of the cohesin complex
YDL017W	CDC7	DDK (Dbf4-dependent kinase) catalytic subunit
YDL025C	RTK1	Putative protein kinase, potentially phosphorylated by Cdc28p
YDL028C	MPS1	Dual-specificity kinase
YDL056W	MBP1	Transcription factor
YDL063C	SYO1	Transport adaptor or symportin
YDL129W		Protein of unknown function
YDL177C		Putative protein of unknown function
YDL208W	NHP2	Protein related to mammalian high mobility group (HMG) proteins
YDL211C		Protein of unknown function
YDL235C	YPD1	Phosphorelay intermediate protein
YDR037W	KRS1	Lysyl-tRNA synthetase
YDR043C	NRG1	Transcriptional repressor
YDR086C	SSS1	Subunit of the Sec61p translocation complex (Sec61p-Sss1p-Sbh1p)
YDR142C	PEX7	Peroxisomal signal receptor for peroxisomal matrix proteins
YDR144C	MKC7	GPI-anchored aspartyl protease
YDR155C	CPR1	Cytoplasmic peptidyl-prolyl cis-trans isomerase (cyclophilin)
YDR159W	SAC3	mRNA export factor
YDR172W	SUP35	Translation termination factor eRF3
YDR189W	SLY1	Hydrophilic protein involved in ER/Golgi vesicle trafficking
YDR267C	CIA1	Component of cytosolic iron-sulfur protein assembly (CIA) machinery
YDR292C	SRP101	Signal recognition particle (SRP) receptor alpha subunit
YDR319C	YFT2	Protein required for normal ER membrane biosynthesis
YDR347W	MRP1	Mitochondrial ribosomal protein of the small subunit
YDR357C	CNL1	Subunit of the BLOC-1 complex involved in endosomal maturation
YDR377W	ATP17	Subunit f of the F0 sector of mitochondrial F1F0 ATP synthase

YDR433W		Dubious open reading frame
YDR453C	TSA2	Stress inducible cytoplasmic thioredoxin peroxidase
YDR473C	PRP3	Splicing factor
YDR490C	PKH1	Serine/threonine protein kinase
YDR510W	SMT3	Ubiquitin-like protein of the SUMO family
YDR514C		Protein of unknown function that localizes to mitochondria
YDR530C	APA2	Diadenosine 5',5'''-P1,P4-tetraphosphate phosphorylase II
YDR541C		Putative dihydrokaempferol 4-reductase
YEL009C	GCN4	bZIP transcriptional activator of amino acid biosynthetic genes
YEL024W	RIP1	Ubiquinol-cytochrome-c reductase
YEL053C	MAK10	Non-catalytic subunit of N-terminal acetyltransferase of the NatC type
YEL063C	CAN1	Plasma membrane arginine permease
YEL072W	RMD6	Protein required for sporulation
YER014W	HEM14	Protoporphyrinogen oxidase
YER047C	SAP1	Putative ATPase of the AAA family
YER057C	HMF1	Member of the p14.5 protein family
YER059W	PCL6	Pho85p cyclin of the Pho80p subfamily
YER063W	THO1	Conserved nuclear RNA-binding protein
YER090W	TRP2	Anthranilate synthase
YER142C	MAG1	3-methyl-adenine DNA glycosylase
YER147C	SCC4	Subunit of cohesin loading factor (Scc2p-Scc4p)
YER156C		Putative protein of unknown function
YER169W	RPH1	JmjC domain-containing histone demethylase
YER190W	YRF1-2	Helicase encoded by the Y' element of subtelomeric regions
YFL007W	BLM10	Proteasome activator
YFL066C		Helicase-like protein encoded within the telomeric Y' element
YFR027W	ECO1	Acetyltransferase
YFR049W	YMR31	Mitochondrial ribosomal protein of the small subunit
YFR055W	IRC7	Beta-lyase involved in the production of thiols
YGL003C	CDH1	Activator of anaphase-promoting complex/cyclosome (APC/C)
YGL013C	PDR1	Transcription factor that regulates the pleiotropic drug response
YGL014W	PUF4	Member of the PUF protein family
YGL037C	PNC1	Nicotinamidase that converts nicotinamide to nicotinic acid
YGL039W		Oxidoreductase shown to reduce carbonyl compounds to chiral alcohols
YGL061C	DUO1	Essential subunit of the Dam1 complex (aka DASH complex)
YGL160W	AIM14	NADPH oxidase localized to the perinuclear ER
YGL179C	TOS3	Protein kinase
YGR026W		Putative protein of unknown function

YGR085C	RPL11B	Ribosomal 60S subunit protein L11B
YGR091W	PRP31	Splicing factor
YGR155W	CYS4	Cystathionine beta-synthase
YGR166W	TRS65	Component of transport protein particle (TRAPP) complex II
YGR177C	ATF2	Alcohol acetyltransferase
YGR188C	BUB1	Protein kinase involved in the cell cycle checkpoint into anaphase
YGR209C	TRX2	Cytoplasmic thioredoxin isoenzyme
YGR244C	LSC2	Beta subunit of succinyl-CoA ligase
YGR260W	TNA1	High affinity nicotinic acid plasma membrane permease
YGR264C	MES1	Methionyl-tRNA synthetase
YGR267C	FOL2	GTP-cyclohydrolase I
YGR281W	YOR1	Plasma membrane ATP-binding cassette (ABC) transporter
YHL002W	HSE1	Subunit of the endosomal Vps27p-Hse1p complex
YHL008C		Putative protein of unknown function
YHL010C	ETP1	Putative protein of unknown function required for growth on ethanol
YHL032C	GUT1	Glycerol kinase
YHR032W	ERC1	Member of the multi-drug and toxin extrusion (MATE) family
YHR036W	BRL1	Essential nuclear envelope integral membrane protein
YHR039C	MSC7	Protein of unknown function
YHR072W-A	NOP10	Subunit of box H/ACA snoRNP complex
YHR084W	STE12	Transcription factor that is activated by a MAPK signaling cascade
YHR087W	RTC3	Protein of unknown function involved in RNA metabolism
YHR121W	LSM12	Protein of unknown function that may function in RNA processing
YHR131C		Putative protein of unknown function
YHR158C	KEL1	Protein required for proper cell fusion and cell morphology
YHR170W	NMD3	Protein involved in nuclear export of the large ribosomal subunit
YHR172W	SPC97	Component of the microtubule-nucleating Tub4p (gamma-tubulin) complex
YHR186C	KOG1	Subunit of TORC1
YIL016W	SNL1	Ribosome-associated protein
YIL022W	TIM44	Essential component of the TIM23 complex
YIL103W	DPH1	Protein required for synthesis of diphthamide
YIL124W	AYR1	Bifunctional triacylglycerol lipase and 1-acyl DHAP reductase
YIL133C	RPL16A	Ribosomal 60S subunit protein L16A
YIR029W	DAL2	Allantoicase
YJL026W	RNR2	Ribonucleotide-diphosphate reductase (RNR), small subunit
YJL046W	AIM22	Putative lipoate-protein ligase
YJL130C	URA2	Bifunctional carbamoylphosphate synthetase/aspartate transcarbamylase

YJL146W	IDS2	Protein involved in modulation of Ime2p activity during meiosis
YJL164C	TPK1	cAMP-dependent protein kinase catalytic subunit
YJL168C	SET2	Histone methyltransferase with a role in transcriptional elongation
YJL183W	MNN11	Subunit of a Golgi mannosyltransferase complex
YJL196C	ELO1	Elongase I, medium-chain acyl elongase
YJL209W	CBP1	Mitochondrial protein, regulator of COB mRNA stability and translation
YJR034W	PET191	Protein required for assembly of cytochrome c oxidase
YJR054W	KCH1	Potassium transporter that mediates K+ influx
YJR131W	MNS1	Alpha-1,2-mannosidase
YKL004W	AUR1	Phosphatidylinositol:ceramide phosphoinositol transferase
YKL038W	RGT1	Glucose-responsive transcription factor
YKL076C	PSY1	Dubious open reading frame
YKL092C	BUD2	GTPase activating factor for Rsr1p/Bud1p
YKL098W	MTC2	Protein of unknown function
YKL119C	VPH2	Integral membrane protein required for V-ATPase function
YKL125W	RRN3	Protein required for transcription of rDNA by RNA polymerase I
YKL138C-A	HSK3	Essential subunit of the Dam1 complex (aka DASH complex)
YKL166C	TPK3	cAMP-dependent protein kinase catalytic subunit
YKL175W	ZRT3	Vacuolar membrane zinc transporter
YKL184W	SPE1	Ornithine decarboxylase
YKL196C	YKT6	Vesicle membrane protein (v-SNARE) with acyltransferase activity
YKL215C	OXP1	5-oxoprolinase
YKR027W	BCH2	Member of the ChAPs (Chs5p-Arf1p-binding proteins) family
YKR058W	GLG1	Glycogenin glucosyltransferase
YKR079C	TRZ1	tRNA 3'-end processing endonuclease tRNase Z
YKR089C	TGL4	Multifunctional lipase/hydrolase/phospholipase
YLL013C	PUF3	Protein of the mitochondrial outer surface
YLL048C	YBT1	Transporter of the ATP-binding cassette (ABC) family
YLR008C	PAM18	Subunit of the import motor (PAM complex)
YLR084C	RAX2	N-glycosylated protein
YLR135W	SLX4	Endonuclease involved in processing DNA
YLR202C		Dubious open reading frame
YLR219W	MSC3	Protein of unknown function
YLR224W		F-box protein and component of SCF ubiquitin ligase complexes
YLR241W		Putative protein of unknown function
YLR287C		Putative protein of unknown function
YLR326W		Putative protein of unknown function
YLR332W	MID2	O-glycosylated plasma membrane protein
YLR378C	SEC61	Conserved ER protein translocation channel

YLR451W	LEU3	Zinc-knuckle transcription factor, repressor and activator
YLR455W		Nuclear protein of unknown function
YML020W		Putative protein of unknown function
YML023C	NSE5	Component of the SMC5-SMC6 complex
YML032C	RAD52	Protein that stimulates strand exchange
YML037C		Putative protein of unknown function
YML038C	YMD8	Putative nucleotide sugar transporter
YML052W	SUR7	Plasma membrane protein of unknown function involved with endocytosis
YML055W	SPC2	Subunit of signal peptidase complex
YML056C	IMD4	Inosine monophosphate dehydrogenase
YML071C	COG8	Component of the conserved oligomeric Golgi complex
YML108W		Protein of unknown function
YML117W	NAB6	Putative RNA-binding protein
YMR033W	ARP9	Component of both the SWI/SNF and RSC chromatin remodeling complexes
YMR059W	SEN15	Subunit of the tRNA splicing endonuclease
YMR080C	NAM7	ATP-dependent RNA helicase of the SFI superfamily
YMR134W	ERG29	Protein of unknown function involved in ergosterol biosynthesis
YMR173W-A		Dubious open reading frame
YMR200W	ROT1	Molecular chaperone involved in protein folding in ER
YMR226C		NADP(+)-dependent serine dehydrogenase and carbonyl reductase
YMR234W	RNH1	Ribonuclease H1
YMR300C	ADE4	Phosphoribosylpyrophosphate amidotransferase (PRPPAT)
YNL018C		Putative protein of unknown function
YNL031C	HHT2	Histone H3
YNL094W	APP1	Phosphatidate phosphatase, converts phosphatidate to diacylglycerol
YNL139C	THO2	Subunit of the THO complex
YNL147W	LSM7	Lsm (Like Sm) protein
YNL149C	PGA2	Essential protein required for maturation of Gas1p and Pho8p
YNL152W	INN1	Essential protein that associates with contractile actomyosin ring
YNL189W	SRP1	Karyopherin alpha homolog
YNL199C	GCR2	Transcriptional activator of genes involved in glycolysis
YNL233W	BNI4	Targeting subunit for Glc7p protein phosphatase
YNL234W		Protein of unknown function with similarity to globins
YNL239W	LAP3	Cysteine aminopeptidase with homocysteine-thiolactonase activity
YNL255C	GIS2	Translational activator for mRNAs with internal ribosome entry sites
YNL280C	ERG24	C-14 sterol reductase
YNL286W	CUS2	Putative checkpoint factor in transcription

YNR017W	TIM23	Essential component of the TIM23 complex
YNR047W	FPK1	Ser/Thr protein kinase
YNR053C	NOG2	Putative GTPase
YOL004W	SIN3	Component of both the Rpd3S and Rpd3L histone deacetylase complexes
YOL028C	YAP7	Putative basic leucine zipper (bZIP) transcription factor
YOL042W	NGL1	Putative endonuclease
YOL061W	PRS5	5-phospho-ribosyl-1(alpha)-pyrophosphate synthetase
YOL063C	CRT10	Protein involved in transcriptional regulation of RNR2 and RNR3
YOL098C		Putative metalloprotease
YOL136C	PFK27	6-phosphofructo-2-kinase
YOL155C	HPF1	Haze-protective mannoprotein
YOR062C		Protein of unknown function
YOR065W	CYT1	Cytochrome c1
YOR083W	WHI5	Repressor of G1 transcription
YOR086C	TCB1	Lipid-binding ER protein involved in ER-plasma membrane tethering
YOR113W	AZF1	Zinc-finger transcription factor
YOR142W	LSC1	Alpha subunit of succinyl-CoA ligase
YOR188W	MSB1	Protein of unknown function
YOR193W	PEX27	Peripheral peroxisomal membrane protein
YOR209C	NPT1	Nicotinate phosphoribosyltransferase
YOR228C	MCP1	Mitochondrial protein of unknown function involved in lipid homeostas
YOR232W	MGE1	Mitochondrial matrix cochaperone
YOR260W	GCD1	Gamma subunit of the translation initiation factor eIF2B
YOR303W	CPA1	Small subunit of carbamoyl phosphate synthetase
YOR304W	ISW2	ATP-dependent DNA translocase involved in chromatin remodeling
YOR307C	SLY41	Protein involved in ER-to-Golgi transport
YOR335C	ALA1	Cytoplasmic and mitochondrial alanyl-tRNA synthetase
YOR347C	PYK2	Pyruvate kinase
YOR359W	VTS1	Flap-structured DNA-binding and RNA-binding protein
YOR369C	RPS12	Protein component of the small (40S) ribosomal subunit
YPL004C	LSP1	Primary component of eisosomes
YPL012W	RRP12	Protein required for export of the ribosomal subunits
YPL019C	VTC3	Subunit of vacuolar transporter chaperone (VTC) complex
YPL032C	SVL3	Protein of unknown function
YPL083C	SEN54	Subunit of the tRNA splicing endonuclease
YPL092W	SSU1	Plasma membrane sulfite pump involved in sulfite metabolism
YPL118W	MRP51	Mitochondrial ribosomal protein of the small subunit

YPL132W	COX11	Protein required for delivery of copper to Cox1p
YPL203W	TPK2	cAMP-dependent protein kinase catalytic subunit
YPL236C	ENV7	Vacuolar membrane protein kinase
YPR018W	RLF2	Largest subunit (p90) of the Chromatin Assembly Complex (CAF-1)
YPR070W	MED1	Subunit of the RNA polymerase II mediator complex
YPR072W	NOT5	Subunit of CCR4-NOT global transcriptional regulator
YPR124W	CTR1	High-affinity copper transporter of the plasma membrane
YPR131C	NAT3	Catalytic subunit of the NatB N-terminal acetyltransferase
YPR143W	RRP15	Nucleolar protein
YPR179C	HDA3	Subunit of the HDA1 histone deacetylase complex
YPR204W		DNA helicase encoded within the telomeric Y' element

Table S10. Initiation at upstream ACG in BY4742

Systematic Name	Standard Name	Description
YAL014C	SYN8	Endosomal SNARE related to mammalian syntaxin 8
YAR008W	SEN34	Subunit of the tRNA splicing endonuclease
YAR015W	ADE1	N-succinyl-5-aminoimidazole-4-carboxamide ribotide synthetase
YBL093C	ROX3	Subunit of the RNA polymerase II mediator complex
YBR007C	DSF2	Deletion suppressor of mpt5 mutation
YBR135W	CKS1	Cyclin-dependent protein kinase regulatory subunit and adaptor
YBR193C	MED8	Subunit of the RNA polymerase II mediator complex
YCR023C		Vacuolar membrane protein of unknown function
YCR027C	RHB1	Putative Rheb-related GTPase
YDL003W	MCD1	Essential alpha-kleisin subunit of the cohesin complex
YDL076C	RXT3	Component of the Rpd3L histone deacetylase complex
YDL077C	VAM6	Vacuolar protein involved in vacuolar membrane fusion tethering
YDL112W	TRM3	2'-O-ribose methyltransferase
YDL136W	RPL35B	Ribosomal 60S subunit protein L35B
YDL160C	DHH1	Cytoplasmic DExD/H-box helicase, stimulates mRNA decapping
YDL179W	PCL9	Cyclin
YDL208W	NHP2	Protein related to mammalian high mobility group (HMG) proteins
YDL224C	WHI4	Putative RNA binding protein
YDR133C		Dubious open reading frame
YDR154C		Dubious open reading frame
YDR210W		Predicted tail-anchored plasma membrane protein
YDR237W	MRPL7	Mitochondrial ribosomal protein of the large subunit
YDR281C	PHM6	Protein of unknown function
YDR284C	DPP1	Diacylglycerol pyrophosphate (DGPP) phosphatase

YDR309C	GIC2	Redundant rho-like GTPase Cdc42p effector
YDR545W	YRF1-1	Helicase encoded by the Y' element of subtelomeric regions
YER071C	TDA2	Protein of unknown function
YER072W	VTC1	Subunit of the vacuolar transporter chaperone (VTC) complex
YER088C-A		Dubious open reading frame
YER110C	KAP123	Karyopherin beta
YER118C	SHO1	Transmembrane osmosensor for filamentous growth and HOG pathways
YER159C	BUR6	Subunit of a heterodimeric NC2 transcription regulator complex
YGL071W	AFT1	Transcription factor involved in iron utilization and homeostasis
YGR017W		Putative protein of unknown function
YGR026W		Putative protein of unknown function
YGR137W		Dubious open reading frame
YGR156W	PTI1	Essential component of CPF (cleavage and polyadenylation factor)
YGR170W	PSD2	Phosphatidylserine decarboxylase of the Golgi and vacuolar membranes
YHL026C		Putative protein of unknown function
YHL034C	SBP1	Protein that binds eIF4G and has a role in repression of translation
YHR007C	ERG11	Lanosterol 14-alpha-demethylase
YHR162W	MPC2	Highly conserved subunit of the mitochondrial pyruvate carrier
YIL087C	AIM19	Putative protein of unknown function
YIL133C	RPL16A	Ribosomal 60S subunit protein L16A
YIL153W	RRD1	Peptidyl-prolyl cis/trans-isomerase
YIL177C		Putative Y' element ATP-dependent helicase
YJL016W		Putative protein of unknown function
YJL046W	AIM22	Putative lipoate-protein ligase
YJL154C	VPS35	Endosomal subunit of membrane-associated retromer complex
YJL166W	QCR8	Subunit 8 of ubiquinol cytochrome-c reductase (Complex III)
YJL225C		Putative Y' element ATP-dependent helicase
YKL032C	IXR1	Transcriptional repressor that regulates hypoxic genes during normoxia
YKL138C-A	HSK3	Essential subunit of the Dam1 complex (aka DASH complex)
YKL148C	SDH1	Flavoprotein subunit of succinate dehydrogenase
YKL183W	LOT5	Protein of unknown function
YKL198C	PTK1	Putative serine/threonine protein kinase
YKR001C	VPS1	Dynamin-like GTPase required for vacuolar sorting
YKR079C	TRZ1	tRNA 3'-end processing endonuclease tRNase Z
YLL021W	SPA2	Component of the polarisome

YLL048C	YBT1	Transporter of the ATP-binding cassette (ABC) family
YLR026C	SED5	cis-Golgi t-SNARE syntaxin
YLR042C		Protein of unknown function
YLR084C	RAX2	N-glycosylated protein
YLR221C	RSA3	Protein with a likely role in ribosomal maturation
YLR254C	NDL1	Homolog of nuclear distribution factor NudE
YLR340W	RPP0	Conserved ribosomal protein P0 of the ribosomal stalk
YLR467W	YRF1-5	Helicase encoded by the Y' element of subtelomeric regions
YML020W		Putative protein of unknown function
YML055W	SPC2	Subunit of signal peptidase complex
YML108W		Protein of unknown function
YML116W-A		Putative protein of unknown function
YML133C		Putative Y' element ATP-dependent helicase
YMR043W	MCM1	Transcription factor
YMR102C		Protein of unknown function
YMR176W	ECM5	Subunit of the Snt2C complex
YMR304C-A		Dubious open reading frame
YNL021W	HDA1	Putative catalytic subunit of a class II histone deacetylase complex
YNL156C	NSG2	Protein involved in regulation of sterol biosynthesis
YNL199C	GCR2	Transcriptional activator of genes involved in glycolysis
YNL233W	BNI4	Targeting subunit for Glc7p protein phosphatase
YNL239W	LAP3	Cysteine aminopeptidase with homocysteine-thiolactonase activity
YNL282W	POP3	Subunit of both RNase MRP and nuclear RNase P
YNR010W	CSE2	Subunit of the RNA polymerase II mediator complex
YNR044W	AGA1	Anchorage subunit of a-agglutinin of a-cells
YOL113W	SKM1	Member of the PAK family of serine/threonine protein kinases
YOR014W	RTS1	B-type regulatory subunit of protein phosphatase 2A (PP2A)
YOR307C	SLY41	Protein involved in ER-to-Golgi transport
YOR335C	ALA1	Cytoplasmic and mitochondrial alanyl-tRNA synthetase
YOR396W	YRF1-8	One of several telomeric Y' element-encoded DNA helicases
YPL070W	MUK1	Guanine nucleotide exchange factor (GEF)
YPL082C	MOT1	Essential protein involved in regulation of transcription
YPL092W	SSU1	Plasma membrane sulfite pump involved in sulfite metabolism
YPL144W	POC4	Component of a heterodimeric Poc4p-Irc25p chaperone
YPL183W-A	RTC6	Protein involved in translation
YPL221W	FLC1	Putative FAD transporter
YPR073C	LTP1	Protein phosphotyrosine phosphatase of unknown cellular role
YPR128C	ANT1	Peroxisomal adenine nucleotide transporter

YPR170W-A		Dubious open reading frame

Table S11 Initiation at upstream ACG in *tcs2*Δ

Systematic Name	Standard Name	Description
YAL014C	SYN8	Endosomal SNARE related to mammalian syntaxin 8
YAL039C	CYC3	Cytochrome c heme lyase (holocytochrome c synthase)
YAR008W	SEN34	Subunit of the tRNA splicing endonuclease
YAR015W	ADE1	N-succinyl-5-aminoimidazole-4-carboxamide ribotide synthetase
YAR075W		Non-functional protein with homology IMP dehydrogenase
YBL084C	CDC27	Subunit of the Anaphase-Promoting Complex/Cyclosome (APC/C)
YBL093C	ROX3	Subunit of the RNA polymerase II mediator complex
YBL111C		Helicase-like protein encoded within the telomeric Y' element
YBR007C	DSF2	Deletion suppressor of mpt5 mutation
YBR065C	ECM2	Pre-mRNA splicing factor
YBR112C	CYC8	General transcriptional co-repressor
YBR131W	CCZ1	Protein involved in vacuolar assembly
YBR135W	CKS1	Cyclin-dependent protein kinase regulatory subunit and adaptor
YBR148W	YSW1	Protein required for normal prospore membrane formation
YBR188C	NTC20	Member of the NineTeen Complex (NTC)
YBR193C	MED8	Subunit of the RNA polymerase II mediator complex
YBR206W		Dubious open reading frame
YBR257W	POP4	Subunit of both RNase MRP and nuclear RNase P
YBR262C	MIC12	Component of the MICOS complex
YCR023C		Vacuolar membrane protein of unknown function
YCR027C	RHB1	Putative Rheb-related GTPase
YDL003W	MCD1	Essential alpha-kleisin subunit of the cohesin complex
YDL013W	SLX5	Subunit of the Slx5-Slx8 SUMO-targeted ubiquitin ligase complex
YDL076C	RXT3	Component of the Rpd3L histone deacetylase complex
YDL077C	VAM6	Vacuolar protein involved in vacuolar membrane fusion tethering
YDL095W	PMT1	Protein O-mannosyltransferase of the ER membrane
YDL097C	RPN6	Essential, non-ATPase regulatory subunit of the 26S proteasome lid
YDL112W	TRM3	2'-O-ribose methyltransferase
YDL136W	RPL35B	Ribosomal 60S subunit protein L35B
YDL160C	DHH1	Cytoplasmic DExD/H-box helicase, stimulates mRNA decapping
YDL179W	PCL9	Cyclin
YDL208W	NHP2	Protein related to mammalian high mobility group (HMG) proteins
YDL224C	WHI4	Putative RNA binding protein

YDL235C	YPD1	Phosphorelay intermediate protein
YDR118W	APC4	Subunit of the Anaphase-Promoting Complex/Cyclosome (APC/C)
YDR133C		Dubious open reading frame
YDR144C	MKC7	GPI-anchored aspartyl protease
YDR154C		Dubious open reading frame
YDR192C	NUP42	FG-nucleoporin component of central core of the nuclear pore complex
YDR210W		Predicted tail-anchored plasma membrane protein
YDR237W	MRPL7	Mitochondrial ribosomal protein of the large subunit
YDR284C	DPP1	Diacylglycerol pyrophosphate (DGPP) phosphatase
YDR309C	GIC2	Redundant rho-like GTPase Cdc42p effector
YDR335W	MSN5	Karyopherin
YDR421W	ARO80	Zinc finger transcriptional activator of the Zn2Cys6 family
YDR490C	PKH1	Serine/threonine protein kinase
YDR528W	HLR1	Protein involved in regulation of cell wall composition and integrity
YDR545W	YRF1-1	Helicase encoded by the Y' element of subtelomeric regions
YEL053C	MAK10	Non-catalytic subunit of N-terminal acetyltransferase of the NatC type
YEL075C		Putative protein of unknown function
YER014W	HEM14	Protoporphyrinogen oxidase
YER072W	VTC1	Subunit of the vacuolar transporter chaperone (VTC) complex
YER088C-A		Dubious open reading frame
YER110C	KAP123	Karyopherin beta
YER118C	SHO1	Transmembrane osmosensor for filamentous growth and HOG pathways
YER159C	BUR6	Subunit of a heterodimeric NC2 transcription regulator complex
YER190W	YRF1-2	Helicase encoded by the Y' element of subtelomeric regions
YFL064C		Putative protein of unknown function
YGL071W	AFT1	Transcription factor involved in iron utilization and homeostasis
YGL073W	HSF1	Trimeric heat shock transcription factor
YGL137W	SEC27	Essential beta'-coat protein of the COPI coatomer
YGR017W		Putative protein of unknown function
YGR026W		Putative protein of unknown function
YGR137W		Dubious open reading frame
YGR156W	PTI1	Essential component of CPF (cleavage and polyadenylation factor)
YGR170W	PSD2	Phosphatidylserine decarboxylase of the Golgi and vacuolar membranes
YGR209C	TRX2	Cytoplasmic thioredoxin isoenzyme
YGR281W	YOR1	Plasma membrane ATP-binding cassette (ABC) transporter

YHL026C		Putative protein of unknown function
YHL034C	SBP1	Protein that binds eIF4G and has a role in repression of translation
YHL039W	EFM1	Lysine methyltransferase
YHL049C		Putative protein of unknown function
YHR007C	ERG11	Lanosterol 14-alpha-demethylase
YHR036W	BRL1	Essential nuclear envelope integral membrane protein
YHR102W	KIC1	Protein kinase of the PAK/Ste20 family, required for cell integrity
YHR162W	MPC2	Highly conserved subunit of the mitochondrial pyruvate carrier
YHR218W		Helicase-like protein encoded within the telomeric Y' element
YIL001W		Putative protein of unknown function
YIL031W	ULP2	Peptidase that deconjugates Smt3/SUMO-1 peptides from proteins
YIL055C		Putative protein of unknown function
YIL087C	AIM19	Putative protein of unknown function
YIL101C	XBP1	Transcriptional repressor
YIL133C	RPL16A	Ribosomal 60S subunit protein L16A
YIL134W	FLX1	Protein required for transport of flavin adenine dinucleotide (FAD)
YIL153W	RRD1	Peptidyl-prolyl cis/trans-isomerase
YIL177C		Putative Y' element ATP-dependent helicase
YJL001W	PRE3	Beta 1 subunit of the 20S proteasome
YJL016W		Putative protein of unknown function
YJL046W	AIM22	Putative lipoate-protein ligase
YJL078C	PRY3	Cell wall-associated protein involved in export of acetylated sterols
YJL083W	TAX4	EH domain-containing protein
YJL110C	GZF3	GATA zinc finger protein
YJL154C	VPS35	Endosomal subunit of membrane-associated retromer complex
YJL166W	QCR8	Subunit 8 of ubiquinol cytochrome-c reductase (Complex III)
YJL225C		Putative Y' element ATP-dependent helicase
YKL015W	PUT3	Transcriptional activator
YKL032C	IXR1	Transcriptional repressor that regulates hypoxic genes during normoxia
YKL055C	OAR1	Mitochondrial 3-oxoacyl-[acyl-carrier-protein] reductase
YKL113C	RAD27	5' to 3' exonuclease, 5' flap endonuclease
YKL138C	MRPL31	Mitochondrial ribosomal protein of the large subunit
YKL138C-A	HSK3	Essential subunit of the Dam1 complex (aka DASH complex)
YKL148C	SDH1	Flavoprotein subunit of succinate dehydrogenase
YKL175W	ZRT3	Vacuolar membrane zinc transporter
YKL183W	LOT5	Protein of unknown function
YKR079C	TRZ1	tRNA 3'-end processing endonuclease tRNase Z
YLL013C	PUF3	Protein of the mitochondrial outer surface

YLL021W	SPA2	Component of the polarisome
YLL048C	YBT1	Transporter of the ATP-binding cassette (ABC) family
YLR008C	PAM18	Subunit of the import motor (PAM complex)
YLR026C	SED5	cis-Golgi t-SNARE syntaxin
YLR042C		Protein of unknown function
YLR079W	SIC1	Cyclin-dependent kinase inhibitor (CKI)
YLR084C	RAX2	N-glycosylated protein
YLR164W	SHH4	Mitochondrial inner membrane protein of unknown function
YLR178C	TFS1	Protein that interacts with and inhibits carboxypeptidase Y and Ira2p
YLR221C	RSA3	Protein with a likely role in ribosomal maturation
YLR254C	NDL1	Homolog of nuclear distribution factor NudE
YLR325C	RPL38	Ribosomal 60S subunit protein L38
YLR382C	NAM2	Mitochondrial leucyl-tRNA synthetase
YLR438C-A	LSM3	Lsm (Like Sm) protein
YLR462W		Putative protein of unknown function with similarity to helicases
YLR467W	YRF1-5	Helicase encoded by the Y' element of subtelomeric regions
YML020W		Putative protein of unknown function
YML055W	SPC2	Subunit of signal peptidase complex
YML108W		Protein of unknown function
YML116W-A		Putative protein of unknown function
YML133C		Putative Y' element ATP-dependent helicase
YMR043W	MCM1	Transcription factor
YMR059W	SEN15	Subunit of the tRNA splicing endonuclease
YMR080C	NAM7	ATP-dependent RNA helicase of the SFI superfamily
YMR102C		Protein of unknown function
YMR173W	DDR48	DNA damage-responsive protein
YMR176W	ECM5	Subunit of the Snt2C complex
YMR234W	RNH1	Ribonuclease H1
YMR238W	DFG5	Putative mannosidase
YMR281W	GPI12	ER membrane protein involved in the second step of GPI anchor assembly
YMR304C-A		Dubious open reading frame
YNL021W	HDA1	Putative catalytic subunit of a class II histone deacetylase complex
YNL044W	YIP3	Protein localized to COPII vesicles
YNL152W	INN1	Essential protein that associates with contractile actomyosin ring
YNL156C	NSG2	Protein involved in regulation of sterol biosynthesis
YNL199C	GCR2	Transcriptional activator of genes involved in glycolysis
YNL233W	BNI4	Targeting subunit for Glc7p protein phosphatase

YNL239W	LAP3	Cysteine aminopeptidase with homocysteine-thiolactonase activity
YNL255C	GIS2	Translational activator for mRNAs with internal ribosome entry sites
YNL282W	POP3	Subunit of both RNase MRP and nuclear RNase P
YNR010W	CSE2	Subunit of the RNA polymerase II mediator complex
YNR044W	AGA1	Anchorage subunit of a-agglutinin of a-cells
YOL077C	BRX1	Nucleolar protein
YOL098C		Putative metalloprotease
YOL113W	SKM1	Member of the PAK family of serine/threonine protein kinases
YOR014W	RTS1	B-type regulatory subunit of protein phosphatase 2A (PP2A)
YOR030W	DFG16	Probable multiple transmembrane protein
YOR191W	ULS1	Swi2/Snf2-related translocase, SUMO-Targeted Ubiquitin Ligase (STUbL)
YOR208W	PTP2	Phosphotyrosine-specific protein phosphatase
YOR236W	DFR1	Dihydrofolate reductase involved in tetrahydrofolate biosynthesis
YOR307C	SLY41	Protein involved in ER-to-Golgi transport
YOR335C	ALA1	Cytoplasmic and mitochondrial alanyl-tRNA synthetase
YOR396W	YRF1-8	One of several telomeric Y' element-encoded DNA helicases
YPL045W	VPS16	Subunit of the HOPS and the CORVET complexes
YPL082C	MOT1	Essential protein involved in regulation of transcription
YPL092W	SSU1	Plasma membrane sulfite pump involved in sulfite metabolism
YPL144W	POC4	Component of a heterodimeric Poc4p-Irc25p chaperone
YPR010C-A		Putative protein of unknown function
YPR073C	LTP1	Protein phosphotyrosine phosphatase of unknown cellular role
YPR128C	ANT1	Peroxisomal adenine nucleotide transporter
YPR144C	NOC4	Nucleolar protein
YPR170W-A		Dubious open reading frame
YPR191W	QCR2	Subunit 2 of ubiquinol cytochrome-c reductase (Complex III)

Table S12. Initiation at upstream GUG in BY4742

Systematic Name	Standard Name	Description
YBL029C-A		Protein of unknown function
YBL039C-A		Dubious open reading frame
YBL068W	PRS4	5-phospho-ribosyl-1(alpha)-pyrophosphate synthetase, synthesizes PRPP
YBL093C	ROX3	Subunit of the RNA polymerase II mediator complex
YBR071W		Protein of unknown function found in the cytoplasm and bud neck
YBR102C	EXO84	Exocyst subunit with dual roles in exocytosis and

		spliceosome assembly
YBR168W	PEX32	Peroxisomal integral membrane protein
YBR211C	AME1	Essential kinetochore protein associated with microtubules and SPBs
YCL037C	SRO9	Cytoplasmic RNA-binding protein
YCR019W	MAK32	Protein necessary for stability of L-A dsRNA-containing particles
YDR016C	DAD1	Essential subunit of the Dam1 complex (aka DASH complex)
YDR133C		Dubious open reading frame
YDR142C	PEX7	Peroxisomal signal receptor for peroxisomal matrix proteins
YDR154C		Dubious open reading frame
YDR157W		Dubious open reading frame
YDR170C	SEC7	Guanine nucleotide exchange factor (GEF) for ADP ribosylation factors
YDR379W	RGA2	GTPase-activating protein for polarity-establishment protein Cdc42p
YDR399W	HPT1	Dimeric hypoxanthine-guanine phosphoribosyltransferase
YDR441C	APT2	Potential adenine phosphoribosyltransferase
YDR460W	TFB3	Subunit of TFIIH and nucleotide excision repair factor 3 complexes
YDR531W	CAB1	Pantothenate kinase, ATP:D-pantothenate 4'-phosphotransferase
YER071C	TDA2	Protein of unknown function
YGL006W	PMC1	Vacuolar Ca2+ ATPase involved in depleting cytosol of Ca2+ ions
YGR017W		Putative protein of unknown function
YGR135W	PRE9	Alpha 3 subunit of the 20S proteasome
YGR137W		Dubious open reading frame
YGR157W	CHO2	Phosphatidylethanolamine methyltransferase (PEMT)
YGR267C	FOL2	GTP-cyclohydrolase I
YHL004W	MRP4	Mitochondrial ribosomal protein of the small subunit
YHR115C	DMA1	Ubiquitin-protein ligase (E3)
YHR135C	YCK1	Palmitoylated plasma membrane-bound casein kinase I (CK1) isoform
YHR168W	MTG2	Putative GTPase
YHR216W	IMD2	Inosine monophosphate dehydrogenase
YIL016W	SNL1	Ribosome-associated protein
YIL030C	SSM4	Ubiquitin-protein ligase involved in ER-associated protein degradation
YJL138C	TIF2	Translation initiation factor eIF4A

YJL154C	VPS35	Endosomal subunit of membrane-associated retromer complex
YJR007W	SUI2	Alpha subunit of the translation initiation factor eIF2
YKL019W	RAM2	Alpha subunit of farnesyltransferase and geranylgeranyltransferase-I
YLL040C	VPS13	Protein involved in prospore membrane morphogenesis
YLR220W	CCC1	Putative vacuolar Fe2+/Mn2+ transporter
YLR224W		F-box protein and component of SCF ubiquitin ligase complexes
YLR284C	ECI1	Peroxisomal delta3,delta2-enoyl-CoA isomerase
YLR432W	IMD3	Inosine monophosphate dehydrogenase
YML038C	YMD8	Putative nucleotide sugar transporter
YML080W	DUS1	Dihydrouridine synthase
YMR043W	MCM1	Transcription factor
YMR176W	ECM5	Subunit of the Snt2C complex
YMR308C	PSE1	Karyopherin/importin that interacts with the nuclear pore complex
YOL032W	OPI10	Protein with a possible role in phospholipid biosynthesis
YOL158C	ENB1	Endosomal ferric enterobactin transporter
YOR157C	PUP1	Beta 2 subunit of the 20S proteasome
YOR162C	YRR1	Zn2-Cys6 zinc-finger transcription factor
YOR211C	MGM1	Mitochondrial GTPase, present in complex with Ugo1p and Fzo1p
YOR279C	RFM1	Component of the Sum1p-Rfm1p-Hst1p complex
YOR294W	RRS1	Essential protein that binds ribosomal protein L11
YOR307C	SLY41	Protein involved in ER-to-Golgi transport
YPL024W	RMI1	Subunit of the RecQ (Sgs1p) - Topo III (Top3p) complex
YPL144W	POC4	Component of a heterodimeric Poc4p-Irc25p chaperone
YPL226W	NEW1	ATP binding cassette protein
YPR085C	ASA1	Subunit of the ASTRA complex, involved in chromatin remodeling
YPR097W		Protein that contains a PX domain and binds phosphoinositides

Table S13. Initiation at upstream GUG codons in *tcs2*Δ

Systematic Name	Standard Name	Description
YAL026C-A		Dubious open reading frame
YAR075W		Non-functional protein with homology IMP dehydrogenase

YBL029C-A		Protein of unknown function
YBL039C-A		Dubious open reading frame
YBL068W	PRS4	5-phospho-ribosyl-1(alpha)-pyrophosphate synthetase, synthesizes PRPP
YBL093C	ROX3	Subunit of the RNA polymerase II mediator complex
YBR054W	YRO2	Protein of unknown function with similarity to archaeal rhodopsins
YBR071W		Protein of unknown function found in the cytoplasm and bud neck
YBR102C	EXO84	Exocyst subunit with dual roles in exocytosis and spliceosome assembly
YBR168W	PEX32	Peroxisomal integral membrane protein
YBR206W		Dubious open reading frame
YBR211C	AME1	Essential kinetochore protein associated with microtubules and SPBs
YBR238C		Mitochondrial membrane protein
YCL024W	KCC4	Protein kinase of the bud neck involved in the septin checkpoint
YCL037C	SRO9	Cytoplasmic RNA-binding protein
YCR019W	MAK32	Protein necessary for stability of L-A dsRNA-containing particles
YCR091W	KIN82	Putative serine/threonine protein kinase
YDL056W	MBP1	Transcription factor
YDL127W	PCL2	Cyclin, interacts with cyclin-dependent kinase Pho85p
YDL197C	ASF2	Anti-silencing protein
YDL223C	HBT1	Shmoo tip protein, substrate of Hub1p ubiquitin-like protein
YDR016C	DAD1	Essential subunit of the Dam1 complex (aka DASH complex)
YDR047W	HEM12	Uroporphyrinogen decarboxylase
YDR075W	PPH3	Catalytic subunit of protein phosphatase PP4 complex
YDR078C	SHU2	Component of the Shu complex, which promotes error-free DNA repair
YDR133C		Dubious open reading frame
YDR142C	PEX7	Peroxisomal signal receptor for peroxisomal matrix proteins
YDR154C		Dubious open reading frame
YDR157W		Dubious open reading frame
YDR170C	SEC7	Guanine nucleotide exchange factor (GEF) for ADP ribosylation factors
YDR307W	PMT7	Putative protein mannosyltransferase similar to Pmt1p
YDR362C	TFC6	Subunit of RNA polymerase III transcription initiation factor complex
YDR379W	RGA2	GTPase-activating protein for polarity-establishment protein Cdc42p
YDR399W	HPT1	Dimeric hypoxanthine-guanine phosphoribosyltransferase
YDR407C	TRS120	Component of transport protein particle (TRAPP) complex II
YDR439W	LRS4	Nucleolar protein that forms a complex with Csm1p
YDR441C	APT2	Potential adenine phosphoribosyltransferase

YDR460W	TFB3	Subunit of TFIIH and nucleotide excision repair factor 3 complexes
YDR490C	PKH1	Serine/threonine protein kinase
YDR497C	ITR1	Myo-inositol transporter
YDR516C	EMI2	Non-essential protein of unknown function
YDR528W	HLR1	Protein involved in regulation of cell wall composition and integrity
YDR531W	CAB1	Pantothenate kinase, ATP:D-pantothenate 4'-phosphotransferase
YEL071W	DLD3	D-lactate dehydrogenase
YER014W	HEM14	Protoporphyrinogen oxidase
YER019C-A	SBH2	Ssh1p-Sss1p-Sbh2p complex component
YER062C	GPP2	DL-glycerol-3-phosphate phosphatase involved in glycerol biosynthesis
YER119C	AVT6	Vacuolar aspartate and glutamate exporter
YER156C		Putative protein of unknown function
YFR055W	IRC7	Beta-lyase involved in the production of thiols
YGL006W	PMC1	Vacuolar Ca2+ ATPase involved in depleting cytosol of Ca2+ ions
YGL073W	HSF1	Trimeric heat shock transcription factor
YGL157W	ARI1	NADPH-dependent aldehyde reductase
YGL160W	AIM14	NADPH oxidase localized to the perinuclear ER
YGR017W		Putative protein of unknown function
YGR135W	PRE9	Alpha 3 subunit of the 20S proteasome
YGR137W		Dubious open reading frame
YGR157W	CHO2	Phosphatidylethanolamine methyltransferase (PEMT)
YGR231C	PHB2	Subunit of the prohibitin complex (Phb1p-Phb2p)
YGR267C	FOL2	GTP-cyclohydrolase I
YHL004W	MRP4	Mitochondrial ribosomal protein of the small subunit
YHR078W		High osmolarity-regulated gene of unknown function
YHR115C	DMA1	Ubiquitin-protein ligase (E3)
YHR135C	YCK1	Palmitoylated plasma membrane-bound casein kinase I (CK1) isoform
YHR168W	MTG2	Putative GTPase
YHR210C		Putative aldose 1-epimerase superfamily protein
YHR216W	IMD2	Inosine monophosphate dehydrogenase
YHR218W		Helicase-like protein encoded within the telomeric Y' element
YIL016W	SNL1	Ribosome-associated protein
YIL030C	SSM4	Ubiquitin-protein ligase involved in ER-associated protein degradation
YIL042C	PKP1	Mitochondrial protein kinase
YIL144W	NDC80	Component of the kinetochore-associated Ndc80 complex
YIR021W	MRS1	Splicing protein
YJL081C	ARP4	Nuclear actin-related protein involved in chromatin remodeling
YJL110C	GZF3	GATA zinc finger protein

YJL138C	TIF2	Translation initiation factor eIF4A
YJL142C	IRC9	Dubious open reading frame
YJL154C	VPS35	Endosomal subunit of membrane-associated retromer complex
YJL183W	MNN11	Subunit of a Golgi mannosyltransferase complex
YJR007W	SUI2	Alpha subunit of the translation initiation factor eIF2
YJR030C		Putative protein of unknown function
YKL015W	PUT3	Transcriptional activator
YKL019W	RAM2	Alpha subunit of farnesyltransferase and geranylgeranyltransferase-I
YKL134C	1-Oct	Mitochondrial intermediate peptidase
YKL175W	ZRT3	Vacuolar membrane zinc transporter
YKR027W	BCH2	Member of the ChAPs (Chs5p-Arf1p-binding proteins) family
YKR058W	GLG1	Glycogenin glucosyltransferase
YLL040C	VPS13	Protein involved in prospore membrane morphogenesis
YLL062C	MHT1	S-methylmethionine-homocysteine methyltransferase
YLR164W	SHH4	Mitochondrial inner membrane protein of unknown function
YLR220W	CCC1	Putative vacuolar Fe2+/Mn2+ transporter
YLR224W		F-box protein and component of SCF ubiquitin ligase complexes
YLR284C	ECI1	Peroxisomal delta3,delta2-enoyl-CoA isomerase
YLR352W		Putative protein of unknown function with similarity to F-box proteins
YLR382C	NAM2	Mitochondrial leucyl-tRNA synthetase
YLR432W	IMD3	Inosine monophosphate dehydrogenase
YLR455W		Nuclear protein of unknown function
YML012W	ERV25	Member of the p24 family involved in ER to Golgi transport
YML020W		Putative protein of unknown function
YML032C	RAD52	Protein that stimulates strand exchange
YML038C	YMD8	Putative nucleotide sugar transporter
YML080W	DUS1	Dihydrouridine synthase
YMR043W	MCM1	Transcription factor
YMR134W	ERG29	Protein of unknown function involved in ergosterol biosynthesis
YMR176W	ECM5	Subunit of the Snt2C complex
YMR234W	RNH1	Ribonuclease H1
YMR281W	GPI12	ER membrane protein involved in the second step of GPI anchor assembly
YMR308C	PSE1	Karyopherin/importin that interacts with the nuclear pore complex
YNL031C	HHT2	Histone H3
YNL139C	THO2	Subunit of the THO complex
YNL272C	SEC2	Guanyl-nucleotide exchange factor for the small G-protein Sec4p
YNL330C	RPD3	Histone deacetylase, component of both the Rpd3S and Rpd3L complexes
YOL032W	OPI10	Protein with a possible role in phospholipid biosynthesis

YOL098C		Putative metalloprotease
YOL139C	CDC33	mRNA cap binding protein and translation initiation factor eIF4E
YOL158C	ENB1	Endosomal ferric enterobactin transporter
YOR021C	SFM1	SPOUT methyltransferase
YOR062C		Protein of unknown function
YOR157C	PUP1	Beta 2 subunit of the 20S proteasome
YOR162C	YRR1	Zn2-Cys6 zinc-finger transcription factor
YOR180C	DCI1	Peroxisomal protein
YOR208W	PTP2	Phosphotyrosine-specific protein phosphatase
YOR211C	MGM1	Mitochondrial GTPase, present in complex with Ugo1p and Fzo1p
YOR231W	MKK1	MAPKK involved in the protein kinase C signaling pathway
YOR279C	RFM1	Component of the Sum1p-Rfm1p-Hst1p complex
YOR307C	SLY41	Protein involved in ER-to-Golgi transport
YPL066W	RGL1	Regulator of Rho1p signaling, cofactor of Tus1p
YPL085W	SEC16	COPII vesicle coat protein required for ER transport vesicle budding
YPL144W	POC4	Component of a heterodimeric Poc4p-Irc25p chaperone
YPL150W		Protein kinase of unknown cellular role
YPL153C	RAD53	DNA damage response protein kinase
YPL212C	PUS1	tRNA:pseudouridine synthase
YPR085C	ASA1	Subunit of the ASTRA complex, involved in chromatin remodeling
YPR097W		Protein that contains a PX domain and binds phosphoinositides

Table S14. Genes with increased RPFs in both *tcs2*Δ and *ncs6*Δ

Systematic Name	Standard Name	Description
YAL061W	BDH2	Putative medium-chain alcohol dehydrogenase with similarity to BDH1
YDL174C	DLD1	D-lactate dehydrogenase
YDR216W	ADR1	Carbon source-responsive zinc-finger transcription factor
YEL009C	GCN4	bZIP transcriptional activator of amino acid biosynthetic genes
YEL011W	GLC3	Glycogen branching enzyme, involved in glycogen accumulation
YFL014W	HSP12	Plasma membrane protein involved in maintaining membrane organization
YGR043C	NQM1	Transaldolase of unknown function
YGR088W	CTT1	Cytosolic catalase T
YGR248W	SOL4	6-phosphogluconolactonase
YIR031C	DAL7	Malate synthase
YIR039C	YPS6	Putative GPI-anchored aspartic protease
YJR109C	CPA2	Large subunit of carbamoyl phosphate synthetase

YKL161C	KDX1	Protein kinase
YKR039W	GAP1	General amino acid permease
YML128C	MSC1	Protein of unknown function
YMR062C	ARG7	Mitochondrial ornithine acetyltransferase
YNL036W	NCE103	Carbonic anhydrase
YNR001C	CIT1	Citrate synthase
YOR374W	ALD4	Mitochondrial aldehyde dehydrogenase

Table S15. Genes with decreased RPFs in both *tcs2*Δ and *ncs6*Δ

Systematic Name	Standard Name	Description
YBR093C	PHO5	Repressible acid phosphatase
YER011W	TIR1	Cell wall mannoprotein
YFL034C-A	RPL22B	Ribosomal 60S subunit protein L22B
YGL089C	MF(ALPHA)2	Mating pheromone alpha-factor, made by alpha cells
YGL255W	ZRT1	High-affinity zinc transporter of the plasma membrane
YHR215W	PHO12	One of three repressible acid phosphatases
YIL011W	TIR3	Cell wall mannoprotein
YJR047C	ANB1	Translation elongation factor eIF-5A
YLL052C	AQY2	Water channel that mediates water transport across cell membranes
YML058W-A	HUG1	Protein involved in the Mec1p-mediated checkpoint pathway
YMR006C	PLB2	Phospholipase B (lysophospholipase) involved in lipid metabolism
YPL081W	RPS9A	Protein component of the small (40S) ribosomal subunit

Table S16. Gene ontology enrichment for genes using only AGA/AGG codons to encode arginine.

GO Term	P-Value	Number of Genes
hydrogen ion transmembrane transport	8.99E-11	21
respiratory electron transport chain	1.02E-10	16
oxidative phosphorylation	2.02E-10	16
electron transport chain	2.02E-10	16
proton transport	3.36E-10	22
hydrogen transport	4.77E-10	22
ATP synthesis coupled electron transport	6.24E-10	15
mitochondrial ATP synthesis coupled electron transport	6.24E-10	15
monovalent inorganic cation transport	1.56E-07	22

mitochondrial electron transport, cytochrome c to oxygen	7.38E-07	9
cell redox homeostasis	1.88E-04	11
mitochondrial electron transport, ubiquinol to cytochrome c	5.26E-04	7
ion transmembrane transport	1.93E-03	22
cation transport	2.64E-03	25
cellular respiration	6.63E-03	18
generation of precursor metabolites and energy	6.69E-03	25
cytoplasmic translation	9.85E-03	23
oxidation-reduction process	1.55E-02	43
energy coupled proton transport, down electrochemical gradient	2.43E-02	7
ATP synthesis coupled proton transport	2.43E-02	7

Table S17. Genes using only AGA/AGG codons to encode arginine.

Systematic Name	Standard Name	Description
Q0045	COX1	Cytochrome c OXidase
Q0065	AI4	
Q0070	AI5_ALPHA	
Q0080	ATP8	ATP synthase
Q0085	ATP6	ATP synthase
Q0105	COB	CytochrOme B
Q0110	BI2	
Q0130	OLI1	OLIgomycin resistance
Q0250	COX2	Cytochrome c OXidase
Q0275	COX3	Cytochrome c OXidase
YAL003W	EFB1	Elongation Factor Beta
YAL068C	PAU8	seriPAUperin
YAR020C	PAU7	seriPAUperin family
YAR061W		
YAR062W		
YAR068W		
YAR069C		
YBL002W	HTB2	Histone h Two B
YBL003C	HTA2	Histone h Two A
YBL026W	LSM2	Like SM
YBL036C		

YBL053W		
YBL064C	PRX1	PeroxiRedoXin
YBL071W-A	KTI11	Kluveromyces lactis Toxin Insensitive
YBR013C		
YBR014C	GRX7	GlutaRedoXin
YBR057C	MUM2	MUddled Meiosis
YBR067C	TIP1	Temperature shock-Inducible Protein
YBR077C	SLM4	Synthetic Lethal with Mss4
YBR078W	ECM33	ExtraCellular Mutant
YBR111W-A	SUS1	Sl gene Upstream of ySa1
YBR118W	TEF2	Translation Elongation Factor
YBR124W		
YBR162C	TOS1	Target Of Sbf
YBR162W-A	YSY6	
YBR210W	ERV15	ER Vesicle Protein
YBR233W-A	DAD3	Duo1 And Dam1 interacting
YBR244W	GPX2	Glutathione PeroXidase
YBR249C	ARO4	AROmatic amino acid requiring
YBR301W	PAU24	seriPAUperin family
YCL022C		
YCL035C	GRX1	GlutaRedoXin
YCL043C	PDI1	Protein Disulfide Isomerase
YCR004C	YCP4	
YCR020W-B	HTL1	High-Temperature Lethal
YCR024C	SLM5	Synthetic Lethal with Mss4
YCR024C-A	PMP1	Plasma Membrane Proteolipid
YCR043C		
YCR071C	IMG2	Integrity of Mitochondrial Genome
YCR083W	TRX3	ThioRedoXin
YCR104W	PAU3	seriPAUperin family
YDL045W-A	MRP10	Mitochondrial Ribosomal Protein
YDL050C		
YDL067C	COX9	Cytochrome c OXidase
YDL125C	HNT1	Histidine triad NucleoTide-binding
YDL136W	RPL35B	Ribosomal Protein of the Large subunit
YDL191W	RPL35A	Ribosomal Protein of the Large subunit
YDL208W	NHP2	Non-Histone Protein

YDL212W	SHR3	Super high Histidine Resistant
YDL219W	DTD1	D-Tyr-tRNA(Tyr) Deacylase
YDL228C		
YDL241W		
YDR002W	YRB1	Yeast Ran Binder
YDR008C		
YDR016C	DAD1	Duo1 And Dam1 interacting
YDR050C	TPI1	Triose-Phosphate Isomerase
YDR079C-A	TFB5	
YDR086C	SSS1	Sec Sixty-one Suppressor
YDR100W	TVP15	Tlg2-Vesicle Protein
YDR139C	RUB1	Related to UBiquitin
YDR156W	RPA14	RNA Polymerase A
YDR178W	SDH4	Succinate DeHydrogenase
YDR192C	NUP42	NUclear Pore
YDR193W		
YDR203W		
YDR224C	HTB1	Histone h Two B
YDR225W	HTA1	Histone h Two A
YDR233C	RTN1	ReTiculoN-like
YDR252W	BTT1	BTf Three
YDR298C	ATP5	ATP synthase
YDR322C-A	TIM11	Translocase of the Inner Mitochondrial membrane
YDR357C	CNL1	CNo-Like
YDR373W	FRQ1	FReQuenin homolog
YDR382W	RPP2B	Ribosomal Protein P2 Beta
YDR383C	NKP1	Non-essential Kinetochore Protein
YDR424C	DYN2	DYNein
YDR437W	GPI19	Glycosyl PhosphatidylInositol anchor biosynthesis
YDR441C	APT2	Adenine PhosphoribosylTransferase
YDR453C	TSA2	Thiol-Specific Antioxidant
YDR455C		
YDR510W	SMT3	Suppressor of Mif Two
YDR513W	GRX2	GlutaRedoXin
YDR519W	FPR2	FKBP Proline Rotamase
YDR529C	QCR7	ubiQuinol-cytochrome C oxidoReductase
YDR530C	APA2	AP4A phosphorylase

YDR542W	PAU10	seriPAUperin
YEL001C	IRC22	Increased Recombination Centers
YEL003W	GIM4	Gene Involved in Microtubule biogenesis
YEL017C-A	PMP2	Plasma Membrane Proteolipid
YEL027W	VMA3	Vacuolar Membrane Atpase
YEL034W	HYP2	HYPusine-containing protein
YEL039C	CYC7	CYtochrome C
YEL049W	PAU2	seriPAUperin family
YER011W	TIR1	TIp1-Related
YER048W-A	ISD11	Iron-Sulfur protein biogenesis, Desulfurase-interacting protein
YER057C	HMF1	Homologous Mmf1p Factor
YER071C	TDA2	Topoisomerase I Damage Affected
YER074W-A	YOS1	Yip One Suppressor
YER131W	RPS26B	Ribosomal Protein of the Small subunit
YER150W	SPI1	Stationary Phase Induced
YER159C	BUR6	Bypass UAS Requirement
YFL020C	PAU5	seriPAUperin family
YFL026W	STE2	STErile
YFL034C-A	RPL22B	Ribosomal Protein of the Large subunit
YFL034C-B	MOB2	Mps One Binder
YFL045C	SEC53	SECretory
YFR011C	MIC19	MItochondrial contact site and Cristae organizing system
YFR026C	ULI1	Upr-L-Inducible gene
YFR032C-A	RPL29	Ribosomal Protein of the Large subunit
YFR033C	QCR6	ubiQuinol-cytochrome C oxidoReductase
YFR044C	DUG1	Deficient in Utilization of Glutathione
YFR052W	RPN12	Regulatory Particle Non-ATPase
YGL030W	RPL30	Ribosomal Protein of the Large subunit
YGL054C	ERV14	ER Vesicle
YGL089C	MF(ALPHA)2	Mating Factor ALPHA
YGL106W	MLC1	Myosin Light Chain
YGL127C	SOH1	Suppressor Of Hpr1
YGL189C	RPS26A	Ribosomal Protein of the Small subunit
YGL214W		
YGL226C-A	OST5	OligoSaccharylTransferase
YGL239C		

YGL242C		
YGL258W	VEL1	VELum formation
YGL261C	PAU11	seriPAUperin
YGR018C		
YGR022C		
YGR024C	THG1	tRNAHis Guanylyltransferase
YGR027C	RPS25A	Ribosomal Protein of the Small subunit
YGR043C	NQM1	Non-Quiescent Mutant
YGR063C	SPT4	SuPpressor of Ty's
YGR106C	VOA1	V0 Assembly protein
YGR181W	TIM13	Translocase of the Inner Mitochondrial membrane
YGR185C	TYS1	TYrosyl-tRNA Synthetase
YGR189C	CRH1	Congo Red Hypersensitive
YGR192C	TDH3	Triose-phosphate DeHydrogenase
YGR209C	TRX2	ThioRedoXin
YGR219W		
YGR228W		
YGR232W	NAS6	Non-ATPase Subunit 6
YGR236C	SPG1	Stationary Phase Gene
YGR275W	RTT102	Regulator of Ty1 Transposition
YGR284C	ERV29	ER Vesicle
YGR290W		
YGR294W	PAU12	seriPAUperin
YHL015W	RPS20	Ribosomal Protein of the Small subunit
YHL033C	RPL8A	Ribosomal Protein of the Large subunit
YHL034C	SBP1	
YHL046C	PAU13	seriPAUperin
YHR001W	OSH7	OxySterol binding protein Homolog
YHR001W-A	QCR10	ubiQuinol-cytochrome C oxidoReductase
YHR005C-A	TIM10	Translocase of the Inner Membrane
YHR008C	SOD2	SuperOxide Dismutase
YHR021C	RPS27B	Ribosomal Protein of the Small subunit
YHR029C	YHI9	
YHR039C-A	VMA10	
YHR049W	FSH1	Family of Serine Hydrolases
YHR051W	COX6	Cytochrome c OXidase
YHR052W	CIC1	Core Interacting Component

YHR072W-A	NOP10	NucleOlar Protein
YHR076W	PTC7	Phosphatase type Two C
YHR079C-A	SAE3	Sporulation in the Absence of spo Eleven
YHR087W	RTC3	Restriction of Telomere Capping
YHR121W	LSM12	Like SM
YHR175W	CTR2	Copper TRansport
YHR193C	EGD2	Enhancer of Gal4 DNA binding
YHR213W		
YHR214W		
YIL003W	CFD1	Cytosolic Fe-S cluster Deficient
YIL008W	URM1	Ubiquitin Related Modifier
YIL011W	TIR3	TIp1-Related
YIL027C	EMC5	ER Membrane protein Complex
YIL051C	MMF1	Mitochondrial Matrix Factor
YIL138C	TPM2	TroPoMyosin
YIL167W	SDL1	
YIL176C	PAU14	seriPAUperin
YIR012W	SQT1	
YIR019C	FLO11	FLOcculation
YIR022W	SEC11	SECretory
YIR035C		putative Novel REductase
YIR041W	PAU15	seriPAUperin
YJL032W		
YJL041W	NSP1	NucleoSkeletal-like Protein
YJL052W	TDH1	Triose-phosphate DeHydrogenase
YJL066C	MPM1	Mitochondrial Peculiar Membrane protein
YJL122W	ALB1	Arx1 Little Brother
YJL158C	CIS3	CIk1 Suppressing
YJL166W	QCR8	ubiQuinol-cytochrome C oxidoReductase
YJL179W	PFD1	PreFolDin
YJL223C	PAU1	seriPAUperin family
YJR009C	TDH2	Triose-phosphate DeHydrogenase
YJR047C	ANB1	ANaeroBically induced
YJR048W	CYC1	CYtochrome C
YJR070C	LIA1	Ligand of eIF5A
YJR071W		
YJR079W		

YKL018W	SWD2	Set1c, WD40 repeat protein
YKL036C		
YKL120W	OAC1	OxaloAcetate Carrier
YKL136W		
YKL152C	GPM1	Glycerate PhosphoMutase
YKL153W		
YKL156W	RPS27A	Ribosomal Protein of the Small subunit
YKL163W	PIR3	Protein containing Internal Repeats
YKL164C	PIR1	Protein containing Internal Repeats
YKL177W		
YKL207W	EMC3	ER Membrane protein Complex
YKL224C	PAU16	seriPAUperin
YKR033C		
YKR092C	SRP40	Serine Rich Protein
YLL025W	PAU17	seriPAUperin
YLL030C	RRT7	Regulator of rDNA Transcription
YLL039C	UBI4	Ubiquitin
YLL044W		
YLL045C	RPL8B	Ribosomal Protein of the Large subunit
YLL050C	COF1	COFilin
YLL064C	PAU18	seriPAUperin
YLR008C	PAM18	Presequence translocase-Associated Motor
YLR021W	IRC25	Increased Recombination Centers
YLR037C	PAU23	seriPAUperin family
YLR038C	COX12	Cytochrome c OXidase
YLR042C		
YLR061W	RPL22A	Ribosomal Protein of the Large subunit
YLR062C	BUD28	BUD site selection
YLR066W	SPC3	Signal Peptidase Complex
YLR068W	FYV7	Function required for Yeast Viability
YLR109W	AHP1	Alkyl HydroPeroxide reductase
YLR167W	RPS31	Ribosomal Protein of the Small subunit
YLR179C		
YLR232W		
YLR244C	MAP1	Methionine AminoPeptidase
YLR262C	YPT6	Yeast Protein Two
YLR268W	SEC22	SECretory

YLR295C	ATP14	ATP synthase
YLR321C	SFH1	Snf Five Homolog
YLR325C	RPL38	Ribosomal Protein of the Large subunit
YLR327C	TMA10	Translation Machinery Associated
YLR333C	RPS25B	Ribosomal Protein of the Small subunit
YLR354C	TAL1	TransALdolase
YLR364W	GRX8	GlutaRedoXin
YLR395C	COX8	Cytochrome c OXidase
YLR441C	RPS1A	Ribosomal Protein of the Small subunit
YLR461W	PAU4	seriPAUperin family
YML028W	TSA1	Thiol-Specific Antioxidant
YML063W	RPS1B	Ribosomal Protein of the Small subunit
YML079W		
YML094W	GIM5	Gene Involved in Microtubule biogenesis
YML106W	URA5	URAcil requiring
YML108W		
YMR010W		
YMR071C	TVP18	Tlg2-Vesicle Protein
YMR149W	SWP1	Suppressor of a WbP1 mutation
YMR175W	SIP18	Salt Induced Protein
YMR181C		
YMR184W	ADD37	Alpha1-proteinase inhibitor-Degradation Deficient
YMR202W	ERG2	ERGosterol biosynthesis
YMR230W	RPS10B	Ribosomal Protein of the Small subunit
YMR256C	COX7	Cytochrome c OXidase
YMR303C	ADH2	Alcohol DeHydrogenase
YMR325W	PAU19	seriPAUperin
YNL001W	DOM34	Duplication Of Multilocus region
YNL015W	PBI2	Proteinase B Inhibitor
YNL079C	TPM1	TroPoMyosin
YNL131W	TOM22	Translocase of the Outer Mitochondrial membrane
YNL145W	MFA2	Mating Factor A
YNR003C	RPC34	RNA Polymerase C
YNR076W	PAU6	seriPAUperin family
YOL039W	RPP2A	Ribosomal Protein P2 Alpha
YOL052C-A	DDR2	DNA Damage Responsive
YOL077W-A	ATP19	ATP synthase

YOL086C	ADH1	Alcohol DeHydrogenase
YOL099C		
YOL127W	RPL25	Ribosomal Protein of the Large subunit
YOL133W	HRT1	High level expression Reduces Ty3 transposition
YOL134C		
YOL150C		
YOL161C	PAU20	seriPAUperin
YOL163W		
YOR007C	SGT2	Small Glutamine-rich Tetratricopeptide repeat-containing protein
YOR009W	TIR4	TIp1-Related
YOR010C	TIR2	TIp1-Related
YOR013W	IRC11	Increased Recombination Centers
YOR045W	TOM6	Translocase of the Outer Mitochondrial membrane
YOR055W		
YOR068C	VAM10	VAcuolar Morphogenesis
YOR122C	PFY1	ProFilin of Yeast
YOR131C		
YOR157C	PUP1	PUtative Proteasome subunit
YOR159C	SME1	
YOR164C	GET4	Guided Entry Tail-anchored proteins
YOR170W		
YOR210W	RPB10	RNA Polymerase B
YOR232W	MGE1	Mitochondrial GrpE
YOR265W	RBL2	Rescues Beta-tubulin Lethality
YOR277C		
YOR293W	RPS10A	Ribosomal Protein of the Small subunit
YOR382W	FIT2	Facilitator of Iron Transport
YOR387C		
YPL037C	EGD1	Enhancer of Gal4 DNA binding
YPL048W	CAM1	Calcium And Membrane-binding protein
YPL052W	OAZ1	Ornithine decarboxylase AntiZyme
YPL069C	BTS1	Bet Two Suppressor
YPL135W	ISU1	IScU homolog
YPL142C		
YPL163C	SVS1	Suppressor of Vanadate Sensitivity
YPL187W	MF(ALPHA)1	Mating Factor ALPHA

YPL200W	CSM4	Chromosome Segregation in Meiosis
YPL211W	NIP7	Nuclear ImPort
YPL234C	VMA11	Vacuolar Membrane Atpase
YPR020W	ATP20	ATP synthase
YPR028W	YOP1	YIP One Partner
YPR065W	ROX1	Regulation by OXygen
YPR080W	TEF1	Translation Elongation Factor
YPR133W-A	TOM5	Translocase of the Outer Mitochondrial membrane
YPR153W		genetic interaction profile similarity to MTC Annotated Yeast genes MTC2 and MTC4
YPR193C	HPA2	Histone and other Protein Acetyltransferase

Supplemental Figures

Figure S1

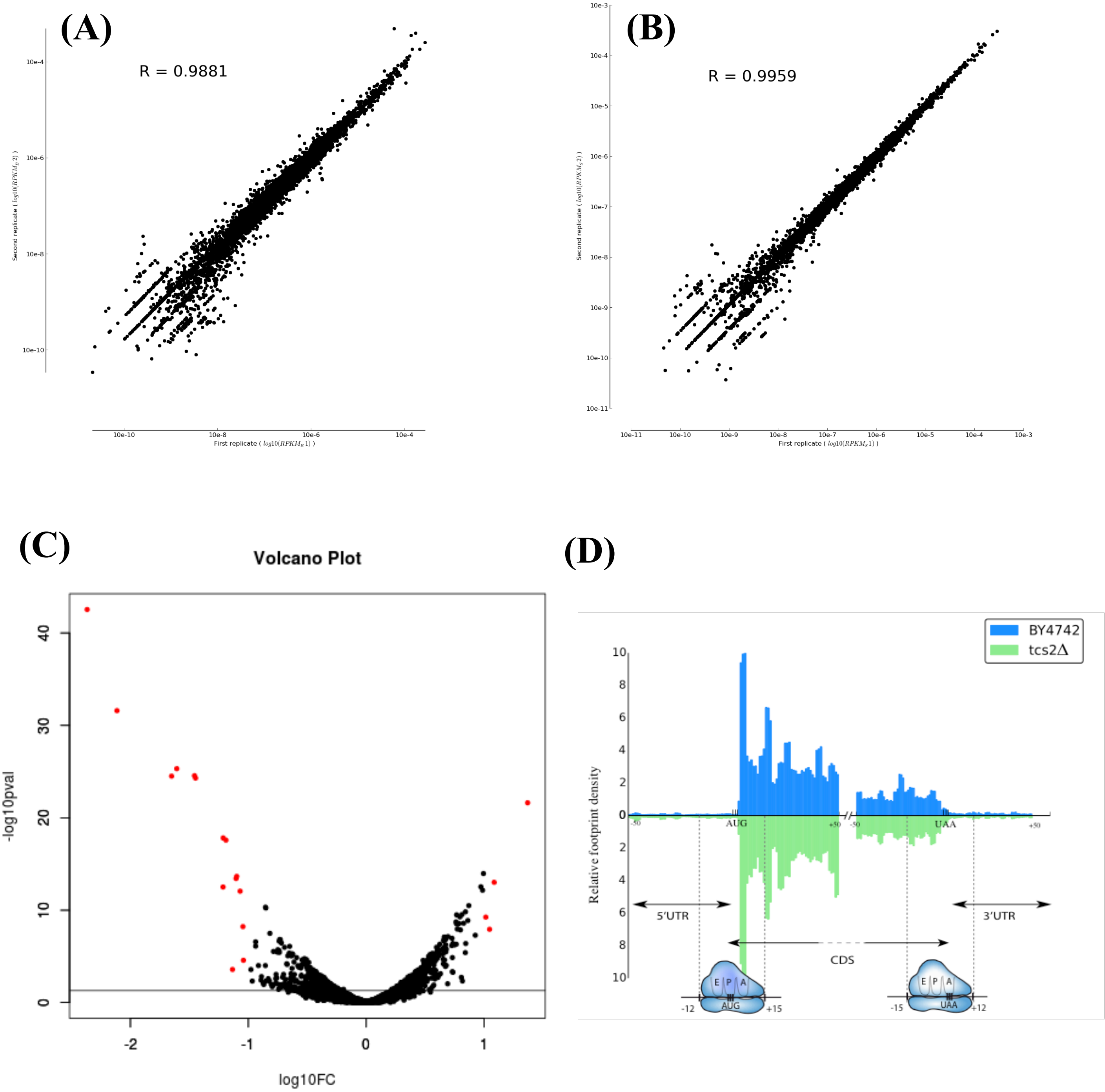

Figure S1. RPKM of biological replicates (A) BY4742 (B) *tcs2*Δ. (C) Differential expression of RPFs in *tcs2*Δ. (D) Ribosome profiling fragments map to open reading frames. The RPFs were mapped to a composite transcript extending 50 bases on either side of the start (AUG) and stop (UAA) codons.

Figure S2

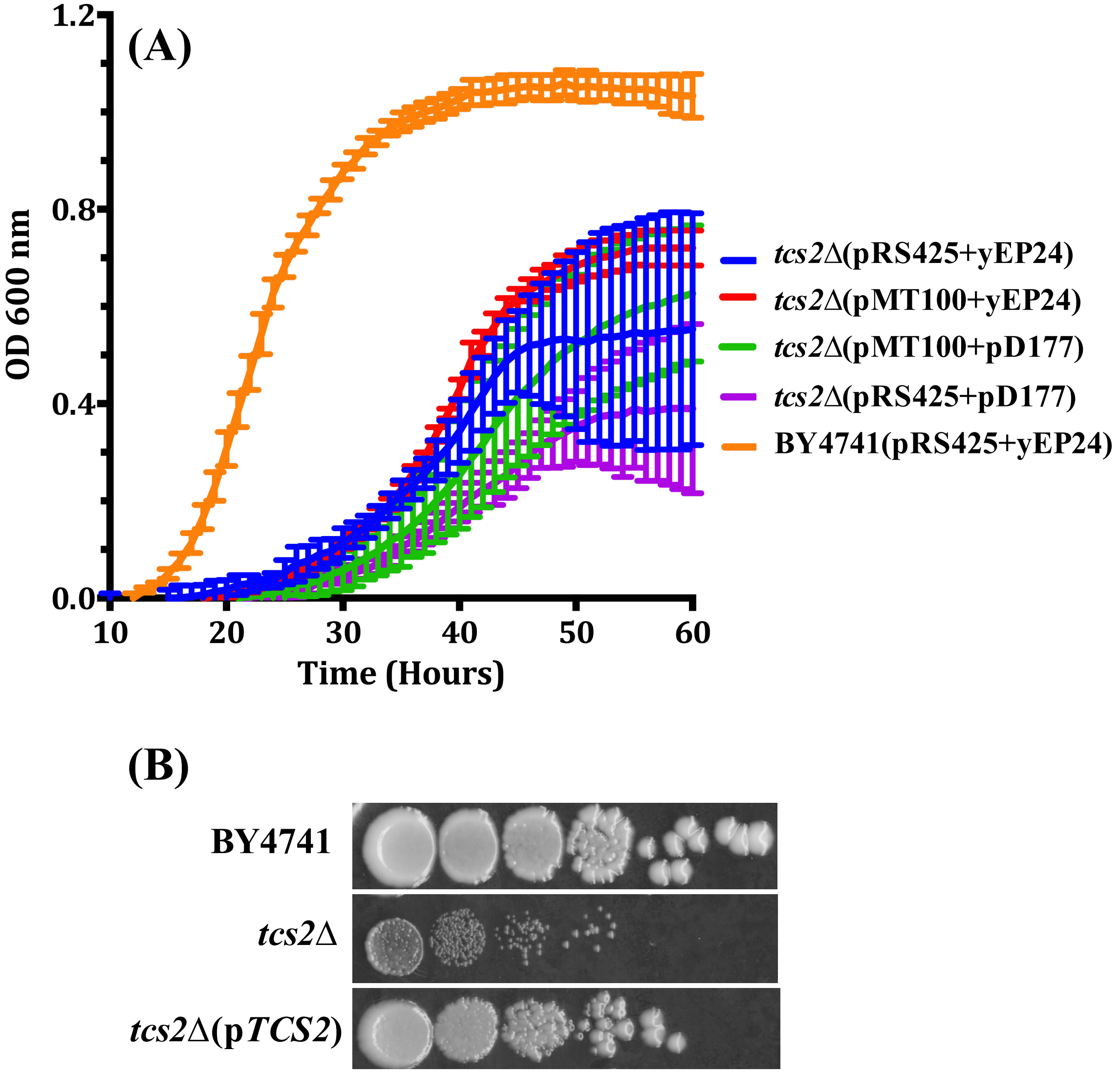

Figure S2. Expression of Ternary Complex (TC) does not suppress slow growth of mutations in t^{6}A. (A) BY4741 and *tsc2*Δ were transformed with plasmids expressing pMT100 (tRNA^{iMet}), pD177 (eIF2α), or pMT100+pD177 (TC). Data points are the average 5 biological replicates. Error bars represent standard error of the mean (SEM). (B) Complementation of *tcs2*Δ with *TCS* expressed *in trans* (pBN204),

Figure S3

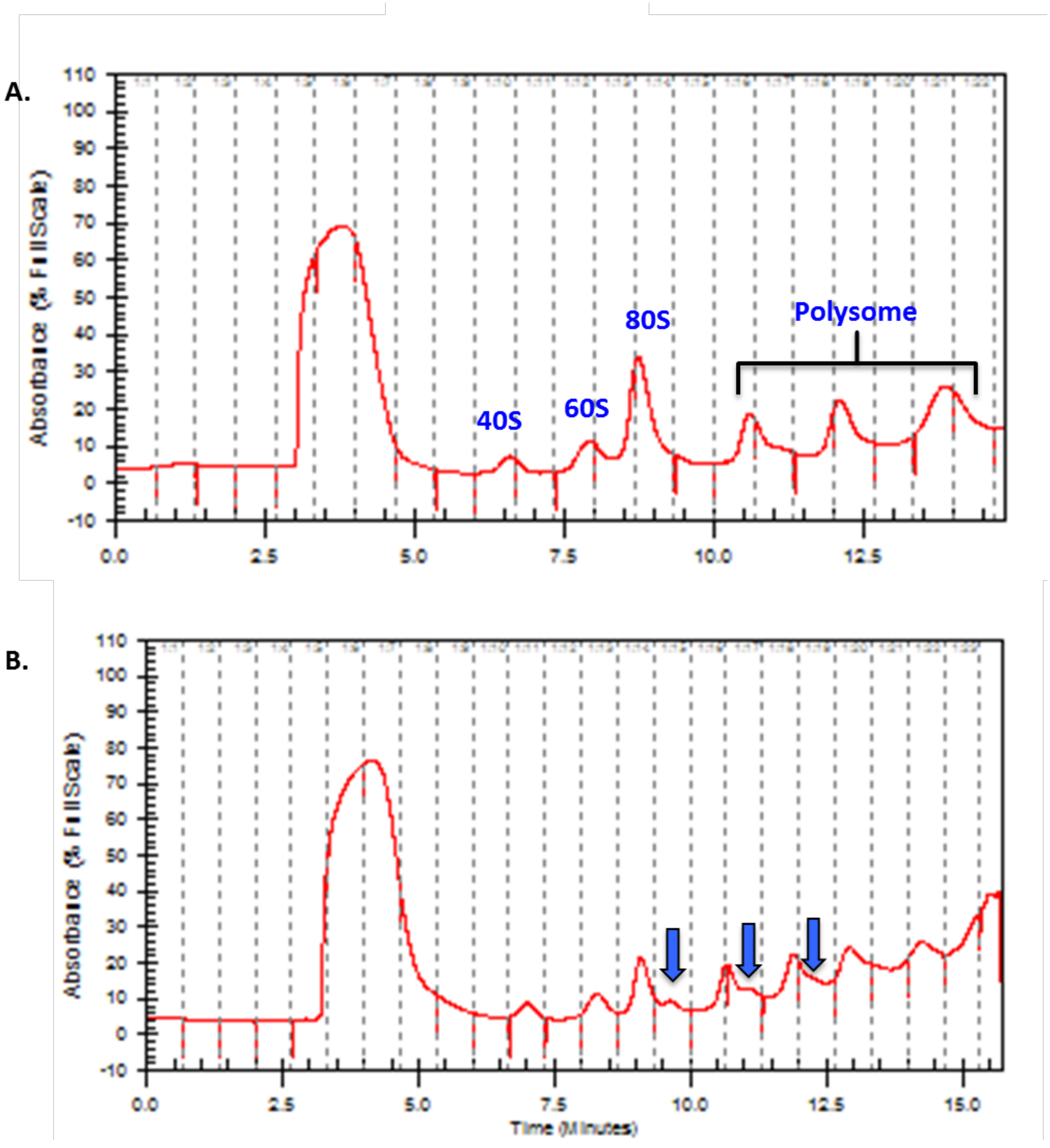

Figure 3. Polysome profiles. A) BY4742. B) *tcs2*Δ. Half-mer phenotype indicated by blue arrows.

Figure S4

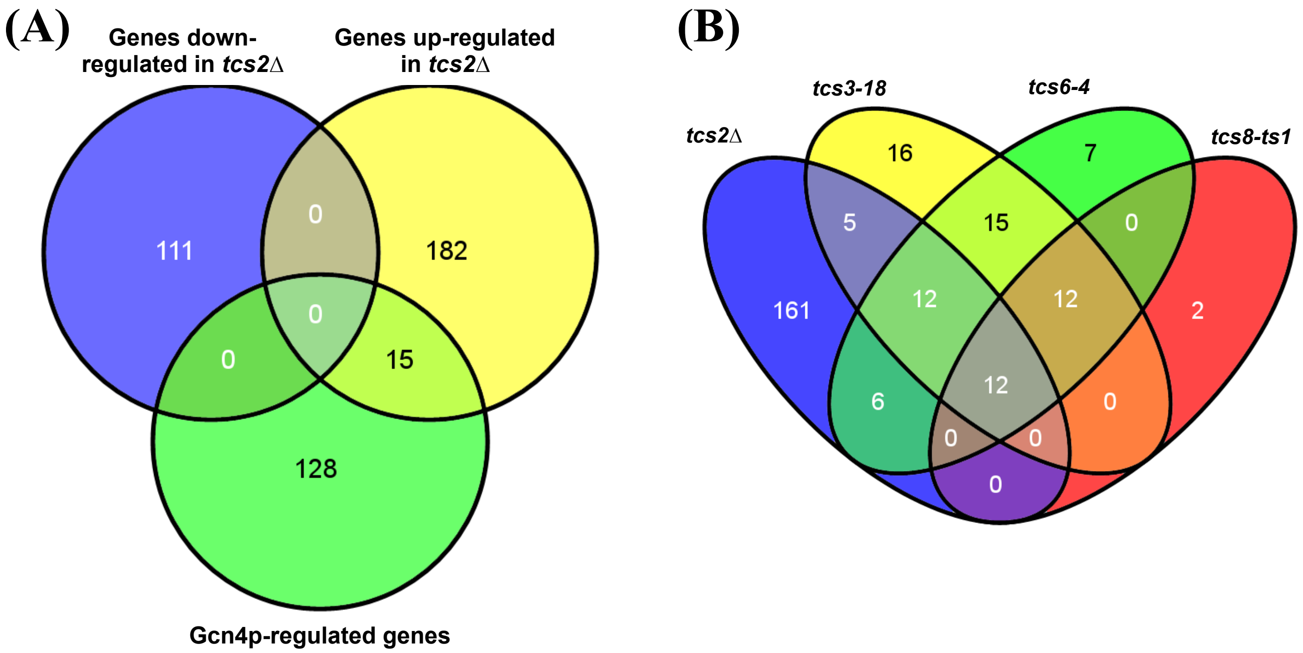

Figure S4. Genes differentially expressed in *tcs2*Δ as measured by ribosome profiling and prior datasets. (A) Gcn4p regulated genes identified by ChIP-Chip (71) differentially expressed in *tcs2*Δ. (B) Overlap between genes up-regulated in *tcs2*Δ as measured by ribosome profiling and genes up-regulated in *tcs3*, *tcs6*, or *tcs8* mutants measured by microarray (30).

Figure S5

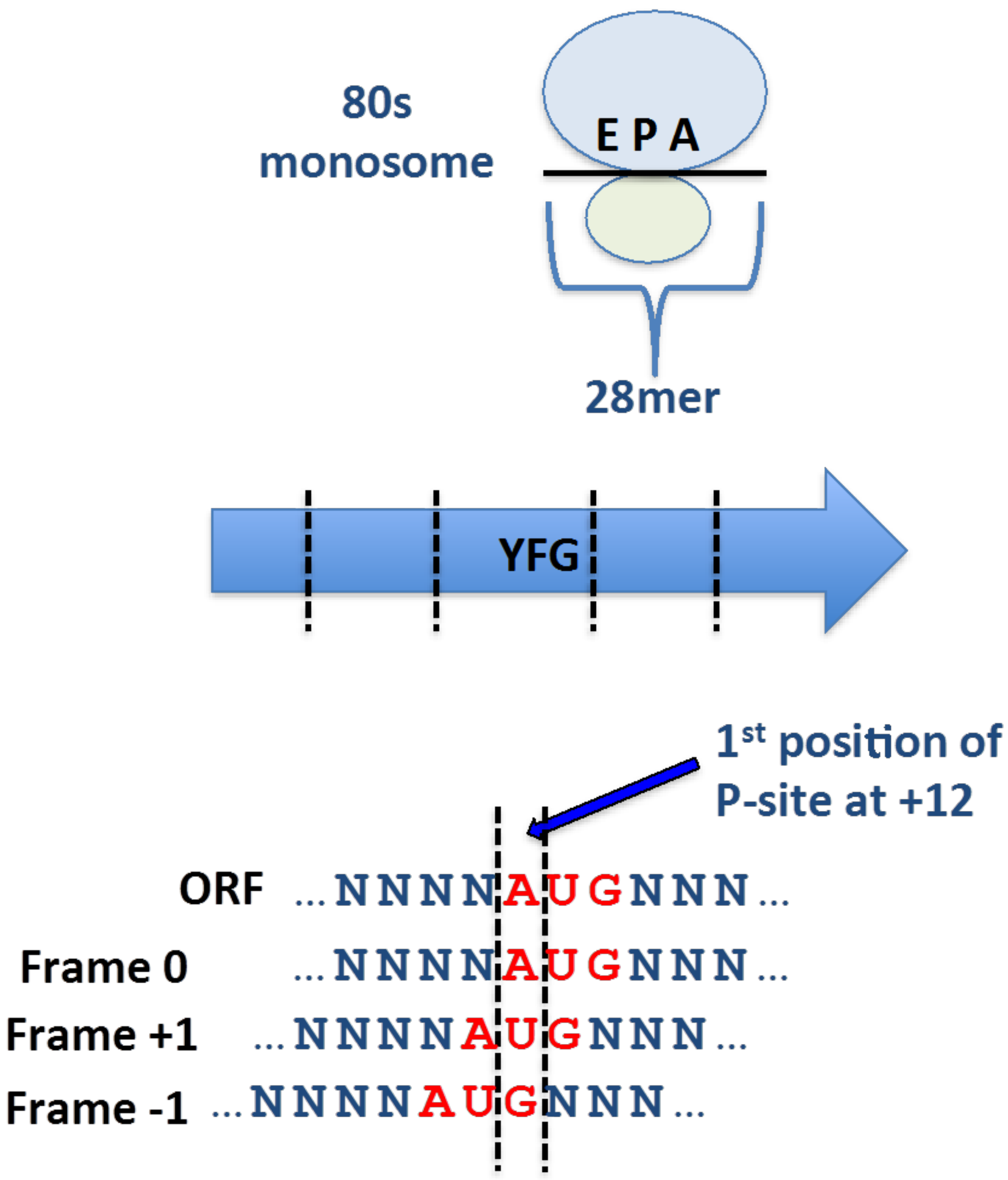

Figure S5. Determining frame of the ribosome. Only unique 28-mers, representing the RPF, are mapped to the genome. The +12 base of the 28-mer corresponds to the first positions of the P-site of the ribosome. The base at the 12th position is used to determine the frame of the ribosome.

Figure S6

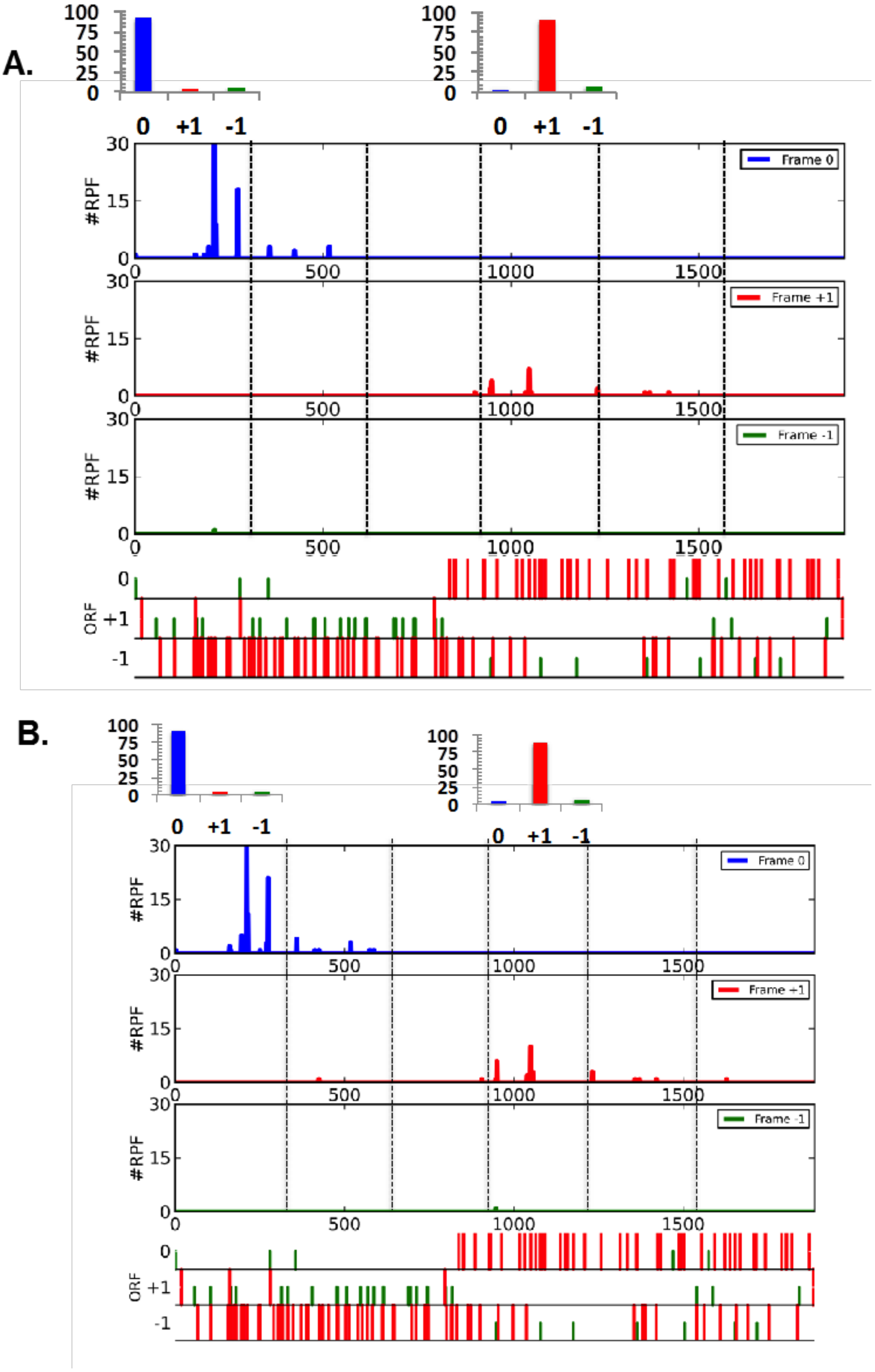

Figure S6. Detection of +1 frame-shifting in *TRM140* using ribosome profiling. Frame-shift must occur at nucleotide 832 to create the full-length and functional Trm140p. Inset graphs indicate the percentage of reads in each frame for that analysis window. Frame 0 = Blue, Frame +1 = red, Frame -1 = Green. Depicted on the bottom of each image is the location of start codons (short, green bars) and stop codons (long red bars). A) BY4742. B) *tcs2*Δ.

Figure S7

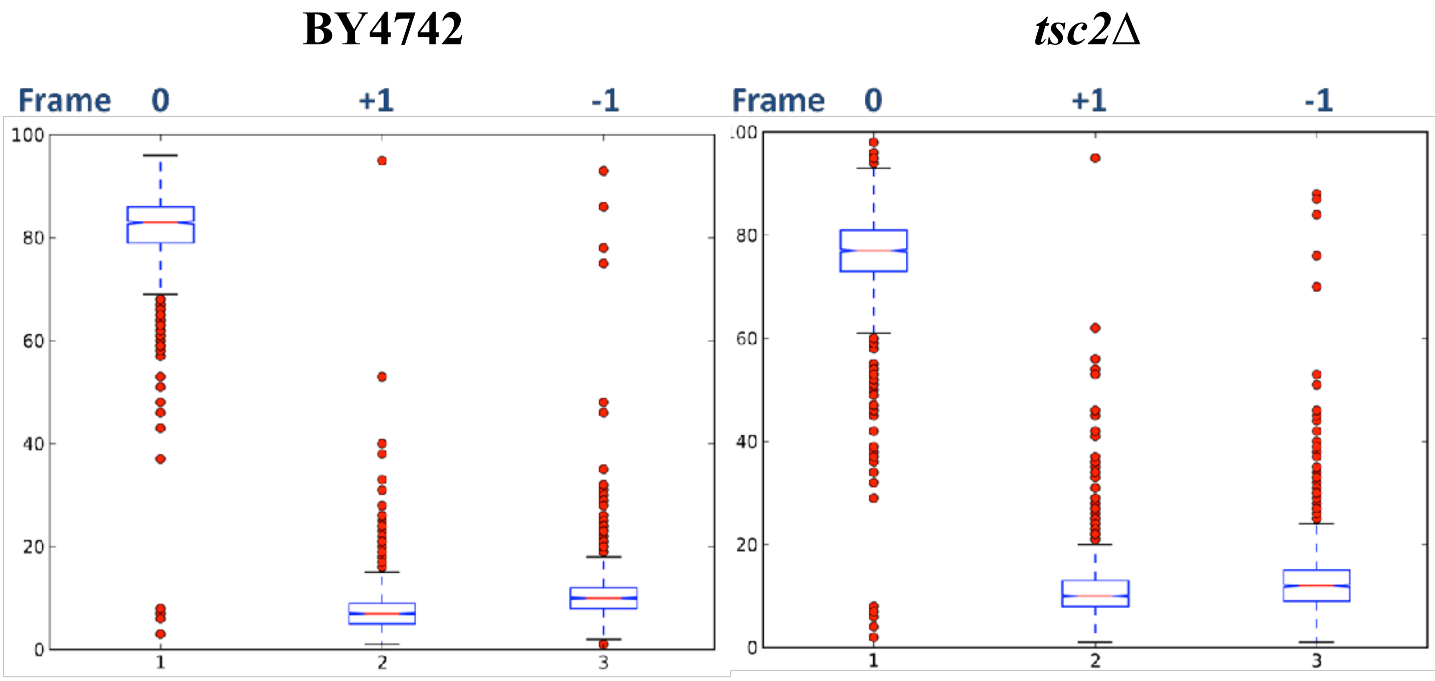

Figure S7. Global analysis of frame-shifts. Left panel, BY4742; Right panel, *tcs2*Δ. Summing all reads used to determine frame indicates that 80% of all reads from ribosome profiling are in the correct, annotated frame. There is a significant difference in frame shifting between wild type and mutant (*P* = 6.5 X 10-98, t-test).

Figure S8

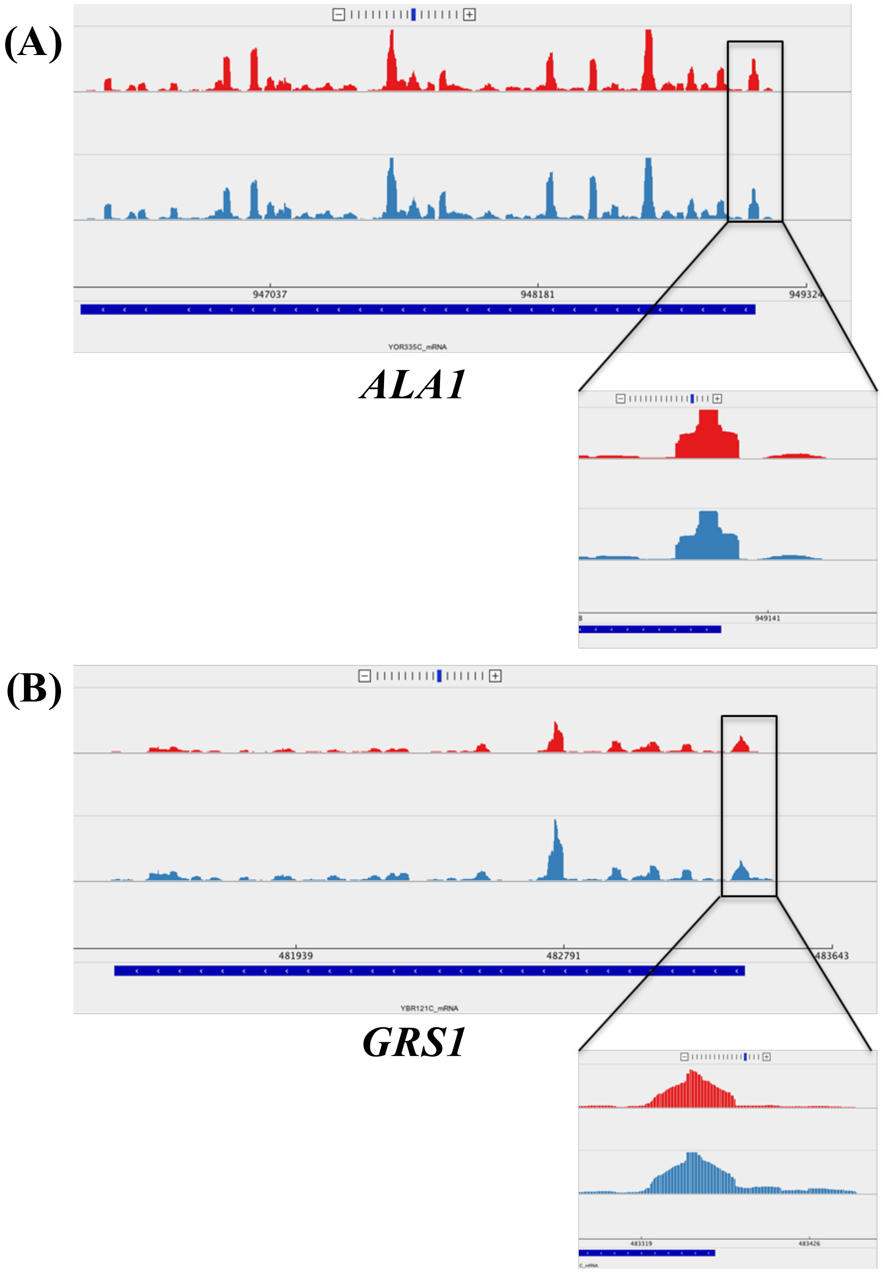

Figure S8. Detection of upstream non-AUG starts. A) *ALA1* B) *GRS1*. For both images, BY4742 is plotted in red, and *tcs2*Δ is plotted in blue. Both genes are on the Crick strand. The inset shows the 5’ end of the gene and the region upstream the annotated AUG start.

REFERENCES

1. Thiaville PC, El Yacoubi B, Perrochia L, Hecker A, Prigent M, Thiaville JJ, Forterre P, Namy O, Basta T, and de Crécy-Lagard V (**2014**). Cross Kingdom Functional Conservation of the Core Universally Conserved Threonylcarbamoyladenosine tRNA Synthesis Enzymes. **Eukaryot Cell** 13(9): 1222–1231. doi: 10.1128/EC.00147-14.

2. El Yacoubi B, Hatin I, Deutsch C, Kahveci T, Rousset J-P, Iwata-Reuyl D, Murzin AG, and de Crécy-Lagard V (**2011**). A role for the universal Kae1/Qri7/YgjD (COG0533) family in tRNA modification. **EMBO J** 30(5): 882–893. doi: 10.1038/emboj.2010.363.

3. Christianson TW, Sikorski RS, Dante M, Shero JH, and Hieter P (**1992**). Multifunctional yeast high-copy-number shuttle vectors. **Gene** 110(1): 119–122. doi:http://www.ncbi.nlm.nih.gov/pubmed/1544568.

4. Leidel S, Pedrioli PG a, Bucher T, Brost R, Costanzo M, Schmidt A, Aebersold R, Boone C, Hofmann K, and Peter M (**2009**). Ubiquitin-related modifier Urm1 acts as a sulphur carrier in thiolation of eukaryotic transfer RNA. **Nature** 458(7235): 228–232. doi: 10.1038/nature07643.

5. El Yacoubi B, Lyons B, Cruz Y, Reddy R, Nordin B, Agnelli F, Williamson JR, Schimmel P, Swairjo MA, and de Crécy-Lagard V (**2009**). The universal YrdC/Sua5 family is required for the formation of threonylcarbamoyladenosine in tRNA. **Nucleic Acids Res** 37(9): 2894–2909. doi: 10.1093/nar/gkp152.

6. Dever TE, Yang W, Aström S, Byström AS, and Hinnebusch AG (**1995**). Modulation of tRNA(iMet), eIF-2, and eIF-2B expression shows that GCN4 translation is inversely coupled to the level of eIF-2.GTP.Met-tRNA(iMet) ternary complexes. **Mol Cell Biol** 15(11): 6351–6363. doi:http://www.pubmedcentral.nih.gov/articlerender.fcgi?artid=230887&tool=pmcentrez&rendertype=abstract.

7. Hill JE, Myers a M, Koerner TJ, and Tzagoloff a (**1986**). Yeast/E. coli shuttle vectors with multiple unique restriction sites. **Yeast** 2(3): 163–167. doi: 10.1002/yea.320020304.
